# A sex-informed approach to improve the personalised decision making process in myelodysplastic syndromes: a multicentre, observational cohort study

**DOI:** 10.1016/S2352-3026(22)00323-4

**Published:** 2022-11-24

**Authors:** GenoMed4All consortium, Giulia Maggioni, Matteo Bersanelli, Erica Travaglino, Ana Alfonso Piérola, Annika Kasprzak, Arnan Sangerman Montserrat, Elisabetta Sauta, Claudia Sala, Tommaso Matteuzzi, Manja Meggendorfer, Matteo Gnocchi, Lin-Pierre Zhao, Cristina Astrid Tentori, Kathrin Nachtkamp, Daniele Dall'Olio, Ettore Mosca, Marta Ubezio, Alessia Campagna, Antonio Russo, Giulia Rivoli, Massimo Bernardi, Lorenza Borin, Maria Teresa Voso, Marta Riva, Esther Oliva, Matteo Zampini, Elena Riva, Elena Saba, Saverio D'Amico, Luca Lanino, Benedetta Tinterri, Francesca Re, Marilena Bicchieri, Laura Giordano, Giovanni Angelotti, Pierandrea Morandini, Anne Sophie Kubasch, Francesco Passamonti, Alessandro Rambaldi, Victor Savevski, Armando Santoro, Arjan A. van de Loosdrecht, Alice Brogi, Valeria Santini, Shahram Kordasti, Guillermo Sanz, Francesc Sole, Norbert Gattermann, Wolfgang Kern, Uwe Platzbecker, Lionel Ades, Pierre Fenaux, Torsten Haferlach, Gastone Castellani, Ulrich Germing, Maria Diez-Campelo, Matteo G. Della Porta

## Abstract

**Background:**

Sex is a major source of diversity among patients and a sex-informed approach is becoming a new paradigm in precision medicine. We aimed to describe sex diversity in myelodysplastic syndromes in terms of disease genotype, phenotype, and clinical outcome. Moreover, we sought to incorporate sex information into the clinical decision-making process as a fundamental component of patient individuality.

**Methods:**

In this multicentre, observational cohort study, we retrospectively analysed 13 284 patients aged 18 years or older with a diagnosis of myelodysplastic syndrome according to 2016 WHO criteria included in the EuroMDS network (n=2025), International Working Group for Prognosis in MDS (IWG-PM; n=2387), the Spanish Group of Myelodysplastic Syndromes registry (GESMD; n=7687), or the Düsseldorf MDS registry (n=1185). Recruitment periods for these cohorts were between 1990 and 2016. The correlation between sex and genomic features was analysed in the EuroMDS cohort and validated in the IWG-PM cohort. The effect of sex on clinical outcome, with overall survival as the main endpoint, was analysed in the EuroMDS population and validated in the other three cohorts. Finally, novel prognostic models incorporating sex and genomic information were built and validated, and compared to the widely used revised International Prognostic Scoring System (IPSS-R). This study is registered with ClinicalTrials.gov, NCT04889729.

**Findings:**

The study included 7792 (58·7%) men and 5492 (41·3%) women. 10 906 (82·1%) patients were White, and race was not reported for 2378 (17·9%) patients. Sex biases were observed at the single-gene level with mutations in seven genes enriched in men (*ASXL1, SRSF2*, and *ZRSR2* p<0·0001 in both cohorts; *DDX41* not available in the EuroMDS cohort *vs* p=0·0062 in the IWG-PM cohort; *IDH2* p<0·0001 in EuroMDS *vs* p=0·042 in IWG-PM; *TET2* p=0·031 *vs* p=0·035; *U2AF1* p=0·033 *vs* p<0·0001) and mutations in two genes were enriched in women (*DNMT3A* p<0·0001 in EuroMDS *vs* p=0·011 in IWG-PM; *TP53* p=0·030 *vs* p=0·037). Additionally, sex biases were observed in co-mutational pathways of founding genomic lesions (splicing-related genes, predominantly in men, p<0·0001 in both the EuroMDS and IWG-PM cohorts), in DNA methylation (predominantly in men, p=0·046 in EuroMDS *vs* p<0·0001 in IWG-PM), and *TP53* mutational pathways (predominantly in women, p=0·0073 in EuroMDS *vs* p<0·0001 in IWG-PM). In the retrospective EuroMDS cohort, men had worse median overall survival (81·3 months, 95% CI 70·4–95·0 in men *vs* 123·5 months, 104·5–127·5 in women; hazard ratio [HR] 1·40, 95% CI 1·26–1·52; p<0·0001). This result was confirmed in the prospective validation cohorts (median overall survival was 54·7 months, 95% CI 52·4–59·1 in men *vs* 74·4 months, 69·3–81·2 in women; HR 1·30, 95% CI 1·23–1·35; p<0·0001 in the GEMSD MDS registry; 40·0 months, 95% CI 33·4–43·7 in men *vs* 54·2 months, 38·6–63·8 in women; HR 1·23, 95% CI 1·08–1·36; p<0·0001 in the Dusseldorf MDS registry). We developed new personalised prognostic tools that included sex information (the sex-informed prognostic scoring system and the sex-informed genomic scoring system). Sex maintained independent prognostic power in all prognostic systems; the highest performance was observed in the model that included both sex and genomic information. A five-to-five mapping between the IPSS-R and new score categories resulted in the re-stratification of 871 (43·0%) of 2025 patients from the EuroMDS cohort and 1003 (42·0%) of 2387 patients from the IWG-PM cohort by using the sex-informed prognostic scoring system, and of 1134 (56·0%) patients from the EuroMDS cohort and 1265 (53·0%) patients from the IWG-PM cohort by using the sex-informed genomic scoring system. We created a web portal that enables outcome predictions based on a sex-informed personalised approach.

**Interpretation:**

Our results suggest that a sex-informed approach can improve the personalised decision making process in patients with myelodysplastic syndromes and should be considered in the design of clinical trials including low-risk patients.

**Funding:**

European Union (Horizon 2020 and Transcan programs), Italian Association for Cancer Research, Italian Ministry of Health, and Italian Ministry of University and Research.

**Research in context**

**Evidence before this study**

We searched PubMed for studies published between Jan 1, 2010, and April 1, 2022, relating to study sex diversity in patients with myelodysplastic syndromes, using the search terms “myelodysplastic syndrome”, “sex” (or “gender”), and “gene mutation” or “prognosis”. The search was limited to English-language publications. Few studies specifically described sex diversity in patients with myelodysplastic syndromes in terms of disease genotype and phenotype, and most that did included small patient populations and little information on the disease genomic landscape. A 2021 study by De Morgan and colleagues analysed acute myeloid leukaemia and myelodysplastic syndrome cases from the COSMIC database and Münchner Leukämielabor, Germany, and observed a mutation bias in men, which stems from the preleukaemic stage of the disease and might be related to age-related change in the haematopoietic system (clonal haematopoiesis). Among the 194 studies identified by our search that addressed myelodysplastic syndrome prognostication, we limited our analysis to 13 studies that included large patient populations. Some of these investigations reported that life expectancy in patients with myelodysplastic syndromes differed between men and women, but sex is not included in outcome predictions provided by currently available prognostic scores and is mainly managed as a confounding factor. In a 2010 study by Nösslinger and colleagues of 897 patients receiving supportive care, the inclusion of age and sex and their respective interactions contributed to significantly improving prognostication in patients with myelodysplastic syndromes.

**Added value of this study**

In this study, we aimed to incorporate sex information into the clinical decision making process of patients with myelodysplastic syndromes. By analysing real-world populations and including 13 284 patients, we observed substantial sex-dependent diversity in patients with myelodysplastic syndromes in terms of disease genotype and phenotype. Sex biases were observed at a single-gene level, in co-mutational pathways of founding genomic lesions (splicing-related genes), and in DNA methylation and *TP53* mutational pathways. Moreover, we found evidence that sex has an independent prognostic effect in patients with myelodysplastic syndromes because of several contributing factors, including an impact on the natural course of disease by way of affecting phenotypic features, an increased risk of cardiovascular complications and cardiac death in men, and a differential prognostic effect of the severity of anaemia, observed across all age groups.

**Implications of all the available evidence**

These findings indicate that sex contributes to genomic and clinical heterogeneity in patients with myelodysplastic syndromes. A sex-informed approach might improve the personalised decision making process in these diseases and should be considered in the design of clinical trials. With this aim, we have created a web portal that allows personalised outcome predictions on the basis of a sex-informed approach.

## Introduction

Myelodysplastic syndromes are haematopoietic neoplasms that are characterised by blood cytopenia and an increased risk of evolution into acute myeloid leukemia.^1^ Myelodysplastic syndromes are extremely heterogeneous and therefore a risk-adapted approach is mandatory in their treatment.^1^, ^2^, ^3^ Disease-related risk is assessed by the revised International Prognostic Scoring System (IPSS-R) on the basis of the percentage of bone marrow blasts, blood cytopenia, and cytogenetic abnormalities.^4^

Sex is a major source of diversity among patients in terms of pathophysiology, clinical presentation, prognosis, and treatment response. A new sex-informed approach to precision medicine could refine the decision-making process for various diseases.^5^, ^6^, ^7^, ^8^ There is a strong rationale to study diversity across the sex spectrum in patients with myelodysplastic syndromes. Accumulating evidence has suggested a relationship between sex and disease biology. The incidence of myelodysplastic syndromes increases exponentially after age 60 years and these disorders are more common in men than in women.^1^, ^3^, ^9^ Moreover, a preponderance of myelodysplastic syndromes with del(5q), a disease subtype with a distinct phenotype, prognosis, and treatment, was observed in women.^1^, ^3^, ^9^

From a clinical perspective, life expectancy in the general population differs among men and women, and preliminary observations suggest that the same is true for myelodysplastic syndromes.^10^ In low-risk patients with myelodysplastic syndromes, cardiovascular complications are the leading cause of mortality.^1^, ^3^, ^10^, ^11^, ^12^ We know that the magnitude of the effect of many risk factors for cardiovascular disease strongly differs with sex.^5^, ^6^ Additionally, in patients with myelodysplastic syndromes, a detrimental interaction was reported between anaemia (the pathological hallmark of marrow dysplasia) and cardiovascular comorbidity, thus affecting the individual probability of survival.^12^, ^13^ Overall, these data suggest that the natural history of the disease might be affected by sex-related factors.

In this study, we aimed to describe sex diversity in myelodysplastic syndromes in terms of disease genotype, phenotype, and clinical outcome. Moreover, we sought to incorporate sex information into the clinical decision-making process as a fundamental component of patient individuality.

## Methods

### Study design and participants

In this multicentre, observational cohort study, we retrospectively analysed patients aged 18 years or older who received a conclusive diagnosis of primary myelodysplastic syndrome according to the 2016 WHO classification. Patients with more than 20% bone marrow blasts were excluded.

**Figure 1:**
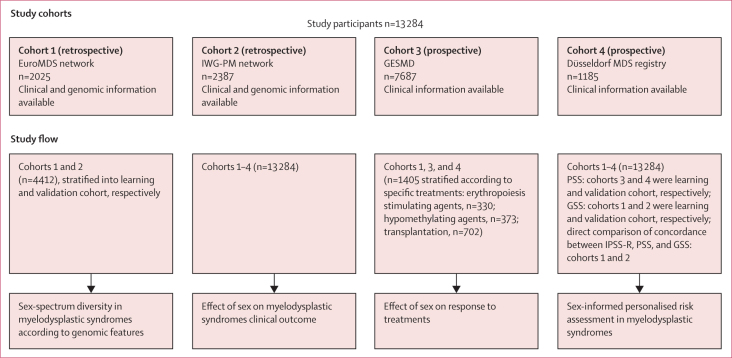
Study cohorts and study flow GESMD=Spanish Group of Myelodysplastic Syndromes registry. GSS=sex-informed genomic scoring system. IPSS-R=revised International Prognostic Scoring System. IWG-PM=International Working Group for Prognosis in MDS. PSS=sex-informed prognostic scoring system.

Patients were included from the following cohorts: the EuroMDS cohort (recruitment period 2001–14, follow-up updated in 2019; n=2025);^14^ the International Working Group for Prognosis in MDS (IWG-PM; recruitment period 1999–2016, follow-up updated in 2016; n=2387);^15^ the Spanish Group of Myelodysplastic Syndromes registry (GESMD; recruitment period 1993–2016, follow-up updated in 2019; n=7687); and the Düsseldorf MDS registry (recruitment period 1990–2016, follow-up updated in 2019; n=1185). Only patients with comprehensive information available on demographics, clinical and haematological features (collected at diagnosis), treatments received, and outcomes were selected for the clinical outcomes analysis (figure 1; appendix 1 pp 2–6).

The Humanitas Ethics Committee approved the study. Written informed consent was obtained from each participant for use of clinical data and samples for genomic screening in all four study cohorts.

### Procedures

For the sex-spectrum diversity in patients with myelodysplastic syndrome according to genomic features analyses, we used the EuroMDS (learning cohort) and IWG-PM cohorts (validation cohort). At diagnosis, cytogenetic analysis was performed using standard G-banding and karyotypes were classified using the International System for Cytogenetic Nomenclature Criteria. Only patients for whom data from the mutational screening of 44 myelodysplastic syndrome-related genes and cytogenetics were available were analysed (2025 in the EuroMDS and 2378 IWG-PM cohorts). Mutational analysis was done on tumour DNA derived from bone marrow mononuclear cells or peripheral blood granulocytes by a targeted sequencing approach. Sequencing strategy was done using a targeted multiplexed amplicon-based approaches (Illumina, San Diego, CA, USA) starting from genomic DNA; the resulting libraries were sequenced on Illumina platforms (NextSeq500) in paired-end mode. For further methodological details on the cytogenetics and mutation screening see appendix 1 (p 11). Targeted regions are listed in appendix 1 (p 12). A list of pathogenic variants is available in appendix 2. Only statistically significant results in both populations were reported (except for *DDX41* mutations, available in the IWG-PM cohort only). Mutations were classified according to the temporal order of acquisitions by a Bradley-Terry model (appendix 1 p 24). To explore sex–age correlations, we analysed the frequency of mutations and their distribution among age groups in men and women (appendix 1 pp 16–23).

For the prognostic analyses, we focused on the retrospective EuroMDS and IWG-PM cohorts, then we provided a clinical validation of the results on the prospective GEMSD and Düsseldorf MDS registries. We also investigated the predictive or prognostic value of sex in patients who received specific treatments across all the study cohorts in which comprehensive data on clinical features, response to treatment,^16^ and outcome were available.

We aimed to study the relationships between sex and anaemia, the latter having a well-known prognostic effect in patients with myelodysplastic syndrome.^3^, ^4^, ^13^ We compared the probability of overall survival of men and women with the same haemoglobin value adjusted for age in the EuroMDS and IWG-PM cohorts.

We developed new personalised prognostic tools including sex information, and overall survival was selected as the primary endpoint for model development (appendix 1 pp 47–48). We incorporated sex into a prognostic model that included age and the established IPSS-R variables. We developed a sex-informed prognostic scoring system on the GEMSD (learning cohort) and Dusseldorf MDS (validation cohort) registries.

Furthermore, we included genomic features in the prognostic model (ie, the presence or absence of chromosomal abnormalities and the mutational status of 44 genes). To do this, we developed a sex-informed genomic scoring system on the EuroMDS (learning cohort) and IWG-PM (validation cohort) populations.

To compare the performance of all newly generated models with respect to IPSS-R, we did an analysis of the EuroMDS and IWG-PM cohorts, which allowed us to calculate all predictions in each patient. Newly generated scores were built to derive a patient-specific probability of survival; to be comparable with IPSS-R, a five-category risk schema was defined for both systems.^15^ To assist clinicians in becoming familiar with new prognostic tools, we have created a web portal that allows personalised outcome predictions to be generated based on a sex-informed approach.

### Outcomes

The key endpoint was overall survival defined as the time between diagnosis and death (from any cause) or last follow-up (for censored observations). The secondary endpoint was leukaemia-free survival, defined as the time between diagnosis and acute myeloid leukaemia evolution (if any) or last follow-up (for censored observations). When focusing on patient populations who received a specific treatment, overall survival was calculated as the time between the start of treatment and death or final follow-up. The response to treatment was assessed according to 2006 International Working Group response criteria.^3^ Prevalence of comorbidity, mortality cause, and the effect of anaemia on clinical outcomes were also analysed.

### Statistical analysis

Numerical variables are summarised by median and IQR; categorical variables are described with count and relative frequency of subjects in each category. When estimating the occurrence of leukaemic versus non-leukaemic death by competing risk analysis, deaths occurring after leukaemic evolution were considered to be related to leukamia and deaths from all causes except leukemic evolution were considered to be unrelated to leukaemia. Survival curves were estimated with the Kaplan-Meier method and compared by log-rank test. Multivariable survival analyses were done with Cox's proportional hazards regression models (survival R package version 3.3.1). p<0·05 was considered to indicate a significant difference. p values were adjusted for multiple testing using the Benjamini-Hochberg procedure. The 95% CI was computed using the R_summary_function applied to the fitted survival analysis or Cox's models.

Bradley-Terry models were used to estimate the timing of mutation acquisition.^14^, ^17^, ^18^ Bayesian networks analysis was used to infer the structure of conditional dependencies among mutations, that is, how the presence of a given mutation influences the probability of the others (causality). Bayesian networks were obtained using the GOBNILP software (version 1.6.3).^14^, ^17^, ^18^ Hierarchical Dirichlet processes were applied to define clusters capturing broad dependencies among all gene mutations and cytogenetic abnormalities.^14^, ^17^, ^18^ To this aim, data were modelled using the R package HDP, available online.

Random effects Cox multistate models were used to incorporate sex into novel personalised prognostic systems. The discriminatory power of the models and the relative goodness of fit for the predictive score were evaluated using the Harrell's concordance index (C-index).^14^, ^18^ These functions were implemented in the R package CoxHD, version 0.0.61, available online.

The new sex-based web scores were built as a weighted sum of prognostic variables observed for each case to derive a patient-specific risk score. To assess the fraction of patients who were assigned to a different prognosis with respect to the conventional IPSS-R (which is based on five risk categories), the new scores were scaled so that a score of 0 represented the average patient (ie, a hypothetical patient with mean values for all variables), whereas values of –1, 1, or 2 corresponded to half, double, or a four-times risk compared with the average patient, respectively. Accordingly, a five-category risk schema was defined for all systems, thus allowing a direct comparison.^15^

Detailed descriptions of the statistical methods for the mutation acquisition order, the identification of co-mutational patterns and mutually exclusive mutations in patients with myelodysplastic syndromes stratified by sex, the Bayesian networks to define relationships between genomic abnormalities in myelodysplastic syndromes stratified by sex, the Dirichelet Process Clustering to identify myelodysplastic syndrome molecular subtypes, and details on the use of Cox proportional hazard assumptions to assess personalised prognostic risk based on demographics, clinical features, and genomic features, are reported in appendix 1 (pp 24–25, 29, 37, 47). This trial is registered with ClinicalTrials.gov, NCT04889729.

### Role of the funding source

The funders of the study had no role in study design, data collection, data analysis, data interpretation, or writing of the report.

## Results

We enrolled 13 284 patients from different cohorts (figure 1). The study included 7792 (58·7%) men and 5492 (41·3%) women. 10 906 (82·1%) patients were White, and race was not reported for 2378 (17·9%) patients. Demographics and clinical characteristics of the study participants are summarised in table 1. 3393 (31·7%) of 10 709 evaluable patients received red blood cell transfusions, 1158 (10·8%) patients were treated with erythroid stimulating agents, 2122 (19·8%) patients were treated with hypomethylating agents, 714 (6·7%) patients were treated with acute myeloid leukaemia-like chemotherapy, and 1176 (11·0%) patients received allogeneic haematopoietic stem-cell transplantation. We found no significant difference in the prevalence of different treatment strategies between men and women (data not shown).

**Table 1:** Demographic, haematological, and clinical features of study participants collected at the time of diagnosis

		**Total**	**Men**	**Women**	**p value**
Patients		13 284	7792 (58·7%)	5492 (41·3%)	<0·0001
Age, years		73 (18–101)	73 (18–101)	73 (18–99)	0·84
Race*					
	White	10 906 (82·1%)	6350 (81·5%)	4547 (82·8%)	0·56
	Not reported	2378 (17·9%)	1442 (18·5%)	945 (17·2%)	0·62
Haemoglobin, g/dL		9·8 (2·6–19·6)	9·9 (2·6–19·6)	9·7 (2·7–16·6)	<0·0001
Red blood cell transfusion dependency		3545/12 488 (28·4%)	2070/7348 (28·2%)	1475/5140 (28·7%)	0·52
Neutrophils, ×10^9^/L		1·91 (0–55·23)	1·85 (0–41·8)	2 (0–55·23)	<0·0001
Platelets, ×10^9^/L		138 (1–1491)	123 (1–1383)	163 (2–1491)	<0·0001
WHO 2016 MDS category					
	MDS with isolated del(5q)	729 (5·5%)	192 (2·5%)	537 (9·8%)	<0·0001
	MDS-SLD	1370 (10·3%)	755 (9·7%)	615 (11·2%)	0·0049
	MDS-RS-SLD	1422 (10·7%)	812 (10·4%)	610 (11·1%)	0·21
	MDS-MLD	3831 (28·8%)	2418 (31·0%)	1413 (25·7%)	<0·0001
	MDS-RS-MLD	1382 (10·4%)	823 (10·6%)	559 (10·2%)	0·48
	MDS-EB1	2234 (16·8%)	1363 (17·5%)	871 (15·9%)	0·013
	MDS-EB2	2239 (16·9%)	1384 (17·8%)	855 (15·6%)	0·0009
	MDS-U	77 (0·6%)	45 (0·6%)	32 (0·6%)	0·97
IPSS-R cytogenetic risk group					
	Very good	476/11 495 (4·1%)	435/6812 (6·4%)	41/4683 (0·9%)	<0·0001
	Good	8358/11 495 (72·7%)	4770/6812 (70·0%)	3588/4683 (76·6%)	<0·0001
	Intermediate	1217/11 495 (10·6%)	772/6812 (11·3%)	445/4683 (9·5%)	0·0017
	Poor	544/11 495 (4·7%)	315/6812 (4·6%)	229/4683 (4·9%)	0·51
	Very poor	900/11 495 (7·8%)	520/6812 (7·6%)	380/4683 (8·1%)	0·35
IPSS-R risk group					
	Very low	1355/11 091 (12·2%)	824/6575 (12·5%)	531/4516 (11·8%)	0·22
	Low	2066/11 091 (18·6%)	1271/6575 (19·3%)	795/4516 (17·6%)	0·022
	Intermediate	4241/11 091 (38·2%)	2389/6575 (36·3%)	1852/4516 (41·0%)	<0·0001
	High	1137/11 091 (10·3%)	688/6575 (10·5%)	449/4516 (9·9%)	0·37
	Very high	2292/11 091 (20·7%)	1403/6575 (21·3%)	889/4516 (19·7%)	0·035

Data are n, n (%), median (IQR), or n/N (%). IPSS-R=Revised International Prognostic Scoring System. MDS=myelodysplastic syndrome. MDS-SLD=MDS with single lineage dysplasia. MDS-RS-SLD=MDS with ring sideroblasts and single lineage dysplasia. MDS-MLD=MDS with multilineage dysplasia. MDS-RS-MLD=MDS with ring sideroblasts and multilineage dysplasia. MDS-EB1=MDS with excess of blasts, type 1. MDS-EB1=MDS with excess of blasts, type 2. MDS-U=unclassified MDS.

* Self-reported.

**Figure 2:**
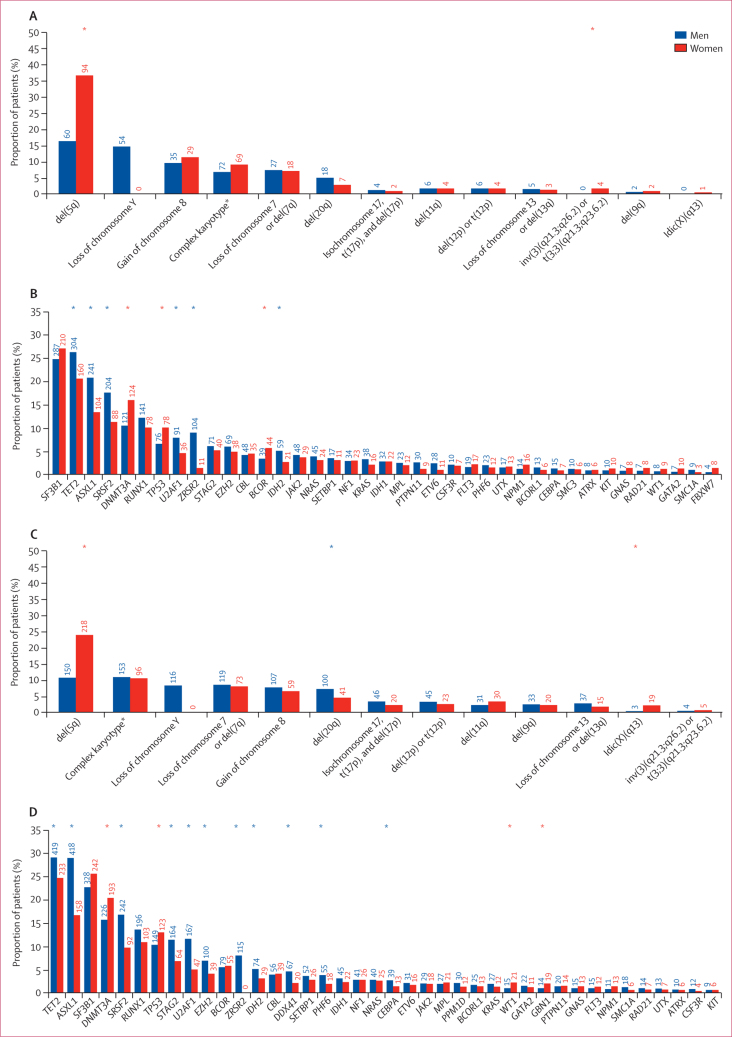
Sex-bias in chromosomal abnormalities and gene mutations in 2025 patients from the EuroMDS cohort (A and B) and in 2387 patients from the IWG-PM cohort (C and D) Numbers above the bars indicate the patient counts. Blue asterisks indicate a significant increase in prevalence in men. Red asterisks indicate a significant increase in prevalence in women. Idic(X)=isodicentric X chromosome. IWG-PM=International Working Group for Prognosis in MDS. *Three or more abnormalities.

Regarding sex-spectrum diversity in patients with myelodysplastic syndrome according to genomic features, detailed results are available in appendix 1 (pp 13–24). We analysed the sex bias of chromosomal abnormalities and found that del(5q) predominantly occurred in women (p<0·0001). The prevalence of patients with at least one mutation was 82·8% (998 of 1205 patients) and 92·6% (1335 of 1442 patients) in men versus 76·2% (625 of 820 patients) and 84·9% (802 of 945 patients) in women in the EuroMDS and IWG-PM cohorts, respectively (p<0·0001), with a higher number of mutations per patient in men versus women (p=0·015 and p<0·0001). We observed that the following seven genes were significantly more mutated in men than in women: *ASXL1* (p<0·0001 in both EuroMDS and IWG-PM)*, DDX41* (p=0·0062 in IWG-PM; p value not available in EuroMDS)*, IDH2* (p<0·0001 in EuroMDS and p=0·042 in IWG-PM)*, SRSF2* (p<0·0001 in both EuroMDS and IWG-PM)*, TET2* (p=0·031 in EuroMDS and p=0·035 in IWG-PM)*, U2AF1* (p=0·033 in EuroMDS and p<0·0001 in IWG-PM), and *ZRSR2* (p<0·0001 in both the EuroMDS and IWG-PM cohorts). By contrast, mutations in *DNMT3A* (p<0·0001 in EuroMDS and p=0·011 in IWG-PM) and *TP53* (p=0·030 in EuroMDS and p=0·037 in IWG-PM) were enriched in women; figure 2; appendix 1 pp 13–15). We observed a higher number of mutations in men for all mutation types, including point mutations and indels (p<0·0001), but the distribution of mutation hotspots and variant allele frequencies were not significantly different in men versus women (data not shown). Considering gene pathways,^14^ splicing factor mutations and mutations related to DNA methylation and chromatin and histone modifier were more prevalent in men (splicing-related genes p<0·0001 in both EuroMDS and IWG-PM; DNA methylation p=0·046 in EuroMDS and p<0·0001 in IWG-PM), whereas tumour suppressor gene mutations were more frequently found in women (p=0·0073 in EuroMDS and p<0·0001 in IWG-PM; appendix 1 pp 14–15).

We identified three different patterns of sex distribution and their relation to age, including: genes with preponderance in men across all age groups—*ASXL1, DDX41, IDH2, SRSF2, TET2, U2AF1*, and *ZRSR2*; genes with a preponderance in women across all age groups—*DNMT3A* and *TP53*; and mutations without a sex bias, regardless of age (including the all the remaining genes analysed; appendix 1 pp 16–23). Overall, we identified that men were more likely to have mutations that occurred more often in early disease phases and less frequently had mutations representing late events associated with disease evolution, compared with women (appendix 1 p 24).

We extracted mutational signatures. Pairwise associations among genes and cytogenetic abnormalities revealed a complex landscape of positive and negative associations, which was significantly different between men and women (appendix 1 pp 25–28). Bayesian networks and Dirichlet processes were applied to infer the structure of conditional dependencies among all genomic abnormalities (appendix 1 pp 29–38). According to these analyses, in both EuroMDS and IWG-PM cohorts, mutations in splicing related genes were mutually exclusive, irrespective of sex (p<0·0001). *SRFS2*-related myelodysplastic syndromes were more common in men than in women (p=0·0024 in EuroMDS and p<0·0001 in IWG-PM). Considering specific *SRSF2* mutational patterns, *TET2* co-mutations were predominated in women (p=0·018 in EuroMDS and p<0·0001 in IWG-PM), whereas co-mutational pattern, including *RUNX1*, were predominant in men (p<0·0001 in both cohorts). In patients with *U2AF1* mutations, co-mutations involving the *ASXL1* gene were observed more frequently in men than in women (p=0·0082 in EuroMDS and p=0·011 in IWG-PM). Myelodysplastic syndromes associated with *TP53* mutations or complex karyotypes (p=0·010 in EuroMDS and p=0·013 in IWG-PM), myelodysplastic syndrome with acute myeloid leukaemia-like mutations (p=0·024 in EuroMDS and p=0·043 in IWG-PM), and myelodysplastic syndromes without specific genomic features (p=0·0012 in EuroMDS and p=0·0014 in IWG-PM) were more common in women than in men. Co-mutational patterns in myelodysplastic syndromes with *TP53* mutations or complex karyotypes were highly conserved across sex (appendix 1 pp 29–38). Regression analysis showed that man-specific genomic profiles were associated with a higher degree of morphological dysplasia (p=0·019 in EuroMDS and p=0·013 in IWG-PM) and more severe cytopenias (neutropenia and thrombocytopenia, p<0·0001 in both cohorts) compared with woman-specific genotypes.

Our findings described substantial sex diversity in myelodysplastic syndromes in terms of disease genotype and distinct phenotypic features, which was confirmed across different patient populations. On the basis of these results, we analysed the effect of sex on the clinical outcomes of myelodysplastic syndromes. In the EuroMDS cohort, we observed that sex had a significant effect on clinical outcome, with men showing worse overall survival (median overall survival 81·3 months, 95% CI 70·4–95·0 in men, and 123·5 months, 104·5–127·5 in women; hazard ratio [HR] 1·40, 95% CI 1·26–1·52; p<0·0001), whereas no significant effect was noticed regarding leukaemia-free survival (median leukaemia-free survival 158·8 months, 95% CI 145·2–182·5) in men, and 146·9 months, 133·3–160·5 in women; HR 0·87, 95% CI 0·65–1·15; p=0·34). The prognostic impact of sex was evident in early disease stages (defined by IPSS-R score ≤3·5; HR 1·62, 95% CI 1·23–2·08; p<0·0001) but was not apparent in patients with advanced disease (IPSS-R >3·5; 1·24, 0·93–1·65; p=0·15; appendix 1 p 41). The independent prognostic effect of sex was maintained in a multivariate model that included age, haemoglobin level, neutrophil and platelet count, proportion of bone marrow blasts, and karyotype as covariates (HR 1·24, 95% CI 1·11–1·46; p=0·0010).

To better understand factors contributing to the survival difference between men and women, we did a competing risk analysis patients with early disease stages, considering leukaemic death versus non-leukaemic death as endpoints. The 5-year risk of non-leukaemic death was 32·1% in men versus 18·4% in women (p<0·0001), but no difference was found regarding the risk of leukemic death (appendix 1 p 41).

Comorbidity has an unfavourable effect on the life expectancy of patients with myelodysplastic syndrome.^11^, ^12^ In the EuroMDS cohort, the prevalence of comorbidity at diagnosis was significantly different between men and women (p=0·0031 in an analysis adjusted for age). Cardiac and renal comorbidities were more common in men than in women (281 [23·3%] of 1205 men *vs* 75 [9·1%] of 820 women; p<0·0001), whereas no significant differences across sex were noted for hepatic, pulmonary, and neoplastic diseases. A high prevalence of cardiac comorbidity was observed in patients with myelodysplastic syndromes with loss of chromosome Y (22 [41%] of 54 patients), and no significant effect of mutations in genes related to clonal haematopoiesis (*ASXL1, DNMT3A*, and *TET2*) was noted on the prevalence of cardiac disease (p=0·48). We then explored specific causes of non-leukaemic death in patients with myelodysplastic syndromes. The leading cause of non-leukaemic death was cardiac death, and the risk of cardiac death was higher in men than in women (HR 1·76, 95% CI 1·29–2·45; p<0·0001, after adjustment for age).

We observed a reduced likelihood of overall survival in men across all haemoglobin levels in both populations (p<0·0001; appendix 1 pp 42–45). We explored the prognostic effect of different haemoglobin values in men and women. In both cohorts, anaemia had a negative prognostic impact at haemoglobin concentrations below 11 g/dL in men (HR 2·48, 95% CI 1·49–4·10; p<0·0001 in EuroMDS and 1·97, 1·17–3·30; p=0·012 in IWG-PM) and below 10 g/dL in women (2·96, 1·61–5·44; p<0·0001 in EuroMDS and 1·72, 1·03–2·86, p=0·039 in IWG-PM). This effect was maintained in multivariable analysis (appendix 1 p 46). Focusing on the EuroMDS cohort, men with haemoglobin concentrations less than 11 g/dL and women with less than 10 g/dL had an increased probability of cardiac death (HR 1·31, 95% CI 1·09–1·58; p=0·0044).

To provide evidence for generalisability of these findings, we did an independent validation on the GEMSD and Dusseldorf MDS registries. The probability of overall survival was lower in men compared with women (median overall survival in the GEMSD MDS registry was 54·7 months, 95% CI 52·4–59·1 in men and 74·4 months, 69·3–81·2 in women; HR 1·30, 95% CI 1·23–1·35; p<0·0001; median overall survival in the Dusseldorf MDS registry was 40·0 months, 95% CI 33·4–43·7 in men and 54·2 months, 38·6–63·8 in women; HR 1·23, 95% CI 1·08–1·36; p<0·0001; appendix 1 pp 39–40). To further define the prognostic effect of sex according to disease stage, we analysed patients stratified by IPSS-R risk groups. We observed a significant sex-related survival effect in patients classified into very low and low-risk groups (table 2). No significant effect of sex on leukaemia-free survival was detectable across all risk categories (data not shown).

**Table 2:** Overall survival of patients with myelodysplastic syndromes, classified into revised International Prognostic Scoring System risk categories and stratified by sex

	**Men**	**Women**	**p value**
**EuroMDS cohort**			
Very low	Not reached	Not reached	<0·0001
Low	95·0 (75·1–191·3)	127·5 (104·5–127·5)	<0·0001
Intermediate	42·6 (34·2–53·7)	112·5 (72·7–112·5)	<0·0001
High	34·0 (28·3–39·0)	49·9 (24·0–62·1)	0·084
Very high	14·6 (8·9–83·2)	16·1 (11·7–47·6)	0·92
**International Working Group for Prognosis in MDS cohort**			
Very low	215·3 (153·3–196·1)	270·9 (202·4–478·2)	<0·0001
Low	139·2 (123·9–151·1)	192·4 (171·2–228·7)	<0·0001
Intermediate	67·2 (60·0–90·4)	107·0 (76·8–164·9)	0·049
High	40·1 (31·5–58·3)	50·6 (38·9–74·1)	0·66
Very high	18·7 (14·4–29·4)	23·6 (15·4–35·8)	0·20
**Registry of Spanish MDS Group cohort**			
Very low	97·1 (86·4–323·1)	137·8 (122·3–148·7)	<0·0001
Low	60·4 (54·8–226·8)	89·1 (76·2–100·7)	<0·0001
Intermediate	34·2 (30·7–198·7)	40·9 (32·6–48·6)	0·24
High	16·3 (13·5–162·3)	18·5 (14·0–241·1)	0·19
Very high	6·6 (5·3–100·5)	8·6 (7·1–10·4)	0·076
**Düsseldorf MDS registry cohort**			
Very low	59·0 (42·0–108·0)	119·0 (63·0–403·0)	0·012
Low	59·0 (49·0–67·0)	91·0 (66·0–132·0)	<0·0001
Intermediate	37·0 (25·0–50·0)	29·0 (23·0–38·0)	0·085
High	22·0 (11·0–89·0)	16·0 (11·0–22·0)	0·40
Very high	10·0 (7·0–12·0)	13·0 (7·0–23·0)	0·20

Data are median overall survival (in months; 95% CI), unless otherwise indicated. The results of these analyses were confirmed after adjusting for age.

Regarding the predictive or prognostic value of sex in relation to treatments received, first, we studied 330 patients treated with erythropoiesis-stimulating agents. A treatment response was observed in 122 (37·0%) of 330 patients, with no significant difference between men and women (p=0·35). A significant effect of sex was noted on the probability of survival after treatment, with men showing worse outcomes (median overall survival since erythropoiesis-stimulating agents treatment was 72·3 months, 95% CI 60·1–102·5 in men and 95·9 months, 76·5–124·3) in women; HR 1·64, 95% CI 1·21–1·92; p=0·013).

We investigated the effect of sex in patients with myelodysplastic syndrome with bone marrow blasts of 10% of more, who were ineligible for transplantation and received hypomethylating agents. A response after four to six cycles of treatment was observed in 172 (46·1%) of 373 patients, with no significant difference between men and women (p=0·30). No significant prognostic effect of sex was noted on the probability of overall survival (median overall survival since hypomethylating agent treatment was 14·2 months, 95% CI 7·9–20·1 in men and 15·6 months, 9·0–24·6 in women; HR 1·12, 95% CI 0·86–1·46; p=0·41).

We focused on 702 patients with myelodysplastic syndrome who were treated with allogeneic haematopoieitc stem-cell transplantation. No significant effect of sex was noted on the probability of post-transplantation survival (median overall survival 29·7 months, 95% CI 19·1–41·6 in men and 34·9 months, 25·2–44·9 in women; HR 0·98, 95% CI 0·80–1·19; p=0·86).

**Figure 3:**
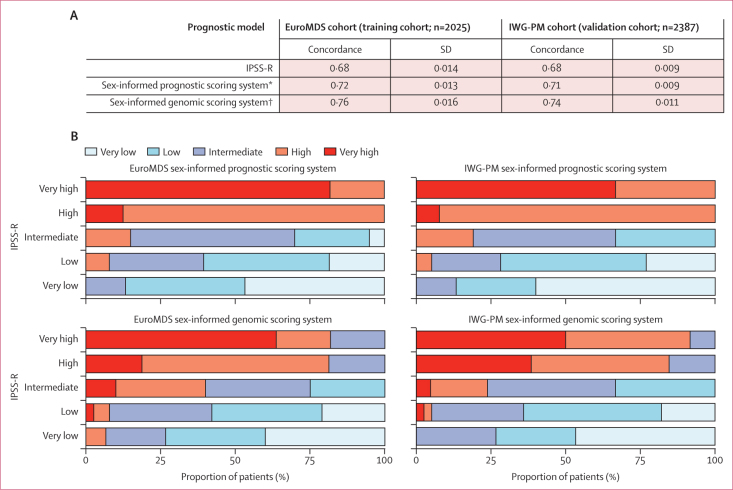
Concordance between IPSS-R, the sex-informed prognostic scoring system, and the sex-informed genomic scoring system (A) and restratification of IPSS-R to the sex-informed prognostic scoring system and sex-informed genomic scoring system groups in patients from EuroMDS and IWG-PM cohorts (B) IPSS-R=revised International Prognostic Scoring System. IWG-PM=International Working Group for Prognosis in MDS. *The sex-informed prognostic scoring system was based on sex, age, haemoglobin concentration, absolute neutrophil count, platelet count, proportion of bone marrow blasts, and cytogenetics (stratified according to IPSS-R criteria). †The sex-informed genomic scoring system was based on sex, age, haemoglobin concentration, absolute neutrophil count, platelet count, proportion of bone marrow blasts, cytogenetics (ie, presence or absence of single chromosomal abnormalities) and mutational status of 44 myelodysplastic syndrome-related genes (ie, presence or absence).

Finally, we developed new personalised prognostic tools including sex information with overall survival as the primary endpoint. First, we incorporated sex into a prognostic model that included age and the well-established IPSS-R variables. We developed a sex-informed prognostic scoring system on the GEMSD (learning cohort) snd Dusseldorf MDS (validation cohort) registries. We found that sex had strong independent prognostic power (HR 1·40, 95% CI 1·26–1·52; p<0·0001). Furthermore, we included genomic features in the prognostic model (ie, the presence or absence of chromosomal abnormalities and the mutation status of 44 genes). To this aim, we developed a sex-informed genomic scoring system on the EuroMDS (learning cohort) and IWG-PM (validation cohort) cohorts. Sex maintained a strong independent prognostic effect (HR 1·37, 95% CI 1·22–1·48; p<0·0001). To compare the performance of all newly generated models with respect to IPSS-R, we did an analysis on the EuroMDS and IWG-PM cohorts, which allowed us to calculate all predictions in each patient. In both cohorts, the best performance was seen using the model that integrated sex and genomic information (figure 3).

A five-to-five mapping between the IPSS-R and new score categories resulted in the re-stratification of 871 (43·0%) of 2025 patients from the EuroMDS cohort and 1003 (42·0%) of 2387 patients from the IWG-PM cohort by using the sex-informed prognostic scoring system, and of 1134 (56·0%) of 2025 patients from the Euro MDS cohort and 1265 (53·0%) of 2387 patients from the IWG-PM cohort by using the sex-informed genomic scoring system (figure 3). We created a web portal that enables outcome predictions based on a sex-informed personalised approach.

We determined the fraction of explained variation for clinical outcome that was attributable to different prognostic factors in predicting the probability of non-leukaemic death versus leukaemic death by the sex-informed genomic scoring system. In merged EuroMDS and IWG-PM cohorts, demographic features (age and sex) together with anaemia had a high predictive prognostic power of non-leukaemic death, whereas the effect of sex on predicting the probability of leukemic death was negligible. Genomic features and bone marrow blast had strong independent predictive power for leukaemic death (appendix 1 p 48).

## Discussion

In this study, we provide evidence for sex-dependent diversity in myelodysplastic syndromes in terms of disease genotype, phenotype, and clinical outcome. Sex biases were observed at the single-gene level, in co-mutational patterns of founding genomic lesions and in specific mutational pathways. Sex affected the probability of overall survival, with men showing a poorer prognosis. Men had a higher prevalence of cardiac comorbidity and risk of cardiac death compared with women, thus reducing their probability of survival. Anaemia had an adverse prognostic effect at different haemoglobin thresholds in men compared with women.

The incidence of myelodysplastic syndromes is more common in men for reasons that remain to be elucidated.^1^, ^2^, ^3^, ^10^, ^11^ In our study, this increased prevalence in men originated at an early stage of the disease, suggesting a possible correlation with age-related clonal haematopoiesis.^19^, ^20^ Accordingly, we observed a higher prevalence of mutations related to DNA methylation (accounting for most of the events related to clonal haematopoiesis) in men compared with women.

We created a web portal (see Methods section for further details) that enables outcome predictions based on a sex-informed personalised approach. This website allows a direct comparison between conventional prognostic assessment provided by IPSS-R versus the individual probability of overall survival and leukaemia-free survival as generated by the sex-informed prognostic scoring system and sex-informed genomic scoring system.

The sex-dependent diversity of the genomic landscape of myelodysplastic syndromes might also be affected by sex-related differences in the mechanisms of mutation acquisition or selection of mutated clones. Sex bias was observed in some genes located on the X chromosome, such as *ZRSR2*. In men, an oncogenic allele on the X chromosome will always be expressed from that single copy, whereas in women a mutated allele on the inactive X chromosome will have no harmful effect unless that gene escapes X inactivation.^21^, ^22^

Several factors have been reported to contribute to differences in autosomal gene mutations between men and women in cancer, including increased defects in DNA damage response pathways in men, the effect of hormonal receptors on various cellular functions, and sex differences in the immune system.^22^, ^23^, ^24^ These mechanisms might also play a part in explaining sex-bias in myelodysplastic syndrome, but further investigation is necessary. We observed an increased mutational burden in men and a sex bias was present in mutation frequency and co-mutational pathways of several founding events (splicing factor genes). *TP53* mutations were more prevalent in women and showed a stronger association with del(5q). Oestrogenic hormones significantly affect the levels or function of components of the p53 network, thus contributing to a higher prevalence of *TP53* dysfunction in women with different types of cancers.^24^, ^25^ We found that myelodysplastic syndromes with germline *DDX41* mutations^2^ had a strong preponderance in men. In this context, sex was found to influence penetrance in a variety of ways, including allelic variation, gender specific genomic architecture, and genomic imprinting.^26^ Overall, these findings support the hypothesis that disease heterogeneity in myelodysplastic syndromes in terms of genotype can be driven by sex-related factors.

Life expectancy in the general population differs between men and women, and this appears to also be the case in patients with myelodysplastic syndromes.^10^, ^11^, ^12^ Nevertheless, sex is not included in outcome prediction provided by currently available prognostic scores and is managed as a confounding factor.^4^, ^15^ We provide evidence that sex has an independent prognostic effect in myelodysplastic syndromes as a result of the following contributing effects: an impact on the natural course of disease by way of affecting phenotypic features, an increased risk of cardiovascular complications and cardiac death in men, and a differential prognostic effect of the severity of anaemia. Focusing on comorbidity, we made a preliminary observation of a high prevalence of cardiac comorbidity in patients with Y chromosome loss.^27^

Clinical management of patients with low-risk myelodysplastic syndromes, in which the leading cause of death is cardiovascular disease, would benefit from the use of prognostic tools that focus on detrimental interactions between the major disease phenotype (ie, anaemia) and patient-related factors, such as age, sex, and comorbidity, rather than putting the emphasis on the risk of leukaemic evolution.^11^, ^12^, ^13^ In this study, we provide evidence that a sex-informed approach could significantly improve personalised decision making in patients with myelodysplastic syndromes with respect to conventional IPSS-R, resulting in risk re-stratification of a substantial proportion of patients, and this effect was maintained even when mutation screening was included. In our combined clinical and molecular model, sex had a predictive prognostic power of non-leukaemic death, whereas genomic features and bone marrow blasts had strong predictive power for leukaemic death. Therefore, we propose that sex should be integrated into the IPSS-R and into novel prognostic tools based on combined clinical and genomic features.^14^, ^15^

In patients receiving treatment for myelodysplastic syndromes, no significant predictive or prognostic effect of sex was noticed on hypomethylating agents and transplantation. Low-risk patients with symptomatic anaemia treated with erythropoiesis-stimulating agents had a better response rate and duration than did those treated after the onset of transfusions.^3^, ^25^ Our results suggest that once mild anaemia occurs (defined by objective sex-specific thresholds), optimal management is needed to limit its negative effect on clinical outcomes. Our findings could have implications for the design of clinical trials.^28^ Inclusion of a sex-informed approach is expected to improve the selection of patients for participation in clinical trials with erythropoiesis-stimulating agents. Moreover, the sex-dependent effect of anaemia, together with differences in the probability of survival between men and women, strengthen the rationale to report study results according to sex.^29^, ^30^

Our study has some limitations. First, retrospective studies might be not representative of the general population. However, we were able to collect data from a large population of patients with myelodysplastic syndromes and the analyses were validated across different cohorts, thus limiting the possibility of selection bias and improving the generalisability of the results. Moreover, genomic screening was based on a small number of genes, thus potentially affecting the capability of analyses to capture all sex biases in single genes, gene pathways, and co-mutational patterns. However, our analysis included all relevant myelodysplastic syndrome-related genes, thus providing a comprehensive characterisation of molecular landscape of these diseases.

In conclusion, our results suggest that a sex-informed approach can improve the personalised decision making process in patients with myelodysplastic syndromes and should be considered in the design of clinical trials including low-risk patients.

## Data sharing

With the aim of helping clinicians be familiar with the proposed sex-informed prognostic tools, we provide public access to a web portal (further details in the methods) that allows outcome predictions to be generated based on demographic, clinical, and genomic features. Requests for access to data from the study should be addressed to the GenoMed4All scientific committee (please contact MGDP at matteo.della_porta@hunimed.eu). All proposals requesting data access must specify how the data will be used, and all proposals will need the approval of the GenoMed4All scientific committee before data release.

## Declaration of interests

AAP reports payment or honoraria for lectures, presentations, speaker's bureaus, manuscript writing, or educational events from Bristol Myers Squibb, Jazz, and Janssen; and participation on a data safety monitoring board or advisory board for Bristol Myers Squibb. ASM reports consulting fees from Bristol Myers Squibb-Celgene, Novartis, Astellas Pharma, Jazz, and Pfizer; and participation on a data safety monitoring board or advisory board for Bristol Myers Squibb-Celgene, Novartis, and Jazz. ARa reports consulting fees from Amgen, Omeros, Novartis, Astellas Pharma, Jazz Pharmaceuticals, Abbvie, Janssen, Pfizer, Incyte, and Kite-Gilead; and payment or honoraria for lectures, presentations, speaker's bureaus, manuscript writing, or educational events from Amgen, Omeros, Novartis, Astellas Pharma, Jazz Pharmaceuticals, Abbvie, Janssen, Pfizer, Incyte, and Kite-Gilead. AS reports consulting fees from Sanofi, Incyte, Takeda, Bristol Myers Squibb, Roche, Abbvie, Amgen, Celgene, Servier, Gilead, AstraZeneca, Pfizer, Lilly, Sandoz, Eisai, Novartis, Bayer, and MSD; and participation on a data safety monitoring board or advisory board for Bristol Myers Squibb, Servier, Gilead, Pfizer, Eisai, Bayer, and MSD. ViS reports consulting fees from Bristol Myers Squibb and Novartis; and participation on a data safety monitoring board or advisory board for Astex, Bristol Myers Squibb, Geron, Gilead, Menarini, and Novartis. UP reports consulting fees from Amgen, Janssen, Novartis, BerGenBio, and Celgene; payment or honoraria for lectures, presentations, speaker's bureaus, manuscript writing, or educational events from Celgene-Jazz; support for attending meetings or travel from Celgene; and participation on a data safety monitoring board or advisory board for Celgene-Jazz. MD-C reports participation on a data safety monitoring board or advisory board for Bristol Myers Squibb and Novartis. All other authors declare no competing interests.
