# Supplementary material for: A sex-informed approach to improve the personalised decision making process in myelodysplastic syndromes: a multicentre, observational cohort study
Source: Lancet Haematol. 2022 Nov 24;10(2):e117–28. doi: 10.1016/S2352-3026(22)00323-4 (PMC9886555; doi:10.1016/S2352-3026(22)00323-4)
Supplement: Supplementary appendix 1 [file mmc1.pdf]

THE LANCET
Haematology

Supplementary appendix 1

This appendix formed part of the original submission and has been peer reviewed. We post it as supplied by the authors.

Supplement to: GenoMed4All consortium. A sex-informed approach to improve the personalised decision making process in myelodysplastic syndromes: a multicentre, observational cohort study. *Lancet Haematol* 2022; published online Nov 24. https://doi.org/10.1016/S2352-3026(22)00323-4.

Supplementary Appendix

A sex-informed approach to improve personalized decision-making process in myelodysplastic syndromes

This appendix has been provided by the authors to give readers additional information about their work

Table of Contents

**Supplementary _File_1 (SF1) - Study populations**<br>• Description of study populations<br>• Probability of overall survival according to WHO 2016 subtype and IPSS-R risk category	**Page 2**
**Supplementary_File_2 (SF2) – Cytogenetics and mutation screening**	**Page 11**
**Supplementary_File_3 (SF3) – List of pathogenic variants**<br>• EuroMDS cohort (see attached excel file)<br>• IWG-PM cohort (available at https://github.com/papaemmelab/IPSSMstudy)	-
**Supplementary_File_4 (SF4)- Genomic landscape of MDS by sex**<br>• Prevalence of chromosomal abnormalities in patients from EuroMDS and IWG-PM cohorts stratified by sex<br>• Prevalence of gene mutations in patients from EuroMDS and IWG-PM cohorts stratified by sex<br>• Cumulative counts and densities per age in sex-biased genes in MDS patients from EuroMDS and IWG-PM cohorts<br>• Distribution of gene mutations in patients from EuroMDS and IWG-PM cohorts stratified sex across age categories	**Page 16**
**Supplementary_File_5 (SF5) – Time of mutation acquisition**<br>• Determination of MDS mutation order in EuroMDS cohort	**Page 24**
**Supplementary_File_6 (SF6) - Identification of genomic associations and subgroups in MDS by sex**<br>• Pairwise associations among genes and cytogenetic abnormalities in patients from EuroMDS and IWG-PM cohorts stratified by sex<br>• Genomic Landscape through Bayesian Networks in patients from EuroMDS EuroMDS and IWG-PM cohorts stratified by sex<br>• Dirichlet processes	**Page 25**
**Supplementary_File_7 (SF7) - Sex effect on MDS clinical outcome**<br>• Probability of overall survival of MDS patients belonging to retrospective EuroMDS and IWG-PM cohorts, and to prospective registry of Spanish MDS Group (GESMD) and Düsseldorf MDS registry, stratified by sex<br>• Competing risk analysis of leukemic death vs. non leukemic death in MDS from EuroMDS cohort stratified by sex<br>• Probability of survival of patients stratified by sex, according to different hemoglobin values (Euro MDS cohort, IWG-PM cohort, registry of Spanish MDS Group (GESMD) and Düsseldorf MDS registry)	**Page 39**
**Supplementary_File_8 (SF8)- Sex-informed prognostic assessment in myelodysplastic syndromes**<br>• Cox proportional-hazards model (coxph)<br>• Random effects Cox proportional-hazards multistate model (CoxHD)<br>• Goodness of fit and model comparison based on the concordance statistic	**Page 47**

SUPPLEMENTARY_FILE_1 (SF1) – Study populations

Study Cohort	Characteristics of MDS populations and sample size	Available data
**COHORT#1:**<br>**Patients from EuroMDS_network**	Retrospective cohort of 2,025 patients affected with MDS according to 2016 WHO classification	- comprehensive information on demographic, clinical and haematological features (collected at diagnosis), treatments and outcomes<br><br>- mutational screening on 47 MDS-related genes performed at diagnosis
**COHORT#2:**<br>**Patients from IWG-PM_network**	Retrospective cohort of 2,387 patients affected with MDS according to 2016 WHO classification	- comprehensive information on demographic, clinical and haematological features (collected at diagnosis), treatments and outcomes<br><br>- mutational screening on 44 MDS-related genes overlapping with EuroMDS cohort performed at diagnosis
**COHORT#3:**<br>**Patients from the registry of Spanish_MDS_Group (GESMD)**	Prospective cohort of 7,687 patients affected with MDS according to 2016 WHO classification	- comprehensive information on demographic, clinical and haematological features (collected at diagnosis), treatments and outcomes
**COHORT#4:**<br>**Patients from Düsseldorf_MDS_registry, Germany**	Prospective cohort of 1,185 patients affected with MDS according to 2016 WHO classification	- comprehensive information on demographic, clinical and haematological features (collected at diagnosis), treatments and outcomes

Supplementary Table_1A_SF1. *Demographic, haematological and clinical features of 2,025 patients from EuroMDS cohort, collected at the time of diagnosis and information on treatment.*

Variable	All patients	Men	Women	P value
**Patients (number)**	2,025	1,205 (59·5%)	820 (40·4%)	<0·0001
**Age (years)**	69 (18-94)	69 (19-92)	68 (18-94)	0·0921
**AGE categories**	1968	1180 (59·3%)	788 (39·7%)	-
<50	238 (12·1%)	128 (10·8%)	110 (14%)	0·0381
50-60	311 (15·8%)	169 (14·3%)	142 (18%)	0·0276
60-70	523 (26·6%)	343 (29·1%)	180 (22·8%)	0·0022
70-80	670 (34%)	403 (34·2%)	267 (33·9%)	0·9017
>80	226 (11·5%)	137 (11·6%)	89 (11·3%)	0·8296
**Haemoglobin (Hb, g/dL)**	9·8 (2·8-19·6)	9·9 (2·8-11·3)	9·7 (4·0-15·7)	0·0110
**Haemoglobin categories**	1,854	1,108 (59·8%)	746 (40·2%)	-
Normal Hb values	189 (10·2%)	108 (9·7%)	81 (10·9%)	0·4384
<Normal values -11 g/dl	341 (18·4%)	239 (21·6%)	102 (13·7%)	<0·0001
<11-10 g/dl	340 (18·3%)	189 (17·1%)	151 (20·2%)	0·0825
<10-9 g/dl	363 (19·6%)	202 (18·2%)	161 (21·6%)	0·0747
<9-8 g/dl	282 (15·2%)	190 (17·1%)	92 (12·3%)	0·0046
<8 g/dl	339 (18·6%)	180 (16·2%)	159 (21·3%)	0·0056
**RBC transfusion dependency (%)**	451/2,025 (22·3%)	265/1,205 (22·0%)	186/820 (22·7%)	0·7137
**Neutrophils (x10^9/L)**	1·92 (0·0-37·2)	1·86 (0·0-37·0)	2·0 (0·0-37·2)	0·0645
**Platelets (x10^9/L)**	129 (0-1,491)	116 (2-1,383)	144 (2-1,491)	<0·0001
**WHO category***	2,025	1,205 (59·5%)	820 (40·5%)	-
MDS with 5q-	75 (3·7%)	25 (2·1%)	50 (6·1%)	<0·0001
MDS-SLD	167 (8·2%)	85 (7·1%)	82 (10%)	0·0180
MDS-RS-SLD	213 (10·5%)	123 (10·2%)	90 (42·3%)	0·5803
MDS-MLD	455 (22·5%)	283 (23·5%)	172 (21%)	0·1842
MDS-RS-MLD	243 (12%)	160 (13·3%)	83 (10·1%)	0·0320
MDS-EB1	341 (16·8%)	206 (17·1%)	135 (16·5%)	0·7092
MDS-EB2	531 (26·2%)	323 (26·8%)	208 (25·4%)	0·4699
MDS-U	0	0	0	-
**IPSS- R cytogenetic risk group**	1,789	1,075 (59·8%)	723 (40·2%)	-
Very good	63 (3·5%)	60 (5·6%)	3 (0·4%)	<0·0001
Good	1317 (73·2%)	786 (71·4%)	549 (75·9%)	0·1805
Intermediate	210 (11·7%)	129 (12%)	81 (11·2%)	0·6061
Poor	107 (6%)	70 (6·5%)	37 (5·1%)	0·2206
Very poor	101 (5·6%)	48 (4·5%)	53 (7·3%)	0·0097
**IPSS- R risk group**	1,618	976 (60·3%)	642 (39·7%)	-
Very low	243 (15%)	141 (14·4%)	102 (15·9%)	0·4274
Low	606 (37·5%)	369 (37·8%)	237 (36·9%)	0·7171
Intermediate	323 (20%)	204 (20·9%)	119 (18·5%)	0·2443
High	259 (16%)	156 (16%)	103 (16%)	0·9743
Very high	187 (11·6%)	106 (10·9%)	81 (12·6%)	0·2799

Treatments*. 426 out of 1,904 patients (22·4%) received red blood cell transfusions; 304 patients (15%) were treated with erythroid stimulating agents; 316 patients (15·6%) were treated with hypomethylating agents; 300 patients (14·8%) were treated with AML-like chemotherapy; 492 patients (24·2%) received allogeneic stem cell transplantation; 131 patients (6·5%) were treated with other treatments (lenalidomide, immunosuppressive drugs). No significant difference was noticed in the prevalence of different treatment strategies between men and women (not shown)*

Supplementary Table_1B_SF1. *Demographic, haematological and clinical features of 2,387 patients from IWG-PM cohort, collected at the time of diagnosis and information on treatment.*

Variable	All patients	Men	Women	P value
**Patients (number)**	2,387	1,442 (60·4%)	945 (39·6%)	<0·0001
**Age (years)**	72 (19-98)	72 (19-95)	72 (19-98)	0·1934
**AGE categories**	2,386	1,441 (60·4%)	945 (39·6%)	-
<50	176 (7·4%)	90 (6·2%)	86 (9·1%)	0·0091
50-60	256 (10·7)	148 (10·3%)	108 (11·4%)	0·3710
60-70	603 (25·3%)	380 (26·4%)	223 (23·6%)	0·1275
70-80	863 (36·2%)	514 (35·7%)	349 (36·9%)	0·5306
>80	488 (20·5%)	309 (21·4%)	179 (18·9%)	0·1385
**Haemoglobin (Hb, g/dL)**	9·6 (4-16·6)	9·7 (4-16·6)	9·5 (4-14·8)	0·0410
**Haemoglobin categories**	2,359	1,426 (60·4%)	933 (41%)	-
Normal Hb values	215 (9·1%)	119 (8·3%)	96 (10·3%)	0·1087
<Normal values -11 g/dl	421 (17·8%)	314 (22%)	107 (11·5%)	<0·0001
<11-10 g/dl	386 (16·4%)	208 (14·6%)	178 (19·1%)	0·0039
<10-9 g/dl	508 (21·5%)	285 (20%)	223 (23·9%)	0·0237
<9-8 g/dl	463 (19·6%)	275 (19·3%)	188 (20·2%)	0·6049
<8 g/dl	366 (15·5%)	225 (15·8%)	141 (15·1%)	0·6623
**RBC transfusion dependency (%)**	519/2,049 (25·3%)	324/1,250 (25·9%)	195/799 (24·4%)	0·4421
**Neutrophils (x10^9/L)**	1·8 (0-10·2)	1·7 (0-10·2)	1·8 (0-9·9)	0·0921
**Platelets (x10^9/L)**	165 (2-1,055)	115 (2-956)	151 (5-1,055)	<0·0001
**WHO category***	2,387	1,442 (60·4%)	954 (39·6%)	-
MDS with 5q-	141 (5·9%)	34 (2·4%)	107 (11·3%)	<0·0001
MDS-SLD	255 (9·4%)	122 (8·5%)	103 (10·9%)	0·0550
MDS-RS-SLD	233 (9·8%)	140 (9·7%)	93 (9·8%)	0·9744
MDS-MLD	661 (27·7%)	425 (29·5%)	236 (25%)	0·0112
MDS-RS-MLD	202 (8·5%)	124 (8·6%)	78 (8·3%)	0·7153
MDS-EB1	439 (18·4%)	277 (19·2%)	162 (17·1%)	0·1676
MDS-EB2	416 (17·4%)	279 (19·3%)	137 (14·5%)	0·0016
MDS-U	70 (2·9%)	41 (2·8%)	29 (31·3%)	0·7798
**IPSS- R cytogenetic risk group**	2,323	1,406 (60·5%)	917 (39·5%)	-
Very good	91 (3·9%)	85 (6%)	6 (0·7%)	<0·0001
Good	1662 (69·8%)	938 (66·7%)	684 (74·6%)	0·0001
Intermediate	291 (12·5%)	190 (13·5%)	101 (11%)	0·0753
Poor	122 (5·3%)	67 (4·8%)	55 (6%)	0·1931
Very poor	197 (8·5%)	126 (9%)	71 (7·7%)	0·3027
**IPSS- R risk group**	2,265	1,372 (50·6%)	893 (39·4%)	-
Very low	356 (15·7%)	217 (15·8%)	139 (15·6%)	0·8727
Low	875 (38·6%)	494 (36%)	381 (42·7%)	0·0015
Intermediate	480 (21·2%)	300 (21·9%)	180 (20·2%)	0·3308
High	307 (13·6%)	201 (14·7%)	106 (11·9%)	0·0590
Very high	247 (10·9%)	160 (11·7%)	87 (9·7%)	0·1522

Treatments. *488 out of 2,359 evaluable patients (20·7%) received red blood cell transfusions; 459 patients (19·9%) were treated with hypomethylating agents; 45 patients (1·6%) were treated with AML-like chemotherapy; 232 patients (10%) received allogeneic stem cell transplantation; 161 patients (7%) were treated with other treatments (lenalidomide, immunosuppressive drugs). No significant difference was noticed in the prevalence of different treatment strategies between men and women (not shown)*

Supplementary Table_1C_SF1. *Demographic, haematological and clinical features of 7,687 patients from Spanish MDS Group registry (GESMD), collected at the time of diagnosis and information on treatment.*

Variable	All patients	Men	Women	P value
**Patients (number)**	7,687	4,420 (57·5%)	3,267 (42·5%)	<0·0001
**Age (years)**	75 (18-101)	75 (18-101)	76 (20-99)	0·2337
**AGE categories**	7,687	4,420 (57·5%)	3,267 (42·5%)	-
<50	344 (4·5%)	145 (3·3%)	199 (6·1%)	<0·0001
50-59	573 (7·5%)	315 (7·1%)	258 (7·9%)	0·2036
60-69	1,483 (19·3%)	902 (20·4%)	581 (17·8%)	0·0040
70-79	2,852 (37·1%)	1,726 (39·0%)	1,126 (34·5%)	<0·0001
>80	2,435 (31·7%)	1,332 (30·1%)	1,103 (33·8%)	0·0007
**Haemoglobin (Hb, g/dL)**	9·8 (2·6-17·7)	9·9 (2·6-17·7)	9·7 (2·7-16·6)	<0·0001
**Haemoglobin categories**	7,687	4,420 (57·2%)	3,267 (42·5%)	-
Normal Hb values	770 (10%)	415 (9·4%)	355 (10·9%)	0·0330
<Normal values -11 g/dl	1,446 (18·8%)	1,026 (23·2%)	420 (12·9%)	<0·0001
<11-10 g/dl	1,430 (18·6%)	747 (16·9%)	683 (20·9%)	<0·0001
<10-9 g/dl	1,311 (17·1%)	704 (15·9%)	607 (18·6%)	0·0022
<9-8 g/dl	1,641 (21·3%)	905 (20·5%)	736 (22·5%)	0·0299
<8 g/dl	1,089 (14·2%)	623 (14·1%)	466 (14·3%)	0·8338
**RBC transfusion dependency (%)**	2,142/7,304 (29·3%)	1,218/4,211 (28·9%)	924/3,093 (29·9%)	0·3784
**Neutrophils (x10^9/L)**	1·99 (0-55·23)	1·93 (0-41·8)	2·07 (0·02-55·23)	<0·0001
**Platelets (x10^9/L)**	147 (1-1,418)	130 (1-1,376)	176 (3-1,418)	<0·0001
**WHO category***	7,687	4,420 (57·5%)	3,267 (42·5%)	-
MDS with 5q-	415 (5·4%)	102 (2·3%)	313 (9·6%)	<0·0001
MDS-SLD	914 (11·9%)	509 (11·5%)	405 (12·4%)	0·2382
MDS-RS-SLD	928 (12·1%)	522 (11·8%)	406 (12·4%)	0·4115
MDS-MLD	2,252 (29·3%)	1,400 (31·7%)	852 (26·1%)	<0·0001
MDS-RS-MLD	868 (11·3%)	500 (11·8%)	368 (11·3%)	0·9475
MDS-EB1	1,257 (16·4%)	757 (17·1%)	500 (15·3%)	0·0327
MDS-EB2	1,046 (13·6%)	626 (14·2%)	420 (12·9%)	0·0985
MDS-U	7 (0·1%)	4 (0·1%)	3 (0·1%)	0·9848
**IPSSR cytogenetic risk group**	6,298	3,670 (58·3%)	2,628 (41·7%)	-
Very good	297 (4·7%)	248 (6·8%)	31 (1·2%)	<0·0001
Good	4,665 (70·9%)	2,617 (71·3%)	2,048 (77·9%)	<0·0001
Intermediate	655 (10·4%)	413 (11·3%)	242 (9·2%)	0·0088
Poor	269 (4·3%)	153 (4·2%)	116 (4·4%)	0·6353
Very poor	430 (6·8%)	239 (6·5%)	191 (7·3%)	0·2411
**IPSSR risk group**	6,298	3,670 (58·3%)	2,628 (41·7%)	-
Very low	1,563 (24·8%)	958 (26·1%)	605 (23%)	0·0052
Low	2,428 (38·6%)	1,329 (36·2%)	1,099 (41·8%)	<0·0001
Intermediate	1,069 (17%)	645 (17·6%)	424 (16·1%)	0·1331
High	672 (10·7%)	400 (10·9%)	272 (10·4%)	0·4865
Very high	566 (9%)	338 (9·2%)	228 (8·7%)	0·4650

Treatments*: 2,047 out of 5,336 patients (38·4%) received red blood cell transfusions; 854 patients (16%) were treated with erythroid stimulating agents; 1,238 patients (16·1%) were treated with hypomethylating agents; 369 patients (4·8%) were treated with AML-like chemotherapy; 300 patients (3·9%) received allogeneic stem cell transplantation. A higher prevalence of transfusion dependency was noticed in men vs. women (P=0·023).*

Supplementary Table_1D_SF1. *Demographic, haematological and clinical features of 1,185 patients from Düsseldorf MDS registry, collected at the time of diagnosis and information on treatment.*

Variable	All patients	Men	Women	P value
**Patients (number. %)**	1,185	725 (61·2%)	460 (38·8%)	<0·0001
**Age (years)**	67 (17-94)	67 (17-94)	66 (17-89)	0·0343
**AGE categories**	1,185	725 (61·2%)	460 (38·8%)	-
<50	136 (11·5%)	65 (9%)	71 (15·4%)	0·0007
50-59	213 (18%)	134 (18·5%)	79 (17·2%)	0·5676
60-69	372 (31·4%)	228 (31·4%)	144 (31·3%)	0·9585
70-79	369 (31·1%)	232 (32%)	137 (29·8%)	0·4220
>80	95 (8%)	66 (9·1%)	29 (6·3%)	0·0839
**Haemoglobin (Hb, g/dL)**	9·6 (4·2-16·9)	9·7 (4·2-16·9)	9·4 (4·3-14·1)	0·010
**Haemoglobin categories**	1,110	682 (61·4%)	428 (38·6%)	-
Normal Hb values	103 (9·3%)	63 (9·2%)	40 (8·3%)	0·9518
<Normal values -11 g/dl	190 (17·1%)	136 (19·9%)	54 (12·6%)	0·0016
<11-10 g/dl	193 (17·4%)	118 (17·3%)	75 (17·5%)	0·9246
<10-9 g/dl	166 (15%)	93 (13·6%)	73 (17·1%)	0·1201
<9-8 g/dl	245 (22·1%)	146 (21·4%)	99 (23·1%)	0·5006
<8 g/dl	213 (19·2%)	126 (18·5%)	87 (20·3%)	0·4459
**RBC transfusion dependency**	433/1,110 (39%)	263/682 (38·6%)	170/428 (39·7%)	0·7007
**Neutrophils (x10^9/L)**	1·75 (0·09-32·83)	1·71 (0·1-26)	1·77 (0·09-32·83)	0·8132
**Platelets(x10^9/L)**	115 (2-1,194)	103 (3-999)	132 (2-1,194)	0·0040
**WHO category***	1,185	725 (61·2%)	460 (38·8%)	-
MDS with 5q-	98 (8·3%)	31 (4·3%)	67 (14·6%)	<0·0001
MDS-SLD	64 (5·4%)	39 (5·4%)	25 (5·4%)	0·9672
MDS-RS-SLD	48 (4·1%)	27 (3·7%)	21 (4·6%)	0·4744
MDS-MLD	463 (39·1%)	310 (42·8%)	153 (33·3%)	0·0011
MDS-RS-MLD	69 (5·8%)	39 (5·4%)	30 (6·5%)	0·4133
MDS-EB1	197 (16·6%)	123 (17%)	74 (16%)	0·6923
MDS-EB2	246 (20·8%)	156 (21·5%)	90 (19·6%)	0·4196
MDS-U	0	0	0	-
**IPSSR cytogenetic risk group**	1,076	661 (61·4%)	415 (38·6%)	-
Very good	43 (4%)	42 (6·4%)	1 (0·2%)	<0·0001
Good	754 (70·1%)	447 (67·6%)	307 (74%)	0·0269
Intermediate	61 (5·7%)	40 (6·1%)	21 (5·1%)	0·4939
Poor	46 (4·3%)	25 (3·8%)	21 (5·1%)	0·3133
Very poor	172 (16%)	107 (16·2%)	65 (15·7%)	0·8192
**IPSSR risk group**	910	557 (61·2%)	353 (38·8%)	-
Very low	130 (14·3%)	87 (15·6%)	43 (12·2%)	0·1489
Low	332 (36·2%)	197 (35·4%)	135 (38·2%)	0·3802
Intermediate	194 (21·3%)	122 (21·9%)	72 (20·4%)	0·5889
High	117 (12·9%)	67 (12%)	50 (14·2%)	0·3486
Very high	137 (15·1%)	84 (15·1%)	53 (15%)	0·9782

Treatments. *432 out of 1,110 patients (38·9%) received red blood cell transfusions; 109 patients (9·8%) were treated with hypomethylating agents; 152 patients (12·8%) received allogeneic stem cell transplantation. No significant difference was noticed in the prevalence of different treatment strategies between men and women (not shown).*

** The diagnosis of myeloid neoplasm was formulated according to the criteria of the 2016 revision of WHO classification of myeloid neoplasms. Peripheral blood and bone marrow dysplasia was performed using established consensus criteria. (MDS with 5q-, MDS with isolated deletion of long arm of chromosome 5; MDS-SLD, MDS with single lineage dysplasia; MDS-MLD, MDS with multilineage dysplasia; MDS-RS-SLD, MDS with ring sideroblasts and single lineage dysplasia; MDS-RS-MLD, MDS with ring sideroblasts and multilineage dysplasia; MDS-EB1, MDS with excess of blasts, type 1; MDS-EB1, MDS with excess of blasts, type 2; IPSS-R, Revised International Prognostic Scoring System).*

Supplementary Figure_1_SF1. *Probability of survival (since the time of diagnosis) of MDS patients belonging to EuroMDS cohort, IWG-PM cohort, registry of Spanish MDS Group (GESMD) and Düsseldorf MDS registry according to 2016 WHO categories (A, B, C and D, respectively) and to IPSS-R risk groups (E, F, G and H, respectively). (MDS with 5q-, MDS with isolated deletion of long arm of chromosome 5; MDS-SLD, MDS with single lineage dysplasia; MDS-MLD, MDS with multilineage dysplasia; MDS-RS-SLD, MDS with ring sideroblasts and single lineage dysplasia; MDS-RS-MLD, MDS with ring sideroblasts and multilineage dysplasia; MDS-EB1, MDS with excess of blasts, type 1; MDS-EB1, MDS with excess of blasts, type 2; MDS-U, unclassified MDS; IPSS-R, Revised International Prognostic Scoring System).*

A. Probability of overall survival of EuroMDS cohort according to WHO categories (P<0·0001)

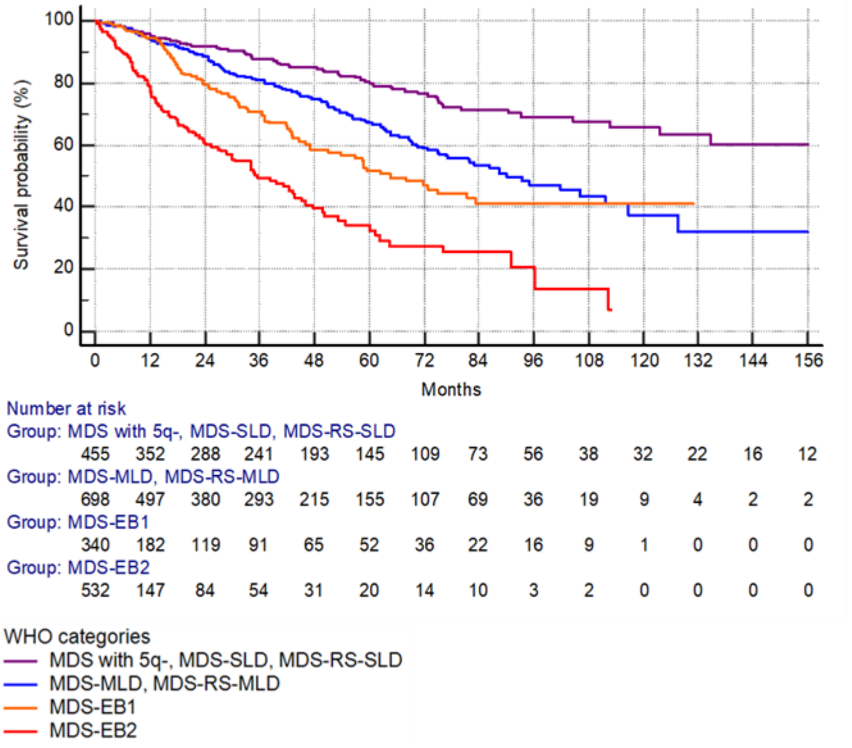

B. Probability of overall survival of IWG-PM cohort according to WHO categories (P<0·0001)

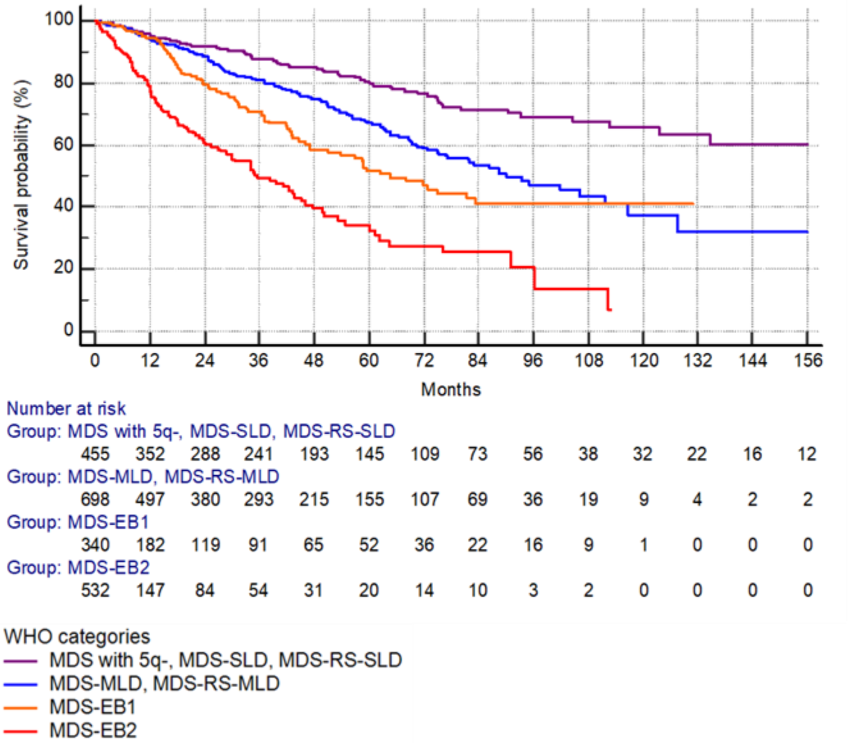

C. Probability of overall survival of Spanish MDS Group (GESMD) registry according to WHO categories (P<0·0001)

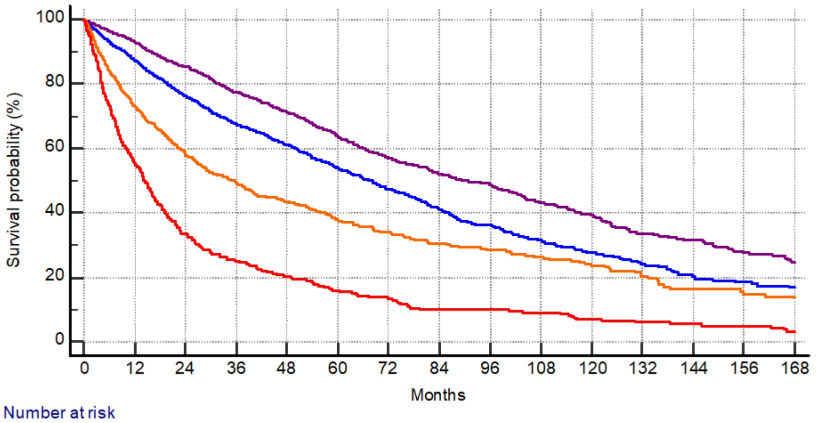

Number at risk

Group	0	12	24	36	48	60	72	84	96	108	120	132	144	156	168
Group: MDS with 5q-, MDS-SLD, MDS-RS-SLD	2255	1852	1446	1134	909	710	555	436	352	270	207	148	115	83	65
Group: MDS-MLD, MDS-RS-MLD	3117	2201	1573	1116	839	625	454	311	239	175	132	92	66	50	40
Group: MDS-EB1	1257	590	374	260	198	157	131	98	80	68	50	32	22	17	13
Group: MDS-EB2	1042	277	136	86	64	46	37	27	25	22	16	12	10	9	5

WHO categories
- MDS with 5q-, MDS-SLD, MDS-RS-SLD, MDS-U
- MDS-MLD, MDS-RS-MLD
- MDS-EB1
- MDS-EB2

D. Probability of overall survival of Düsseldorf MDS registry according to WHO categories (P<0·0001)

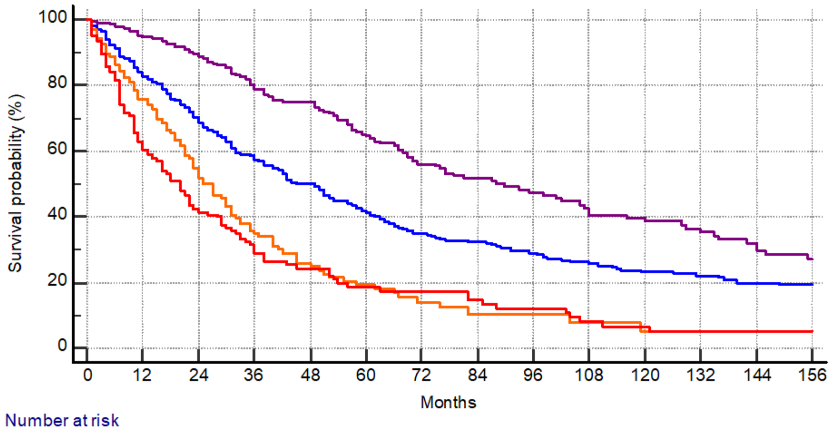

Number at risk

Group	0	12	24	36	48	60	72	84	96	108	120	132	144	156
Group: MDS with 5q-, MDS-SLD, MDS-RS-SLD	210	182	158	135	124	97	78	65	52	40	37	32	26	22
Group: MDS-MLD, MDS-RS-MLD	531	347	250	193	153	112	88	75	66	56	48	43	33	30
Group: MDS-EB1	197	108	64	36	23	17	9	5	4	3	2	2	2	2
Group: MDS-EB2	246	93	56	33	23	14	13	11	9	6	5	3	3	3

WHO categories
- MDS with 5q-, MDS-SLD, MDS-RS-SLD
- MDS-MLD, MDS-RS-MLD
- MDS-EB1
- MDS-EB2

E. Probability of overall survival of EuroMDS cohort according to IPSS-R categories (P<0·0001)

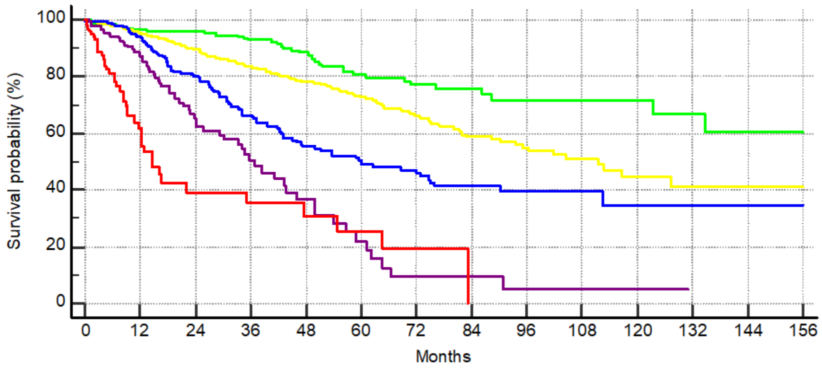

Number at risk

Group	0	12	24	36	48	60	72	84	96	108	120	132	144	156
Group: Very Low	243	202	171	142	109	82	57	42	32	22	16	10	7	5
Group: Low	606	467	382	308	242	178	127	81	49	29	16	8	4	4
Group: Intermediate	323	209	148	106	72	58	44	29	15	11	4	3	3	1
Group: High	259	101	44	25	14	7	2	2	1	1	1	0	0	0
Group: Very High	187	29	12	10	6	4	1	0	0	0	0	0	0	0

IPSS-R Categories
- Very Low
- Low
- Intermediate
- High
- Very High

F. Probability of overall survival of IWG-PM cohort according to IPSS-R categories (P<0·0001)

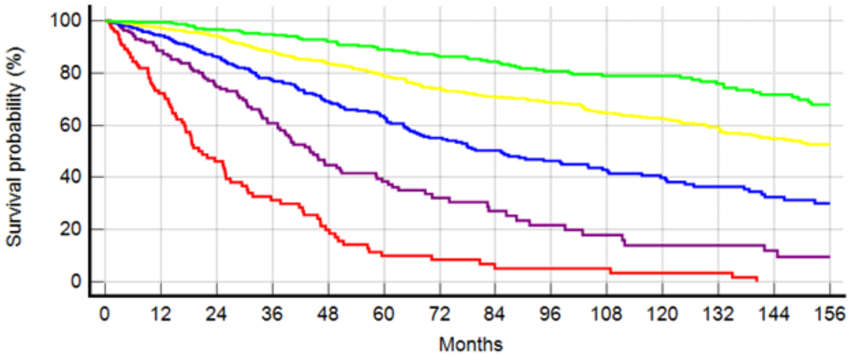

Number at risk

Group	0	12	24	36	48	60	72	84	96	108	120	132	144	156
Group: High	282	135	94	68	44	35	20	15	12	9	7	7	6	4
Group: Intermediate	451	313	241	192	155	130	107	91	75	59	49	40	29	22
Group: Low	824	705	611	523	455	395	343	305	266	221	187	153	130	115
Group: Very high	229	71	35	23	14	7	6	3	3	3	2	2	0	0
Group: Very low	339	306	276	244	219	202	186	165	146	128	113	95	81	67

IPSS-R Categories
- Very Low
- Low
- Intermediate
- High
- Very High

G. Probability of overall survival of Spanish MDS Group (GESMD) registry according to IPSS-R categories (P<0·0001)

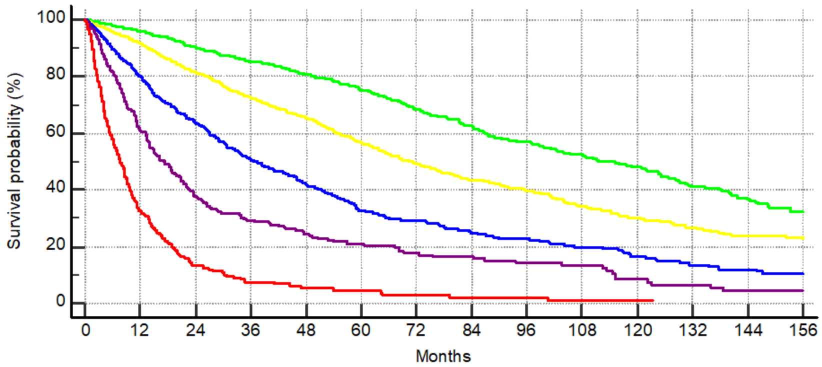

Number at risk

Group	0	12	24	36	48	60	72	84	96	108	120	132	144	156
Group: Very Low	1562	1309	1018	796	632	512	393	291	231	176	127	87	63	40
Group: Low	2426	1855	1349	967	729	532	387	280	213	157	117	84	62	48
Group: Intermediate	1068	571	349	220	155	106	83	56	47	37	30	19	15	12
Group: High	672	198	94	62	43	33	27	23	18	15	8	6	4	4
Group: Very High	565	75	25	11	8	5	3	2	2	1	1	0	0	0

IPSS-R Categories
- Very Low
- Low
- Intermediate
- High
- Very High

H. Probability of overall survival of Düsseldorf MDS registry according to IPSS-R categories (P<0·0001)

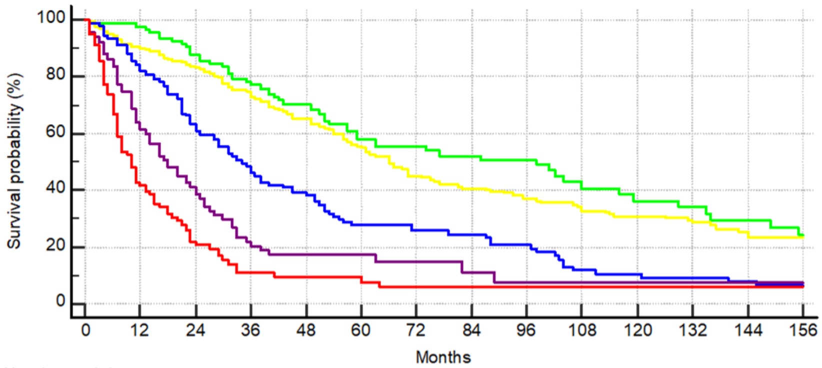

Number at risk

Group	0	12	24	36	48	60	72	84	96	108	120	132	144	156
Group: Very Low	130	105	85	72	60	41	36	33	30	19	16	15	12	10
Group: Low	331	258	213	171	145	111	83	66	55	48	43	35	26	24
Group: Intermediate	194	119	79	54	41	25	22	20	16	9	8	7	6	5
Group: High	117	48	28	13	7	7	6	3	2	2	2	2	2	2
Group: Very High	137	39	17	7	6	4	3	3	3	3	3	2	1	1

IPSS-R Categories
- Very Low
- Low
- Intermediate
- High
- Very High

SUPPLEMENTARY_FILE_2 (SF2) – Cytogenetics and mutation screening

At diagnosis, cytogenetic analysis was performed using standard G-banding and karyotypes were classified using the International System for Cytogenetic Nomenclature Criteria.
In patients belonging to EuroMDS cohort, we analyzed in addition somatic mutations in 47 genes related to myeloid neoplasms, obtained by analyzing tumor DNA derived from bone marrow mononuclear cells (94% of cases) or peripheral blood granulocytes (6% of cases). Sample for DNA sequencing was collected within 30 days from diagnosis for 93% of cases (in all cases within 6 months since the date of diagnosis).
Sequencing strategy was performed using a targeted multiplexed amplicon-based approaches (Illumina, San Diego, CA, USA) starting from genomic DNA; the resulting libraries were sequenced on Illumina platforms (NextSeq500) in paired-end mode. Targeted regions are listed in *Supplementary Table_1_SF2* (see below).
Variants with a variant allele frequency (VAF) lower than 0.01 and/or variants with a coverage <200x were filtered out. Functionally annotated variants were then also excluded based on the information retrieved from public databases (dbSNP, gnomAD) and the expected germ line allele frequency. Single nucleotide polymorphisms (SNP) were annotated according to the NCBI dbSNP (http://www.ncbi.nlm.nih.gov/snp; Build 137) and gnomAD (http://gnomad.broadinstitute.org; gnomAD r2.0.1) databases.
The remaining variants were considered as possible somatic mutations and their pathogenic value was evaluated in order to differentiate known and putative pathogenic mutations from variants of unclear significance by using a multi-step algorithm:
1) All variants (missense, in-frame insertions/deletions, frameshift, nonsense and splice site) were considered pathogenic if they were previously reported in the publicly accessible Catalogue Of Somatic Mutations In Cancer (COSMIC, version 69) (http://cancer.sanger.ac.uk/cancergenome/projects/cosmic) at least in two hematological sample.
2) Internal-tandem-duplication of FLT3 and in-frame insertions/deletions of CALR (exon 9) genes were included as pathogenic variants.
3) Loss of function mutations (Nonsense, frameshift and splice site) were considered pathogenic.
4) Missense variants and in-frame insertions/deletions not fulfilling these criteria were individually assessed based on the available data from COSMIC (the tissues they were found in, whether any other COSMIC variants were reported affecting the same amino-acid positions or were within 3 amino-acids) and their predicted functional consequences using the Mutation Taster algorithm (http://www.mutationtaster.org).
5) Nonsynonymous variants not fulfilling these criteria were then classified on the basis of their functional interpretation using in silico prediction effect by SIFT 1.03 (http://sift.jcvi.org), PolyPhen 2.0 (http://genetics.bwh.harvard.edu/pph2) and MutationTaster 1.0 algorithms (http://www.mutationtaster.org). Variants with less than 2/3 deduced damaging consequences on the amino acid level were discarded.
6) Additionally, TP53 variants were verified using the IARC repository (https://p53.iarc.fr/).
Variants that did not satisfy any of the above criteria were not considered as pathogenic mutations in downstream analyses

Supplementary Table 1_SF2. *Panel of sequenced genes in the EuroMDS and IWG-PM cohorts. The column description from left to right: Gene, name of gene; Pathway, main biological pathways in which the gene is involved or has a determinant function; NCBI gene ID, National Center for Biotechnology Information gene ID; Position, Chromosomal location.*

Gene (coding exons and 5 splice sites)	Pathway	NCBI gene ID	Position
ASXL1 (all)	chromatin & histones modifier	171023	20q11.1
BCOR (2-15)	chromatin & histones modifier	54880	Xp11.14
BCORL1 (1-12)	chromatin & histones modifier	14616	Xq26.1
EZH2 (2-8, 11-20)	chromatin & histones modifier	2146	7q35-36
KDM6A/UTX (1-29)	chromatin & histones modifier	7403	Xp11.2
RAD21 (2-14)	cohesin complex	5885	8q24
SMC1A (2, 11, 16-17)	cohesin complex	8243	Xp11.22
SMC3 (10, 13, 19, 23, 25, 28)	cohesin complex	9126	10q25.2
STAG2 (3-35)	cohesin complex	10735	Xq25
DNMT3A (2-23)	DNA methylation	1788	2p23
IDH1 (4)	DNA methylation	3417	2q33.3
IDH2 (4)	DNA methylation	3418	15q26.1
TET2 (all)	DNA methylation	54790	4q24
PRPF40B (2-26)#	RNA splicing	25766	12q13.12
SF3B1 (10-16)	RNA splicing	23451	2q33.1
SRSF2 (1)	RNA splicing	6427	17q25.1
U2AF1 (2, 6-8)	RNA splicing	7307	21q22.3
ZRSR2 (all)	RNA splicing	8233	Xp22.1
BRAF (15)	signalling	673	7q34
CALR (9)	signalling	811	19p13.13
CBL (7-9)	signalling	867	11q23.3
CBLB (9-11)#	signalling	868	11q13.11
CSF3R (all)	signalling	412	1p34.3
DDX41 (all)*	signalling	51428	5q35.3
FBXW7 (8-12)#	signalling	55294	4q31.3
FLT3 (13-16, 20)	signalling	2322	13q12
GNAS (8-9)	signalling	2778	20q13.3
GNB1 (3-11)	signalling	2782	1p36.33
JAK2 (all)	signalling	3717	9p24
KIT (2, 8-11, 13, 17-18)	signalling	3815	4q12
KRAS (2-5)	signalling	3845	12p12.1
MPL (10)	signalling	4352	1p34
NF1 (1-58)	signalling	42292	7q11.2
NOTCH1 (24-28, 34)	signalling	4851	9q34.3
NRAS (2-5)	signalling	4893	1p13.2
PIGA (2, 6)#	signalling	14165	Xp22.2
PPM1D (all)	signalling	11625	17q23.2
PTPN11 (1-15)	signalling	5781	12q24.1
ATRX (8-31)	transcription regulation	546	Xq21.1
CEBPA (1)	transcription regulation	1050	19q13.1
ETV6 (all)	transcription regulation	2120	12p13.2
GATA2 (2-6)	transcription regulation	2624	3q21.3
NPM1 (10-12)	transcription regulation	4869	5q35
PHF6 (2-10)	transcription regulation	84295	Xq26.2
RUNX1 (all)	transcription regulation	861	21q22.3
SETBP1 (4)	transcription regulation	45859	18q12.3
TP53 (all)	tumor suppressor	7157	17p13.1
WT1 (all)	tumor suppressor	7490	1p13

*# Data available only in EuroMDS cohort; * Data available only in IWG-PM cohort*

SUPPLEMENTARY_FILE_4 (SF4) – Genomic landscape of MDS by sex

Supplementary Table_1_SF4. *Prevalence of chromosomal abnormalities in patients from EuroMDS cohort stratified by sex. For each class of comparisons, p-values were adjusted for multiple testing using the Benjamini-Hochberg procedure.*

Karyotype	All patients	Men	Women	P value	Adjusted P value
**Available**	1,789/2025 (88·3%)	1,075/1,205 (89·2%)	723/820 (88·2%)	-	-
**Normal**	1,173 (65·6%)	706 (65·7%)	467 (64·6%)	0·8323	0.9940
**Complex karyotype (>3 abnormalities)**	137 (7·7%)	72 (6·7%)	65 (9·0%)	0·0716	0.4025
**Chromosomal abnormalities**	616	369 (59·9%)	256 (40·1%)		
Del(5q)	154 (25·0%)	60 (16·3%)	94 (36·7%)	<0·0001	<0·0001
Loss chr 7/del(7q)	45 (7·3%)	27 (7·3%)	18 (7·0%)	0·9836	0.9925
Gain chr 8	64 (10·4%)	35 (9·5%)	29 (11·3%)	0·4121	0.7714
Del(9q)	4 (0·6%)	2 (0·5%)	2 (0·8%)	0·6926	0.9902
Del(11q)	10 (1·2%)	6 (1·6%)	4 (1·6%)	0·9902	0.9954
Del(12p)/t(12p)	10 (1·2%)	6 (1·6%)	4 (1·6%)	0·9935	0.9981
Loss chr 13/del(13q)	8 (1·3%)	5 (1·4%)	3 (1·2%)	0·8859	0.9965
Isochr 17/t(17p)	6 (1·0%)	4 (1·1%)	2 (0·8%)	0·7335	0.9921
Del(20q)	25 (4·1%)	18 (4·9%)	7 (2·7%)	0·1825	0.5155
t(3;21)(q26.2;q22.1)	0	0	0	-	-
t(1;3)(p36.3;q21.2)	2 (0·3%)	2 (0·5%)	0	0·2457	0.5125
t(2;11)(p21;q23.3)	3 (0·5%)	3 (0·8%)	0	0·1616	0.5108
inv(3)(q21.3q26.2)/ t(3;3)(q21.3q23.6.2)	4 (0·6%)	0	4 (1·6%)	0·01509	0.1336
t(6;9)(p23;q34) 19	1 (0·2%)	0	1 (0·4%)	0·2210	0.5125
Loss chr Y*	54 (14·6%)	54 (14·6%)	-	-	-
Idic(X)(q13)	1 (0·2%)	0	1 (0·4%)	0·2238	0.5133
Other	157 (25·5%)	103 (27·9%)	54 (21·1%)	0·5125	0.8742

**Only for men*

Supplementary Table_2_SF4. *Prevalence of chromosomal abnormalities in patients from IWG-PM cohort stratified by sex. For each class of comparisons, p-values were adjusted for multiple testing using the Benjamini-Hochberg procedure.*

Karyotype	All patients	Men	Women	P value	Adjusted P value
**Available**	2,323 (97·3%)	1406 (97·5%)	917 (97·3%)	0·4924	0.6620
**Normal**	1,371 (59%)	848 (60·3%)	558 (57%)	0·1242	0.3109
**Complex karyotype (>3 abnormalities)**	249 (10·7%)	153 (10·9%)	96 (10·5%)	0·7561	0.8331
**Chromosomal abnormalities**	933 (41%)	553 (39·7%)	380 (43%)		
Del(5q)	368 (16%)	150 (10·7%)	218 (24%)	<0·0001	<0·0001
Loss chr 7/del(7q)	192 (8·3%)	119 (8·5%)	73 (8%)	0·6952	0.8255
Gain chr 8	166 (7·2%)	107 (7·7%)	59 (6·5%)	0·2916	0.5324
Del(9q)	53 (2·3%)	33 (2·4%)	20 (2·2%)	0·8005	0.8314
Del(11q)	61 (2·6%)	31 (2·2%)	30 (3·3%)	0·1142	0.3174
Del(12p)/t(12p)	68 (3%)	45 (3·2%)	23 (2·5%)	0·3455	0.5388
Loss chr 13/del(13q)	52 (2·3%)	37 (2·7%)	15 (1·7%)	0·1239	0.3152
Isochr 17/t(17p)	66 (2·9%)	46 (3·3%)	20 (2·2%)	0·1361	0.3142
Del(20q)	141 (6·1%)	100 (7·2%)	41 (4·5%)	0·0102	0.0641
t(3;21)(q26.2;q22.1)	2 (0·1%)	0	2 (0·2%)	0.0821	0.3136
t(1;3)(p36.3;q21.2)	1 (<0·1%)	0	1 (0·1%)	0·2136	0.4451
t(2;11)(p21;q23.3)	3 (0·4%)	2 (0·1%)	1 (0·1%)	0·8369	0.8398
inv(3)(q21.3q26.2)/ t(3;3)(q21.3q23.6.2)	9 (0·4%)	4 (0·3%)	5 (0·6%)	0·3254	0.5264
t(6;9)(p23;q34) 19	4 (0·2%)	3 (0·2%)	1 (0·1%)	0·5641	0.7125
Loss chr Y*	116 (8·3%)	116 (8·3%)	-	-	-

Idic(X)(q13)<br>Other	22 (1%)<br>391 (17%)	3 (0·2%)<br>245 (17·6%)	19 (2·1%)<br>146 (16·1%)	<0·0001<br>0·3655	<0·0001<br>0.5342

*Only for men

Supplementary Table_3_SF4. *Prevalence of mutated genes in patients from EuroMDS cohort stratified by sex. For each class of comparisons, p-values were adjusted for multiple testing using the Benjamini-Hochberg procedure.*

Variable	All patients	Men	Women	P value	Adjusted P value
**N° mutated pts**	1,623/2,025 (80·1%)	998/1,205 (82·8%)	625/820 (76·2%)	<0·0001	<0·0001
**Median number of mutation**	2 (1-17)	3 (1-13)	2 (1-17)	0·0021	0.0145
***ASXL1* mutated patients**	345 (17·9%)	241 (20·8%)	104 (13·4%)	<0·0001	<0·0001
***ATRX* mutated patients**	14 (1·0%)	8 (0·9%)	6 (1·0%)	0·8125	0.9041
***BCOR* mutated patients**	83 (4·3%)	39 (3·4%)	44 (5·7%)	0·01531	0.0625
***BCORL1* mutated patients**	19 (1·3%)	13 (1·5%)	6 (1·0%)	0·4645	0.6625
***BRAF* mutated patients**	4 (0·3%)	2 (0·2%)	2 (0·3%)	0·6712	0.8205
***CALR* mutated patients**	0	0	0	-	-
***CBL* mutated patients**	83 (4·3%)	48 (4·2%)	35 (4·5%)	0·7057	0.8321
***CBLB* mutated patients**	1 (0·1%)	1 (0·1%)	0	0·4225	0.6351
***CEBPA* mutated patients**	22 (1·1%)	15 (1·3%)	7 (0·9%)	0·4231	0.6322
***CSF3R* mutated patients**	17 (2%)	10 (2·1%)	7 (1·9%)	0·9221	0.9548
***DNMT3A* mutated patients**	245 (12·7%)	121 (10·5%)	124 (16·0%)	<0·0001	<0·0001
***ETV6* mutated patients**	39 (2·0%)	28 (2·4%)	11 (1·4%)	0·1253	0.3621
***EZH2* mutated patients**	107 (5·5%)	69 (6·0%)	38 (4·9%)	0·3167	0.6240
***FBXW7* mutated patients**	12 (0·8%)	4 (0·5%)	8 (1·4%)	0·0621	0.2206
***FLT3* mutated patients**	36 (1·9%)	19 (1·6%)	17 (2·2%)	0·3806	0.62
***GATA2* mutated patients**	17 (0·9%)	7 (0·6%)	10 (1·3%)	0·1254	0.3654
***GNAS* mutated patients**	15 (1·0%)	7 (0·8%)	8 (1·4%)	0·2809	0.6028
***GNB1* mutated patients**	5 (0·4%)	4 (0·5%)	1 (0·2%)	0·3734	0.6294
***IDH1* mutated patients**	54 (2·8%)	32 (2·8%)	22 (2·8%)	0·9302	0.9531
***IDH2* mutated patients**	80 (4·1%)	59 (5·1%)	21 (2·7%)	<0·0001	<0·0001
***JAK2* mutated patients**	77 (4·0%)	48 (4·2%)	29 (3·7%)	0·6555	0.8227
***KIT* mutated patients**	20 (1·0%)	10 (0·9%)	10 (1·3%)	0·3721	0.6251
***KRAS* mutated patients**	54 (2·8%)	38 (3·3%)	16 (2·1%)	0·1103	0.3618
***MPL* mutated patients**	35 (2·3%)	23 (2·5%)	12 (2%)	0·5287	0.7135
***NF1* mutated patients**	57 (3·0%)	34 (2·9%)	23 (3%)	0·9821	0.9851
***NOTCH1* mutated patients**	14 (1·0%)	10 (1·1%)	4 (0·7%)	0·3965	0.6221
***NPM1* mutated patients**	30 (1·6%)	14 (1·2%)	16 (2·1%)	0·1428	0.3701
***NRAS* mutated patients**	69 (3·6%)	45 (3·9%)	24 (3·1%)	0·3577	0.6254
***PHF6* mutated patients**	35 (1·8%)	23 (2·0%)	12 (1·5%)	0·4732	0.6691
***PIGA* mutated patients**	4 (0·3%)	2 (0·2%)	2 (0·2%)	0·6751	0.8205
***PPM1D* mutated patients**	5 (0·4%)	3 (0·3%)	2 (0·2%)	0·9032	0.9564
***PRPF40B* mutated patients**	8 (0·5%)	4 (0·5%)	4 (0·7%)	0·5504	0.7342
***PTPN11* mutated patients**	39 (2·0%)	30 (2·6%)	9 (1·2%)	0·0287	0.1151
***RAD21* mutated patients**	15 (1·0%)	7 (0·8%)	8 (1·4%)	0·2851	0.6005
***RUNX1* mutated patients**	219 (11·3%)	141 (12·2%)	78 (10·1%)	0·1439	0.3724
***SETBP1* mutated patients**	28 (3·3%)	17 (3·5%)	11 (3·1%)	0·7325	0.8350
***SF3B1* mutated patients**	497 (25·7%)	287 (24·8%)	210 (27·1%)	0·2705	0.6018
***SMC1A* mutated patients**	12 (0·8%)	9 (1%)	3 (0·5%)	0·2981	0.6024
***SMC3* mutated patients**	16 (1·1%)	10 (1·2%)	6 (1·1%)	0·8654	0.9443
***SRSF2* mutated patients**	292 (15·1%)	204 (17·6%)	88 (11·3%)	<0·0001	<0·0001
***STAG2* mutated patients**	111 (5·7%)	71 (6·1%)	40 (5·2%)	0·3621	0.6214
***TET2* mutated patients**	464 (24%)	304 (26·3%)	160 (20·6%)	0·0051	0.0310
***TP53* mutated patients**	154 (8%)	76 (6·6%)	78 (10·1%)	0·0063	0.0301
***U2AF1* mutated patients**	127 (6·6%)	91 (7·9%)	36 (4·6%)	0·0052	0.0325
***UTX* mutated patients**	30 (1·6%)	17 (1·5%)	13 (1·7%)	0·7254	0.8304
***WT1* mutated patients**	17 (0·9%)	8 (0·7%)	9 (1·2%)	0·2821	0.6025
***ZRSR2* mutated patients**	115 (6·0%)	104 (9·0%)	11 (1·4%)	<0·0001	<0·0001
**Functional pathways (as defined according to Reference 14)**<br>Chromatin & histones modifier	481 (24·9%)	313 (27·1%)	168 (21·6%)	0·0071	0.0205

Cohesin complex	149 (7·7%)	95 (8·2%)	54 (7·0%)	0·3142	0.4221
DNA methylation	742 (38·4%)	467 (40·4%)	275 (35·4%)	0·0283	0.0455
RNA splicing	969 (50·2%)	636 (55·0%)	333 (42·9%)	<0·0001	<0.0001
Signaling	429 (22·2%)	257 (22·2%)	172 (22·2%)	0·9735	0.9721
Transcription regulation	355 (18·4%)	220 (19·0%)	135 (17·4%)	0·3662	0.4254
Tumor suppressor	170 (8·8%)	83 (7·2%)	87 (11·2%)	0·0025	0.0073

Supplementary Table_4_SF4. *Prevalence of mutated genes in patients from IWG-PM cohort stratified by sex. For each class of comparisons, p-values were adjusted for multiple testing using the Benjamini-Hochberg procedure.*

Variable	All patients	Men	Women	P value	Adjusted P value
**N° mutated pts**	2,137/2,387 (89·5%)	1,335/1,442 (92·6%)	802/945 (84·9%)	<0·0001	<0·0001
**Median number of mutation**	2 (0-10)	3 (0-10)	2 (0-10)	<0·0001	<0·0001
***ASXL1* mutated patients**	576 (24·1%)	418 (29%)	158 (16·7%)	<0·0001	<0·0001
***ATRX* mutated patients**	16 (0·7%)	10 (0·7%)	6 (0·6%)	0·8635	0.9025
***BCOR* mutated patients**	134 (5·6%)	79 (5·5%)	55 (5·8%)	0·7241	0.8562
***BCORL1* mutated patients**	38 (1·6%)	25 (1·7%)	13 (1·4%)	0·4907	0.6824
***BRAF* mutated patients**	7 (0·3%)	5 (0·3%)	2 (0·2%)	0·5572	0.7245
***CALR* mutated patients**	5 (0·2%)	2 (0·1%)	3 (0·3%)	0·3531	0.5531
***CBL* mutated patients**	95 (4%)	56 (3·9%)	39 (4·1%)	0·7763	0.8852
***CEBPA* mutated patients**	56 (2·2%)	39 (2·7%)	13 (1·4%)	0·0303	0.0924
***CSF3R* mutated patients**	16 (0·7%)	12 (0·8%)	4 (0·4%)	0·2331	0.4254
DDX41 mutated patients	87 (3·6%)	67 (4·6%)	20 (2·1%)	0·0012	0.0062
***DNMT3A* mutated patients**	419 (17·6%)	226 (15·7%)	193 (20·4%)	0·0034	0.0113
***ETV6* mutated patients**	47 (2%)	31 (2·1%)	16 (1·7%)	0·4321	0.6541
***EZH2* mutated patients**	139 (5·8%)	100 (6·9%)	39 (4·1%)	0·0045	0.0225
***FLT3* mutated patients**	27 (1·1%)	15 (1%)	12 (1·3%)	0·6067	0.7548
***GATA2* mutated patients**	33 (1·4%)	22 (1·5%)	11 (1·2%)	0·4654	0.6651
***GNAS* mutated patients**	28 (1·2%)	15 (1%)	13 (1·4%)	0·4625	0.6624
***GNB1* mutated patients**	33 (1·4%)	14 (1%)	19 (2%)	0·0332	0.0910
***IDH1* mutated patients**	67 (2·8%)	45 (3·1%)	22 (2·3%)	0·2519	0.4236
***IDH2* mutated patients**	103 (4·3%)	74 (5·1%)	29 (3·1%)	0·0153	0.0415
***JAK2* mutated patients**	47 (2%)	29 (2%)	18 (1·9%)	0·8521	0.9029
***KIT* mutated patients**	15 (0·6%)	9 (0·6%)	6 (0·6%)	0·9735	0.9705
***KRAS* mutated patients**	39 (1·6%)	27 (1·9%)	12 (1·3%)	0·2654	0.4336
***MPL* mutated patients**	48 (2%)	27 (1·9%)	21 (2·2%)	0·5547	0.7152
***NF1* mutated patients**	67 (2·8%)	41 (2·8%)	26 (2·8%)	0·8912	0.9024
***NOTCH1* mutated patients**	1 (<0·1%)	0	1 (0·1%)	0·2224	0.4218
***NPM1* mutated patients**	24 (1%)	11 (0·8%)	13 (1·4%)	0·1451	0.2955
***NRAS* mutated patients**	65 (2·7%)	40 (2·8%)	25 (2·6%)	0·8535	0.9071
***PHF6* mutated patients**	73 (3·1%)	55 (3·8%)	18 (1·9%)	0·0081	0.0321
***PPM1D* mutated patients**	42 (1·8%)	30 (2·1%)	12 (1·3%)	0·1421	0.2910
***PTPN11* mutated patients**	34 (1·4%)	20 (1·4%)	14 (1·5%)	0·8587	0.9014
***RAD21* mutated patients**	21 (0·9%)	14 (1%)	7 (0·7%)	0·5615	0.7120
***RUNX1* mutated patients**	299 (12·5%)	196 (13·6%)	103 (10·9%)	0·0522	0.1357
***SETBP1* mutated patients**	78 (3·3%)	52 (3·6%)	26 (2·8%)	0·2516	0.4247
***SF3B1* mutated patients**	570 (23·9%)	328 (22·7%)	242 (25·6%)	0·1127	0.2714
***SMC1A* mutated patients**	24 (1%)	18 (1·2%)	6 (0·6%)	0·1424	0.2922
***SMC3* mutated patients**	7 (0·3%)	6 (0·4%)	1 (0·1%)	0·1751	0.3461
***SRSF2* mutated patients**	334 (14%)	242 (16·8%)	92 (9·7%)	<0·0001	<0·0001
***STAG2* mutated patients**	228 (9·6%)	164 (11·4%)	64 (6·8%)	<0·0001	<0·0001
***TET2* mutated patients**	652 (27·3%)	419 (29·1%)	233 (24·7%)	0·0182	0.0351
***TP53* mutated patients**	272 (11·4%)	149 (10·3%)	123 (13%)	0·0242	0.0372
***U2AF1* mutated patients**	214 (9%)	167 (11·6%)	47 (5%)	<0·0001	<0·0001
***UTX* mutated patients**	20 (0·8%)	13 (0·9%)	7 (0·7%)	0·6751	0.8132
***WT1* mutated patients**	36 (1·5%)	15 (1%)	21 (2·2%)	0·0211	0.0712
***ZRSR2* mutated patients**	115 (4·8%)	115 (8%)	0	<0·0001	<0·0001
**Functional pathways (as defined according to Ref 14)**					
Chromatin & histones modifier	1,944 (81·4%)	1,122 (77·8%)	822 (87%)	<0·0001	<0·0001
Cohesin complex	277 (11·7%)	201 (13·9%)	76 (8%)	<0·0001	<0·0001
DNA methylation	1,087 (45·6%)	680 (47·2%)	407 (43·1%)	0·0310	0.0442

RNA splicing	1,181 (49·4%)	804 (55·8%)	377 (40%)	<0·0001	<0·0001
Signaling	549 (23%)	339 (23·5%)	210 (22·2%)	0·7534	0.7521
Transcription regulation	521 (21·8%)	350 (24·3%)	171 (18·1%)	<0·0001	<0·0001
Tumor suppressor	327 (12·7%)	138 (9·5%)	144 (15·2%)	<0·0001	<0·0001

Supplementary Figure_1 _SF4. Cumulative counts and densities per age in sex-biased genes in 2,025 MDS patients from EuroMDS_cohort

Women

Men

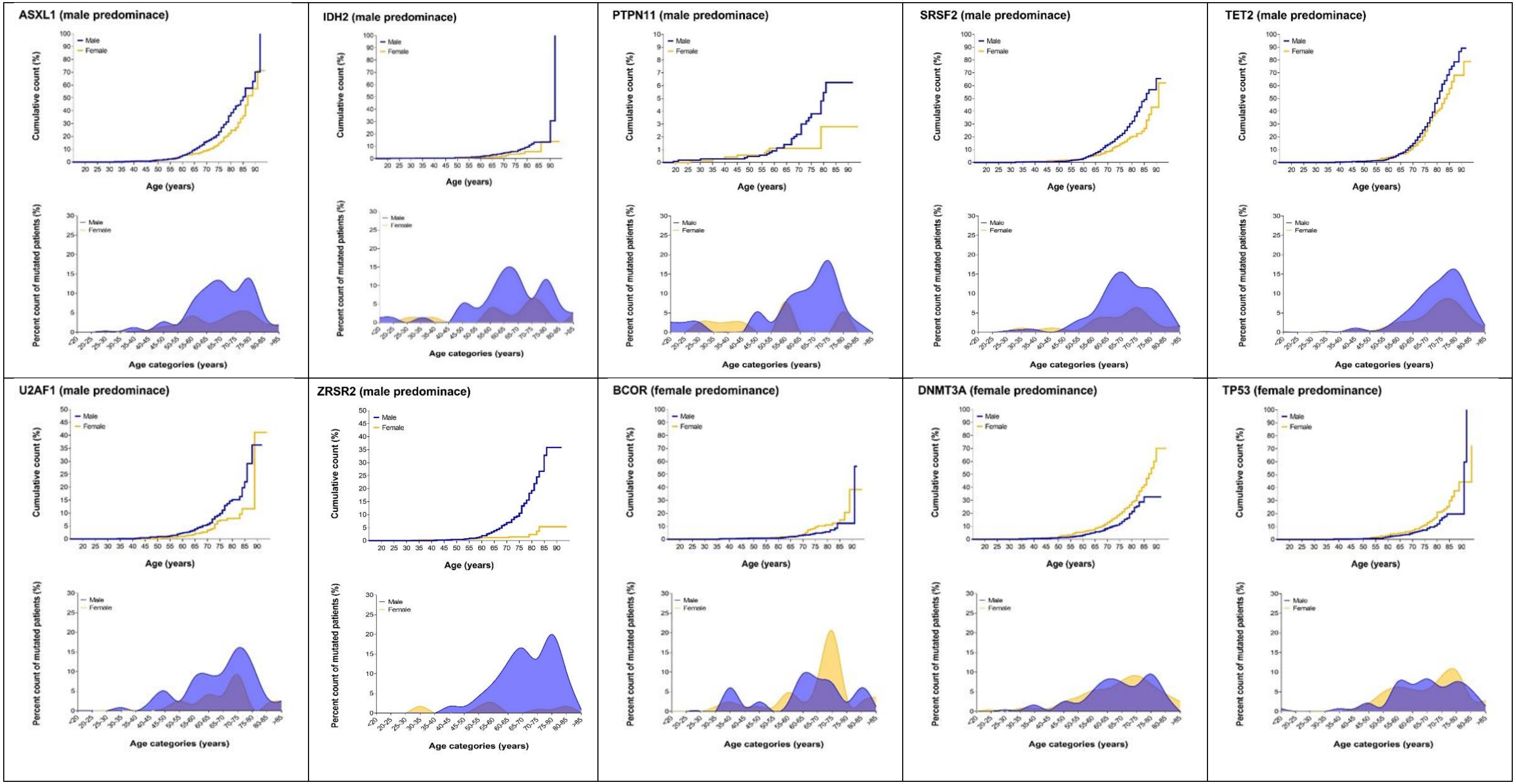

Supplementary Figure_2_SF4. Cumulative counts and densities per age in sex-biased genes in 2,387 MDS patients from IWG-PM_cohort

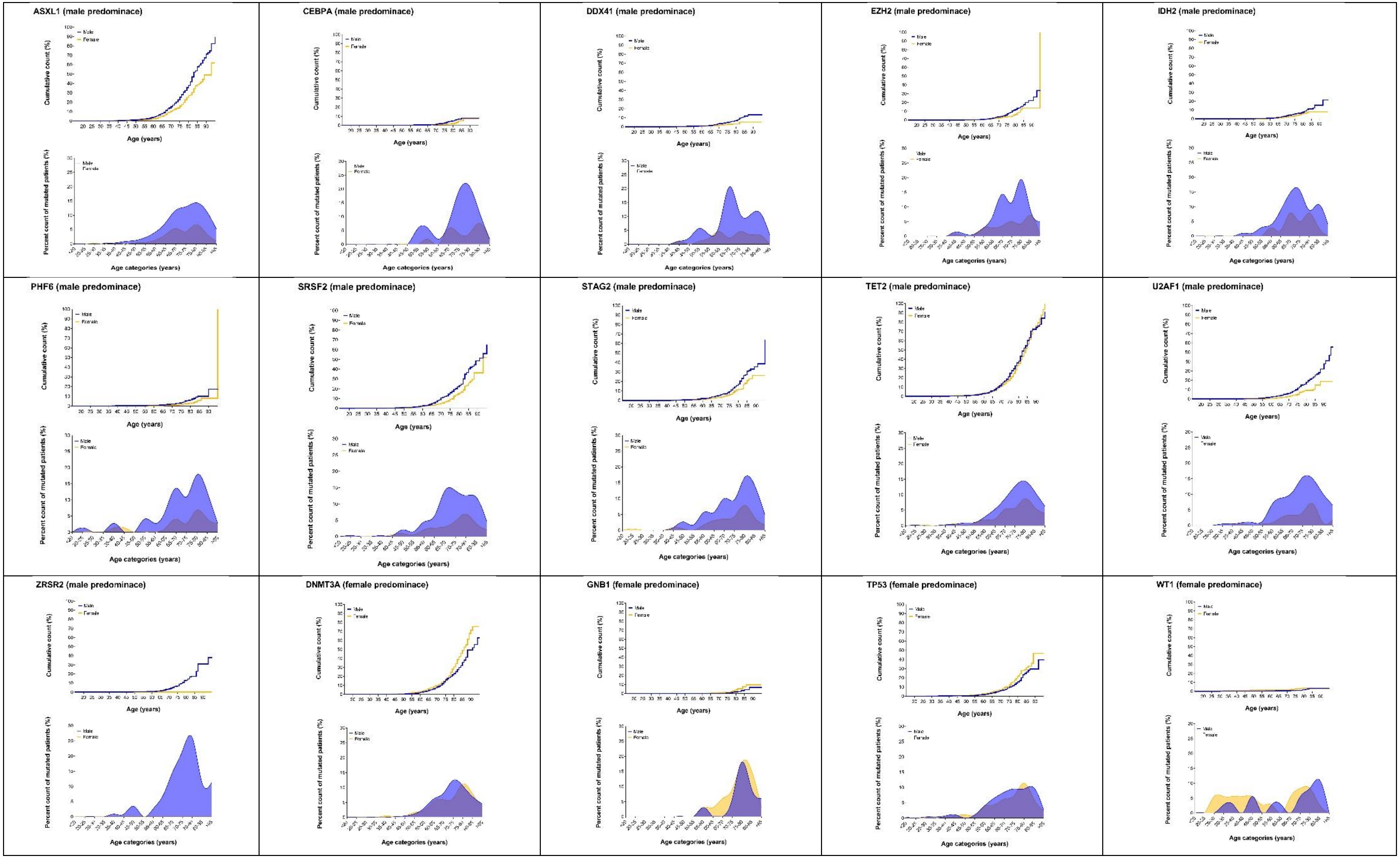

Supplementary Figure_3_SF4. *Distribution of gene mutations in patients from EuroMDS cohort stratified sex across age categories (there are reported only the genes that showed at least 15 mutated patients).*

Women

Men

Gene mutations without sex and age predominance

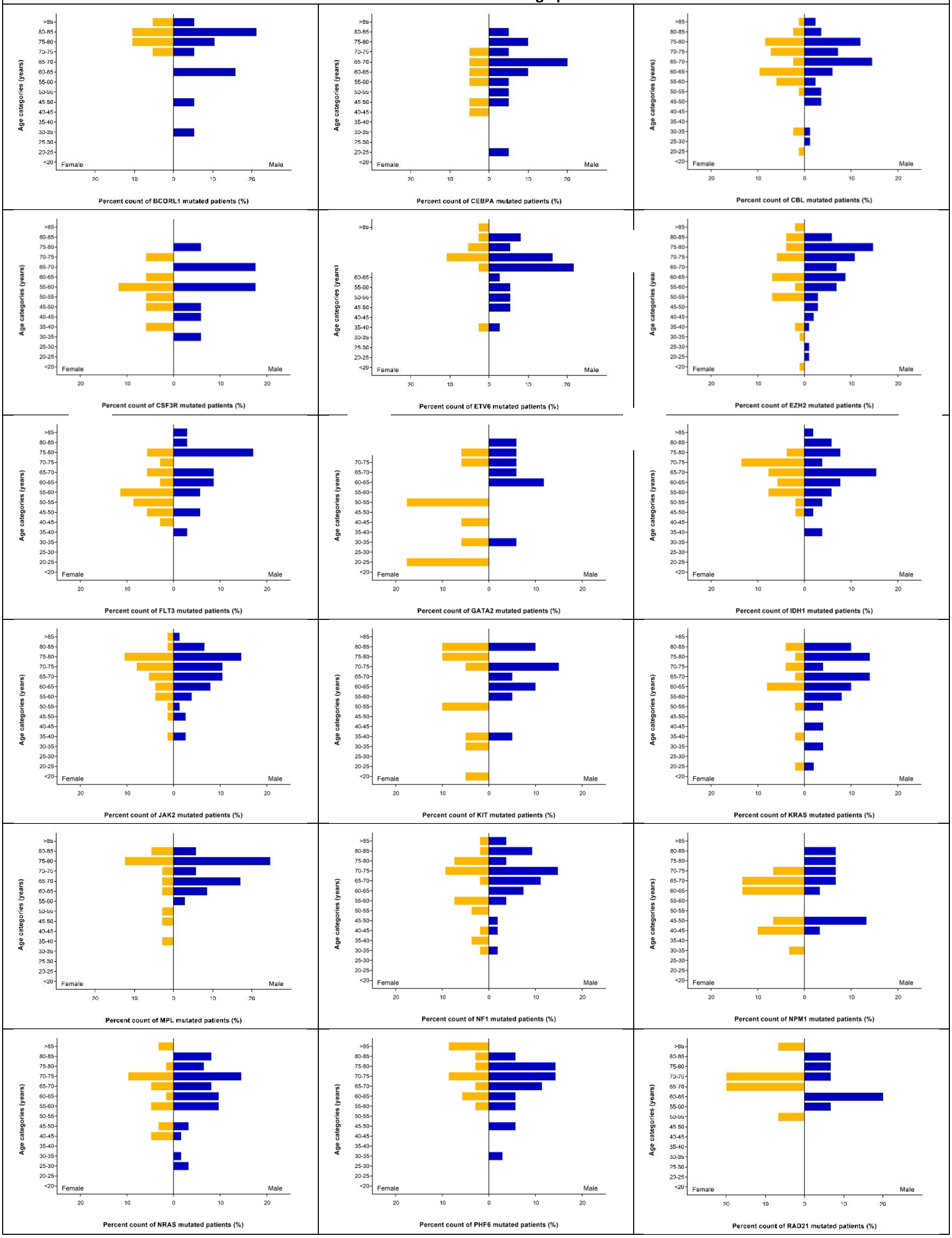

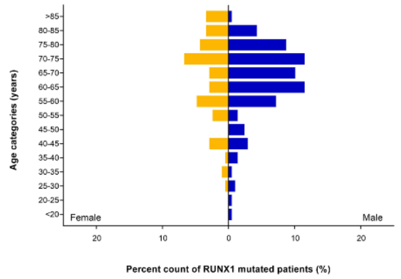

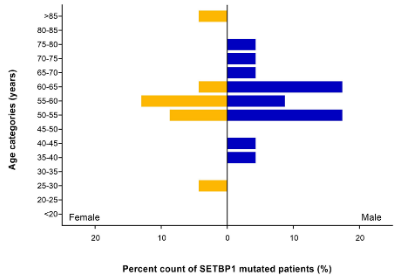

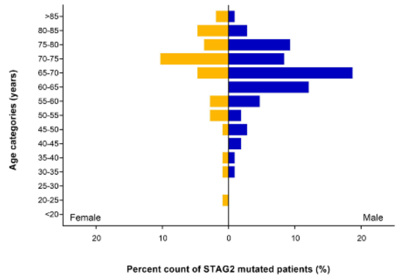

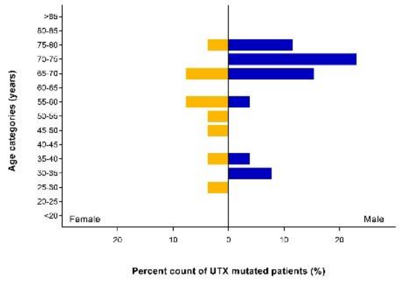

Gene mutations more frequently reported at younger age without sex predominance

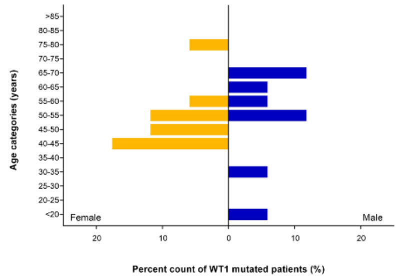

Gene mutations more frequently reported at older age without sex predominance

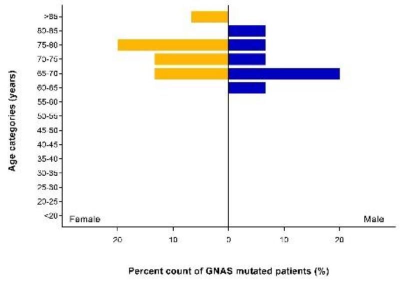

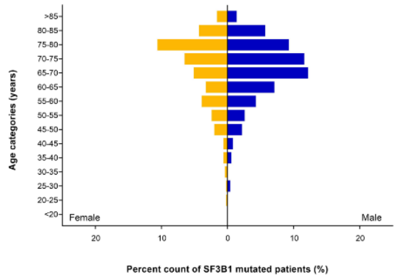

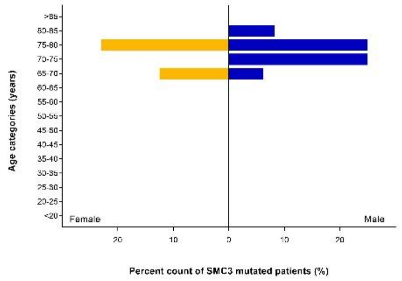

Gene mutations with predominance in men across all age groups

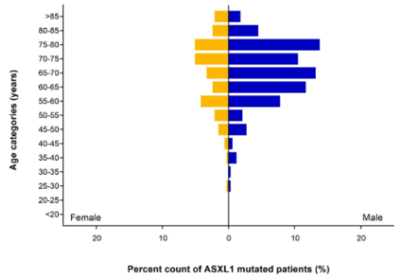

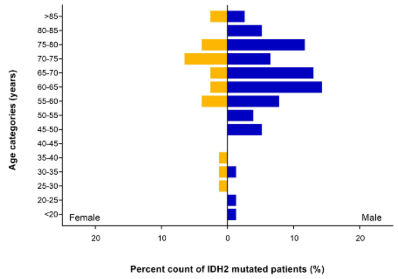

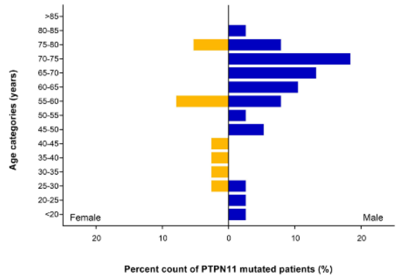

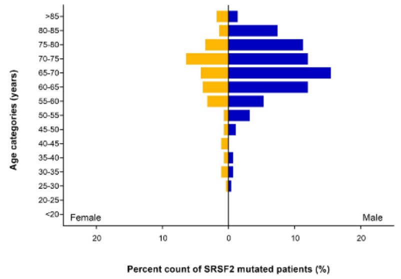

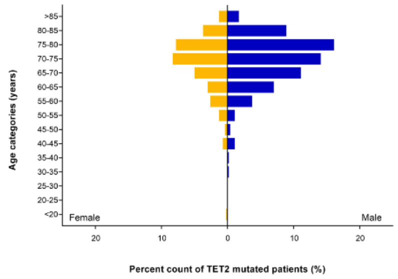

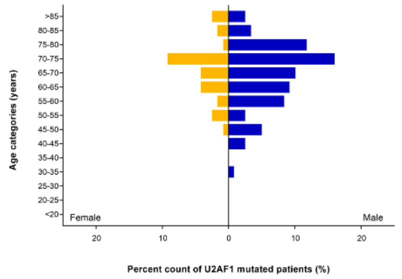

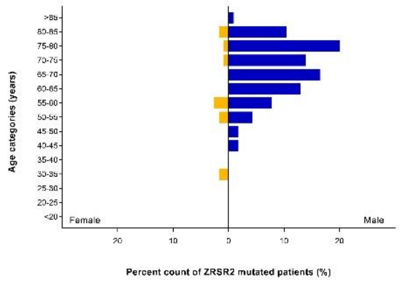

Gene mutations with predominance in women across all age groups

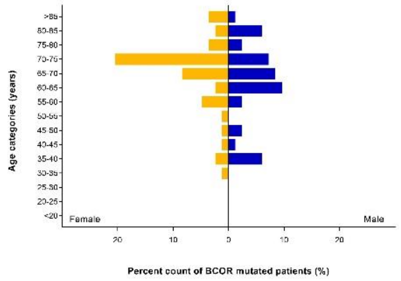

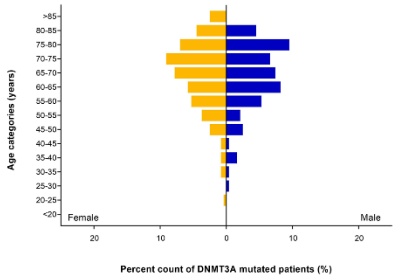

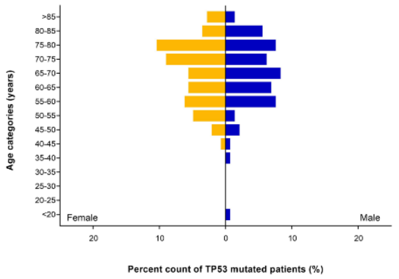

Supplementary Figure_4_SF4. *Distribution of gene mutations in patients from IWG-PM cohort stratified sex across age categories (there are reported only the genes that showed at least 15 mutated patients).*

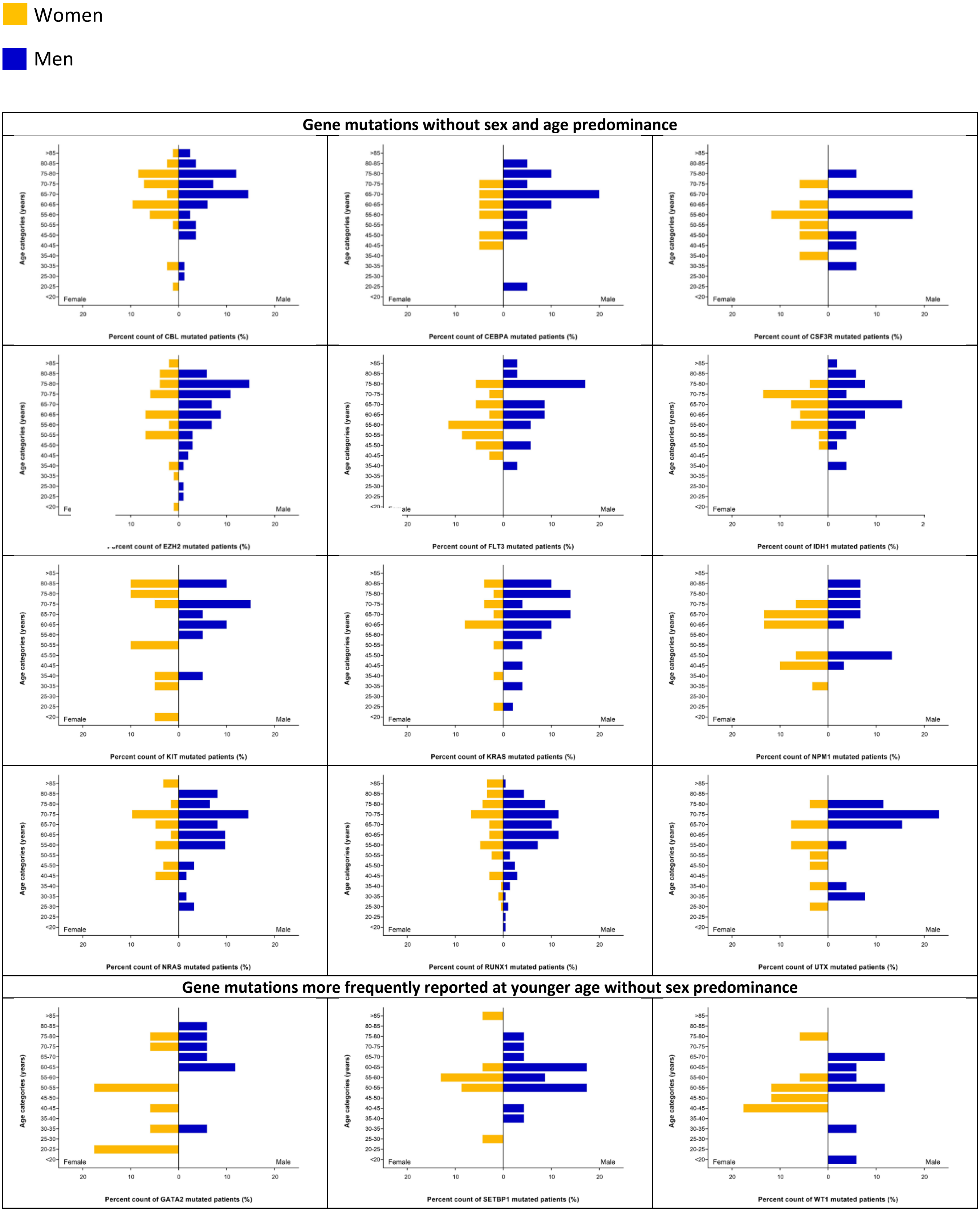

Gene mutations more frequently reported at older age without sex predominance

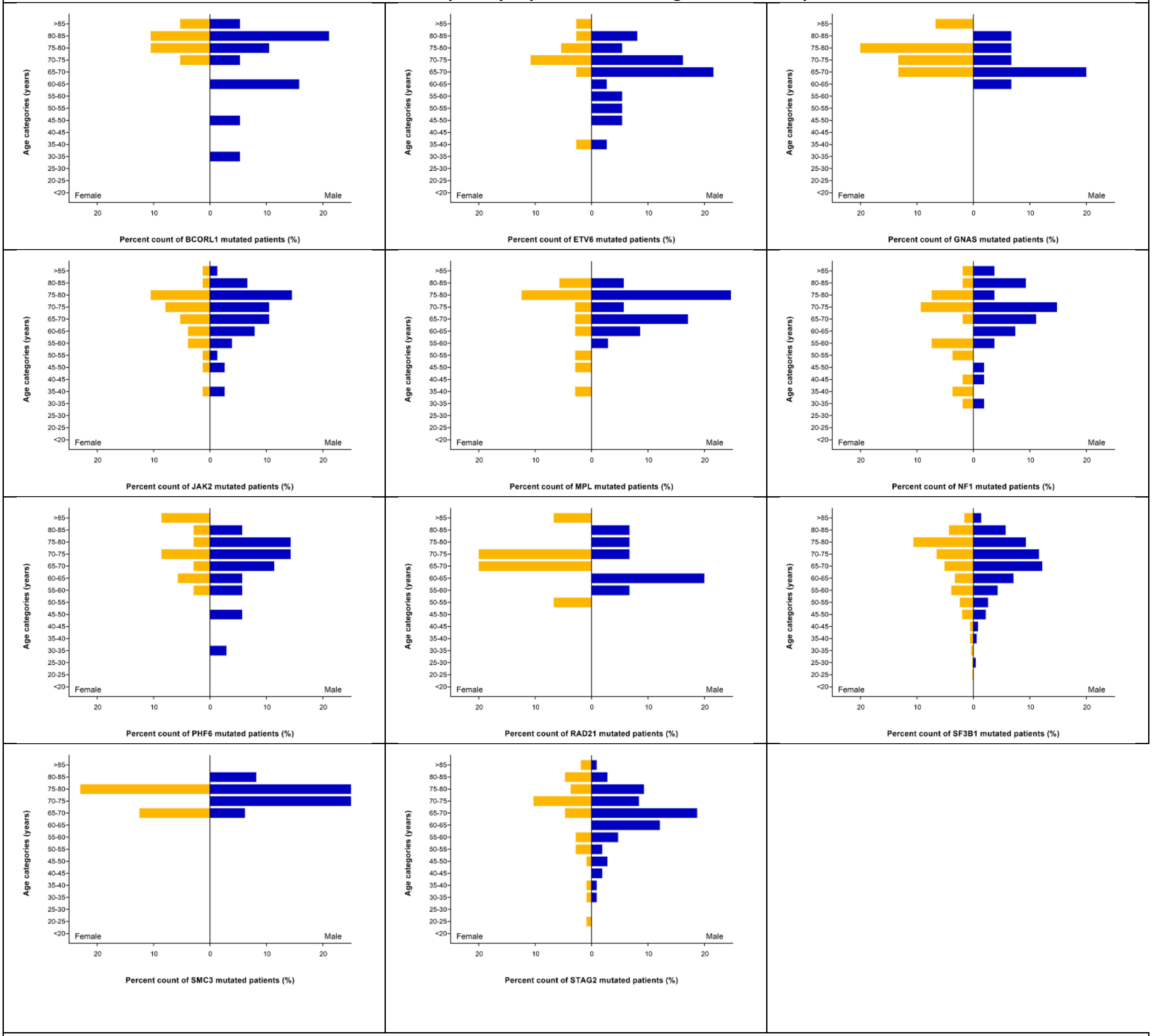

Gene mutations with predominance in men across all age groups

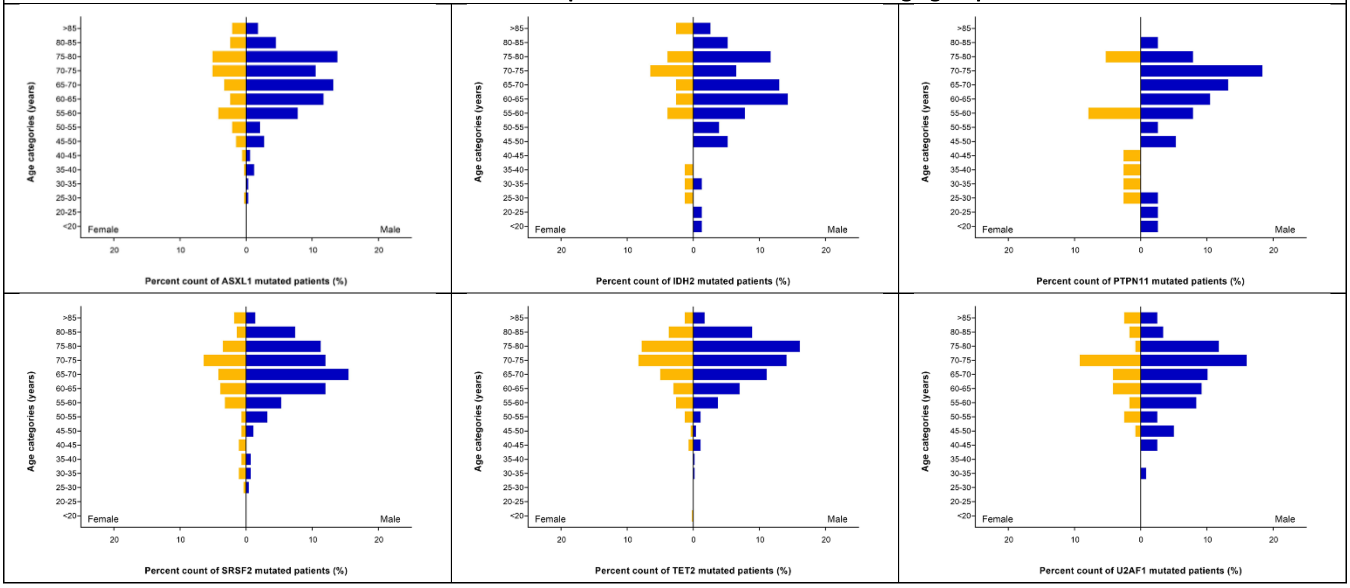

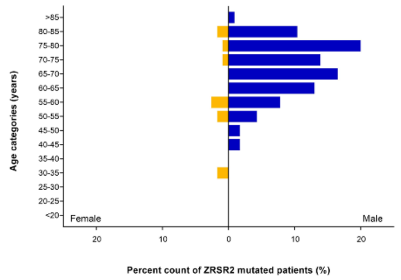

Gene mutations with predominance in women across all age groups

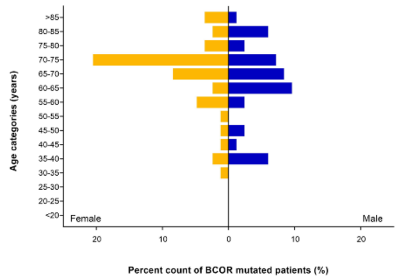

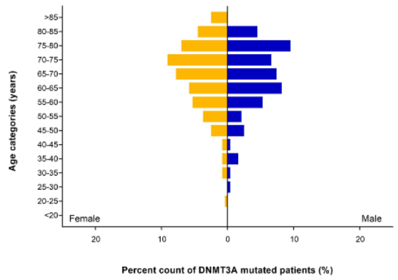

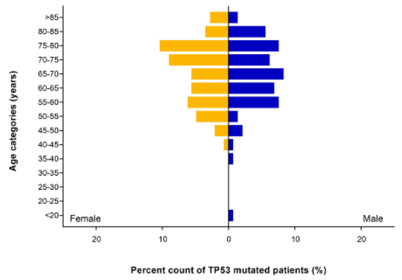

SUPPLEMENTARY_FILE_5 (SF5) - Mutation acquisition order

In order to determine the relative order of mutation acquisition, comparisons were made for each pair of mutations in each patient (for additional details on the methodology, see: https://ascopubs.org/doi/suppl/10.1200/JCO.20.01659). Even without a time course experiment, it is possible to infer the relative order in which two events occurred. Such ordered pairings were used to determine the relative probabilities of a gene mutation occurring first or second for a given pairing with the use of Bradley–Terry (BT) modeling, which provided an estimate of the overall timing of mutation acquisition. For each patient the proportions of cells carrying each mutation, the variant allele fractions corrected for any copy number change at the site of the variant were considered. BT was applied to the set of genes in which genes mutations co-occurring with other gene mutations in at least 15 patients were considered, as it was done in previous works. The data used for the BT model inference were retrieved at site level for 1761 MDS patients from EuroMDS cohort. The R package BradleyTerry2 (version 1.0-8) was used to generate estimates of relative mutation timing. The results for the determination of mutation order in EuroMDS cohort are available in *Supplementary Figure_1_SF6.*

Supplementary Figure_1_SF5. *Determination of MDS mutation order in EuroMDS cohort. The number of pairs n in which the event occurred is shown for each gene on the right of correspondent gene in the plot. Only genes mutations co-occurring with other gene mutations in at least 15 patients were considered. The horizontal axis shows the log odds of a gene occurring second in a gene pair. Any pair of genes can be assessed by calculating the exponential of the difference in log odds for gene A and gene B. Blue asterisks mark statistically significant man-biased genomic abnormalities, and yellow asterisks mark statistically significant woman-biased genomic abnormalities.*

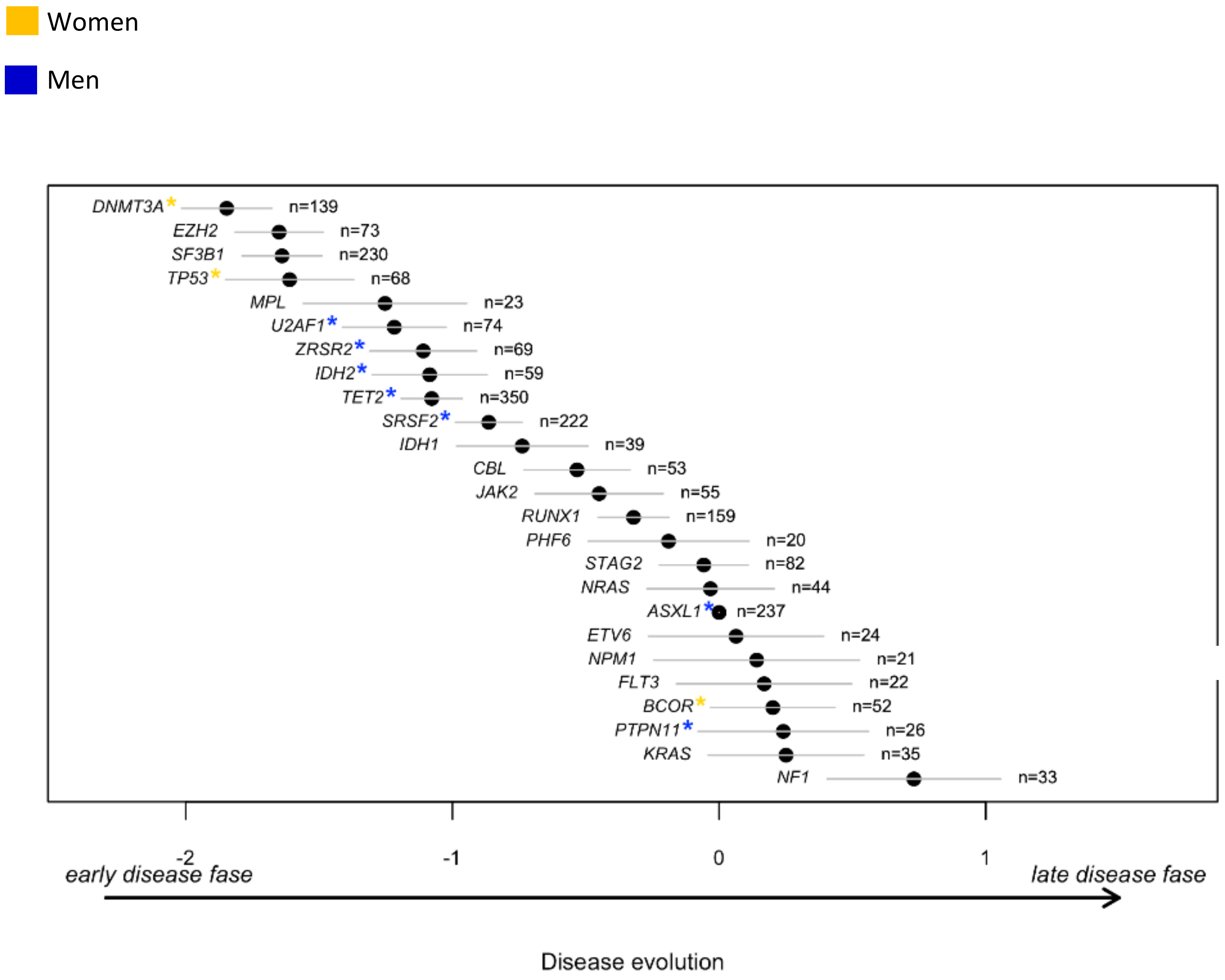

SUPPLEMENTARY_FILE_6 (SF6) - Identification of co-mutational patterns and mutually exclusive mutations in MDS patients stratified by sex

Pairwise associations among genes and cytogenetic abnormalities in MDS patients stratified by sex

In order to assess pairwise association among genes and/or cytogenetics abnormalities, we calculated the co-occurrence of genomics abnormalities across patients. In more details, for each couple of genomic abnormalities, the number of patients showing mutation co-occurrence were quantified. 2x2 contingency tables were generated by each pair present and the significance was evaluated with Fisher's exact test. Furthermore, for each possible pairing of genes and/or cytogenetic abnormalities the odds ratio was calculated. Odds ratios less than 1 indicates that the pairs of mutation were mutually exclusion, while odds ratios greater than 1 implies mutation co-occurrence.

Supplementary Figure_1_SF6. *Pairwise associations among genes that happen to be mutated in at least 40 patients and cytogenetic abnormalities in 2025 MDS patients from EuroMDS cohort, stratified by sex. In the upper triangle, for each couple of genomic abnormalities, the number of patients showing mutation co-occurrences are illustrated using a blue color scale. In the lower triangle the gene-gene co- occurrence and mutual exclusivity is assessed using odds ratio and significance is evaluated using Fisher test. Multiple hypothesis testing was performed using the Benjamini-Hochberg adjustment in order to control the false discovery rate, meaning the expected proportion of false discoveries amongst the rejected hypotheses. Such a correction is necessary when dealing with such a high number of comparisons and help to identify the most significant associations.*

A. Men from EuroMDS cohort

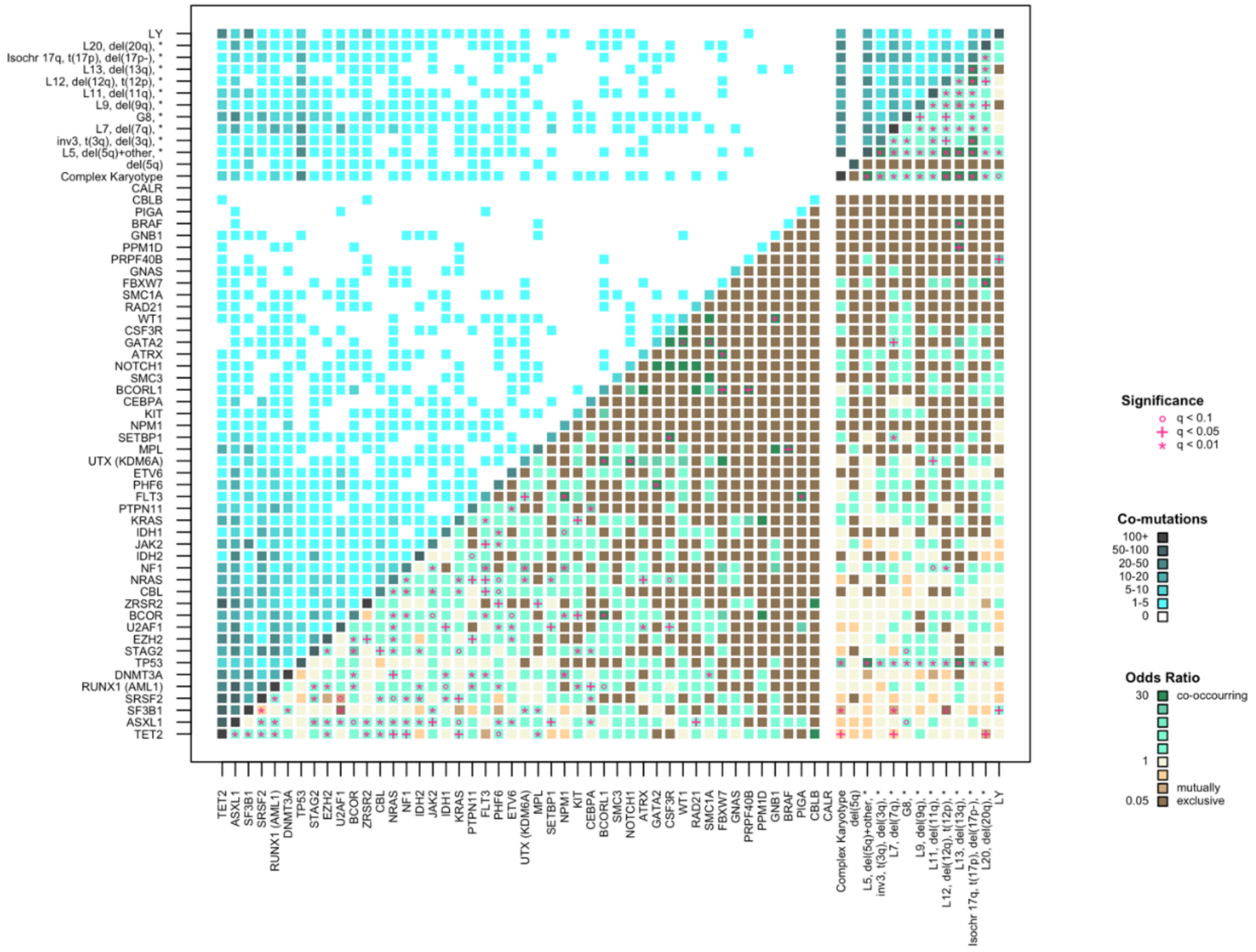

B. Women form EuroMDS cohort

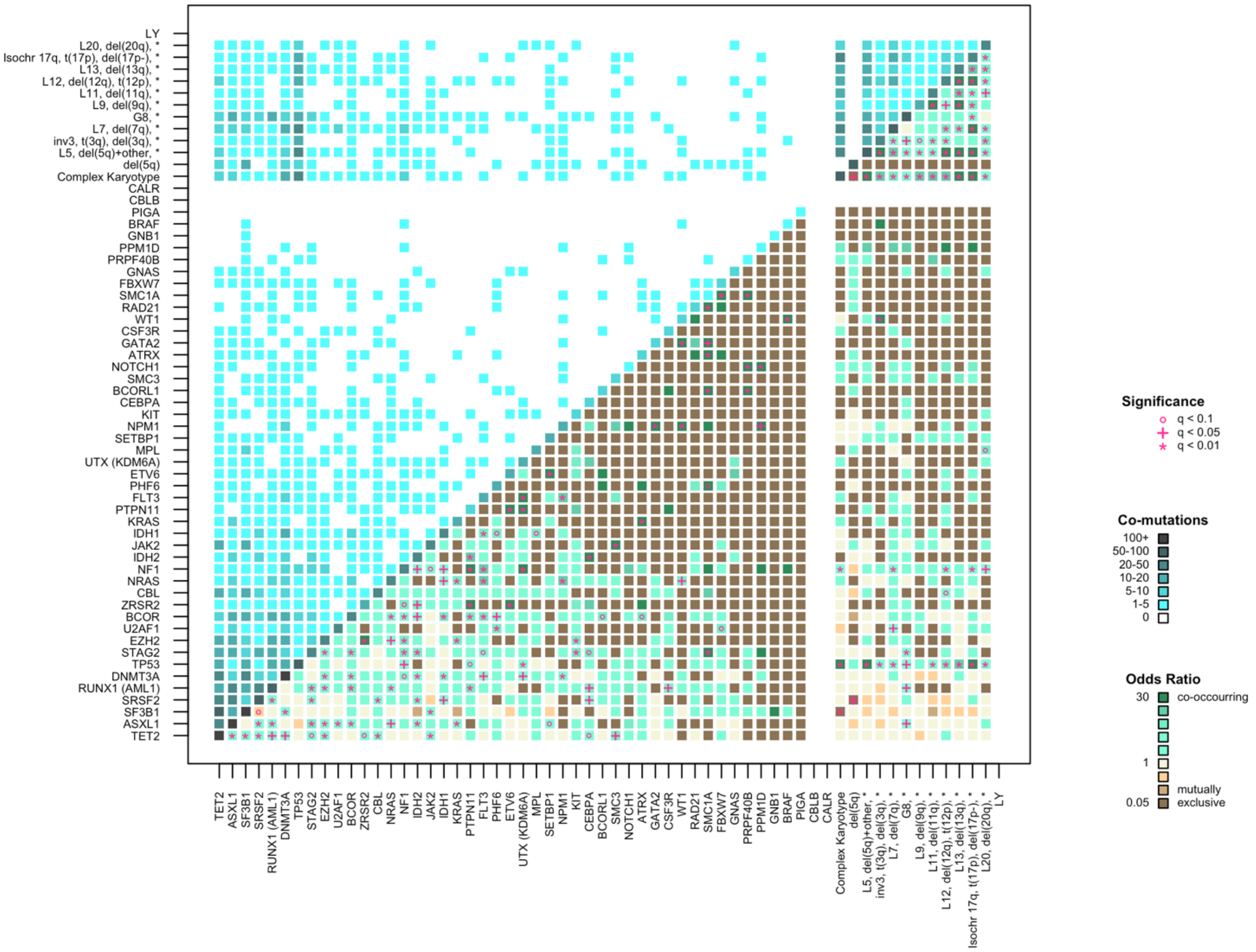

Supplementary Figure_2_SF6. *Pairwise associations among genes that happen to be mutated in at least 40 patients and cytogenetic abnormalities in 2,387 MDS patients from IWG-PM cohort, stratified by sex. In the upper triangle, for each couple of genomic abnormalities, the number of patients showing mutation co-occurrences are illustrated using a blue color scale. In the lower triangle the gene-gene co- occurrence and mutual exclusivity is assessed using odds ratio and significance is evaluated using Fisher test. Multiple hypothesis testing was performed using the Benjamini-Hochberg adjustment in order to control the false discovery rate, meaning the expected proportion of false discoveries amongst the rejected hypotheses. Such a correction is necessary when dealing with such a high number of comparisons and help to identify the most significant associations.*

A. Men from IWG-PM cohort

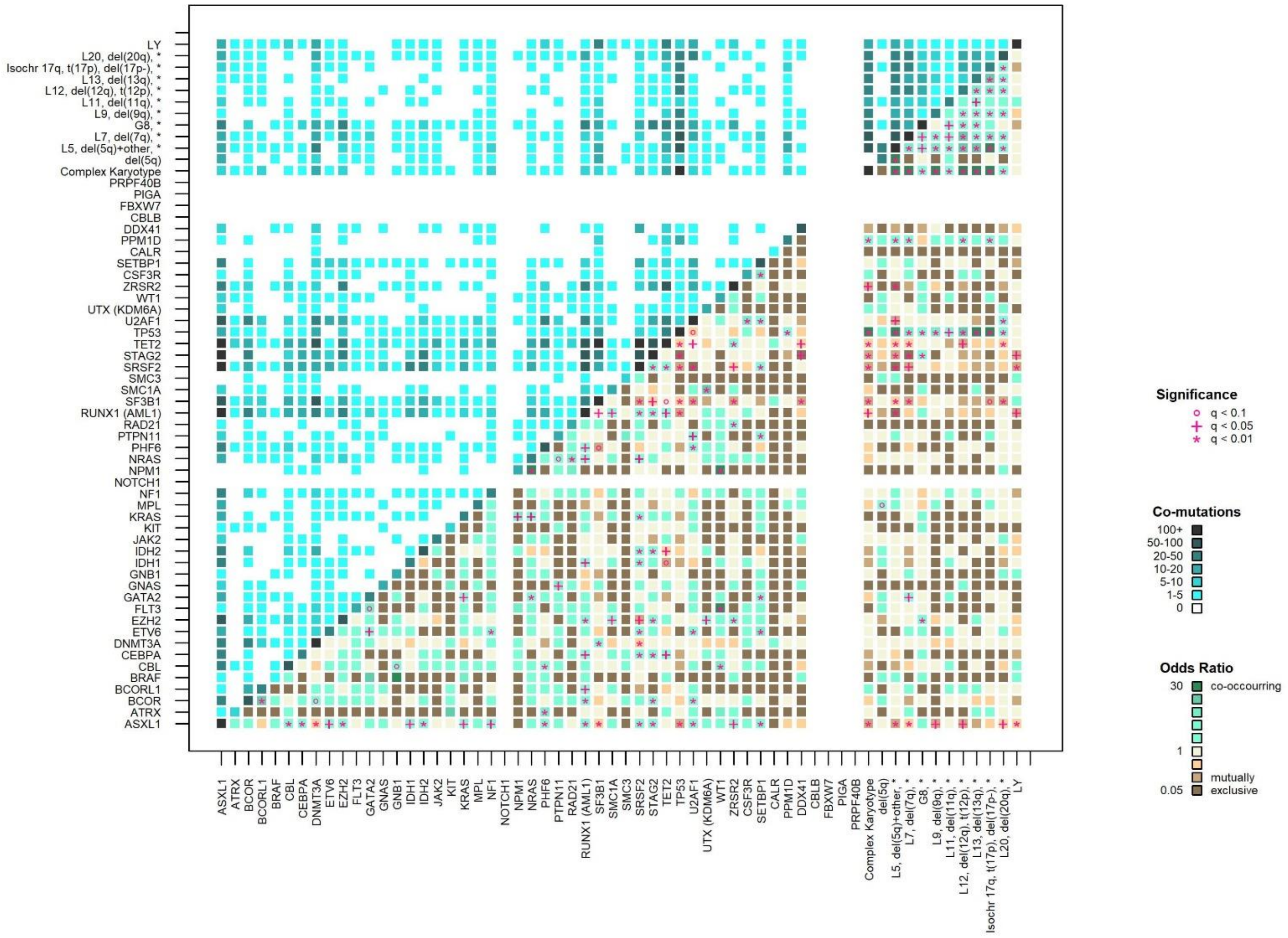

B Women from IWG-PM cohort

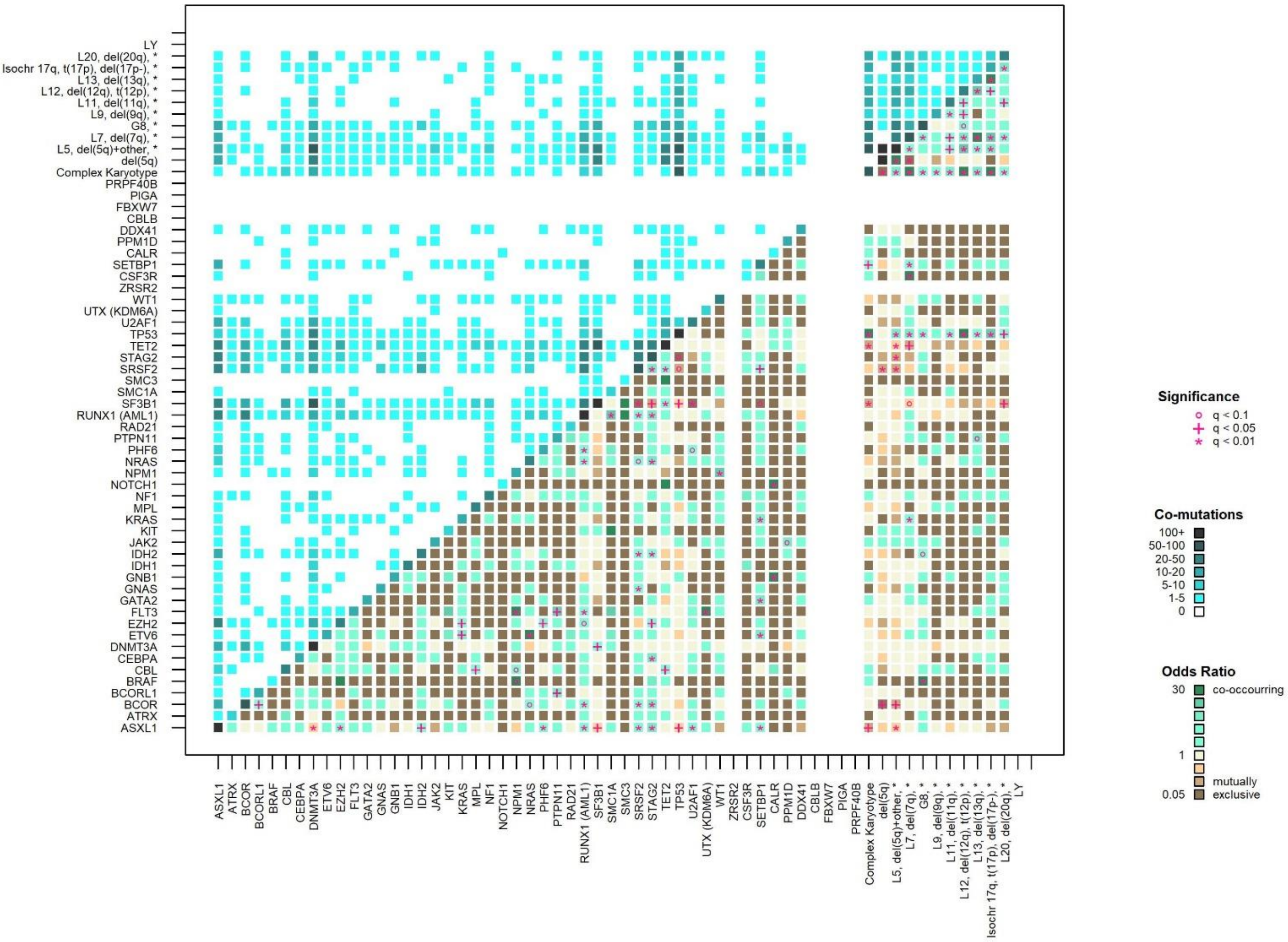

Bayesian networks

We used Bayesian Networks (BN) to define in a more comprehensive way the relationships between genomic abnormalities in MDS stratified by sex. We included gene mutations and cytogenetic abnormalities as random variables in the model and we investigated conditional dependency among them.

Given a set of variables (in our study the set of cytogenetic and genetic mutations), a BN is a graphical way to highlight conditional dependencies among variables, i.e. how the values taken by a given variable influences the probability of the others. They main hypothesis underlying BN is that joint probability distribution (JPD) over the set of variables could be represented as a Directed Acyclic Graph (DAG), i.e. a directed graph with no loops.
DAG nodes represent random variables; each node i is associated with the probability distribution P_i, the probability of observing a mutation at the i-th position; while a link represents a dependence among two variables (i.e. how the presence of a given mutation influences the presence of the other). For instance, an arrow from node A to node B is a probabilistic direct dependence between A and B. Directed dependence means that the value taken by B is influenced by the value taken by A while the vice versa in not true, i.e. we have a causal connection between the variables.
More formally, given the set of variables x = (x_1,... ,x_n), BNs are a way to represent a specific factorization of their JPF. Given the DAG structure (S), the joint probability distribution is given by:

$$P(x_1, \dots, x_n) = \prod_{i=1}^{n} P_i(x_i | Parents_i^S)$$

where the factorized probabilities are conditioned on the parents of the node i in the directed acyclic graph (S). This is equivalent to say that each variable is independent of its non-child nodes in the graph given the state of its parents. Given the training data we estimated the network structure (S) and the parameters of the JPD in the BN (i.e. P_i for i = 1,...,n). We inferred the network structure from data using the GOBNILP software:[4] given a set of random variables, GOBNILP assigns a score (based on data) to each Directed Acyclic Graph and choose the structure which maximizes the score (according to previous literature[1,3] we set the maximum number of parents to 3). For each variable in which conditional dependency was found (i.e. a link in the inferred structure is present), the definition of mutually exclusivity was used to define a significant negative dependency, while the definition of co-occurrence was used to define a positive dependency.

For additional details on the methodology, please see: https://ascopubs.org/doi/suppl/10.1200/JCO.20.01659

Supplementary Figure_3_SF6. *Genomic Landscape of MDS through Bayesian Networks in 2,025 patients from EuroMDS cohort, stratified by sex. Given a set of variables (in our study the set of cytogenetic and genetic mutations), a Bayesian Networks is a graphical way to highlight conditional dependencies among variables, i.e. how the values taken by a given variable influences the probability of the others. The size of each node accounts for the number of correspondent genomic or cytogenetic alterations. The color of each link reflects odds ratio of co- occurrence or mutually exclusivity as calculated previously in Figure_1_SF6. The thickness of edges grows with increasing significance of mutual exclusivity / co-occurrence between alterations.*

A. Men from EuroMDS cohort

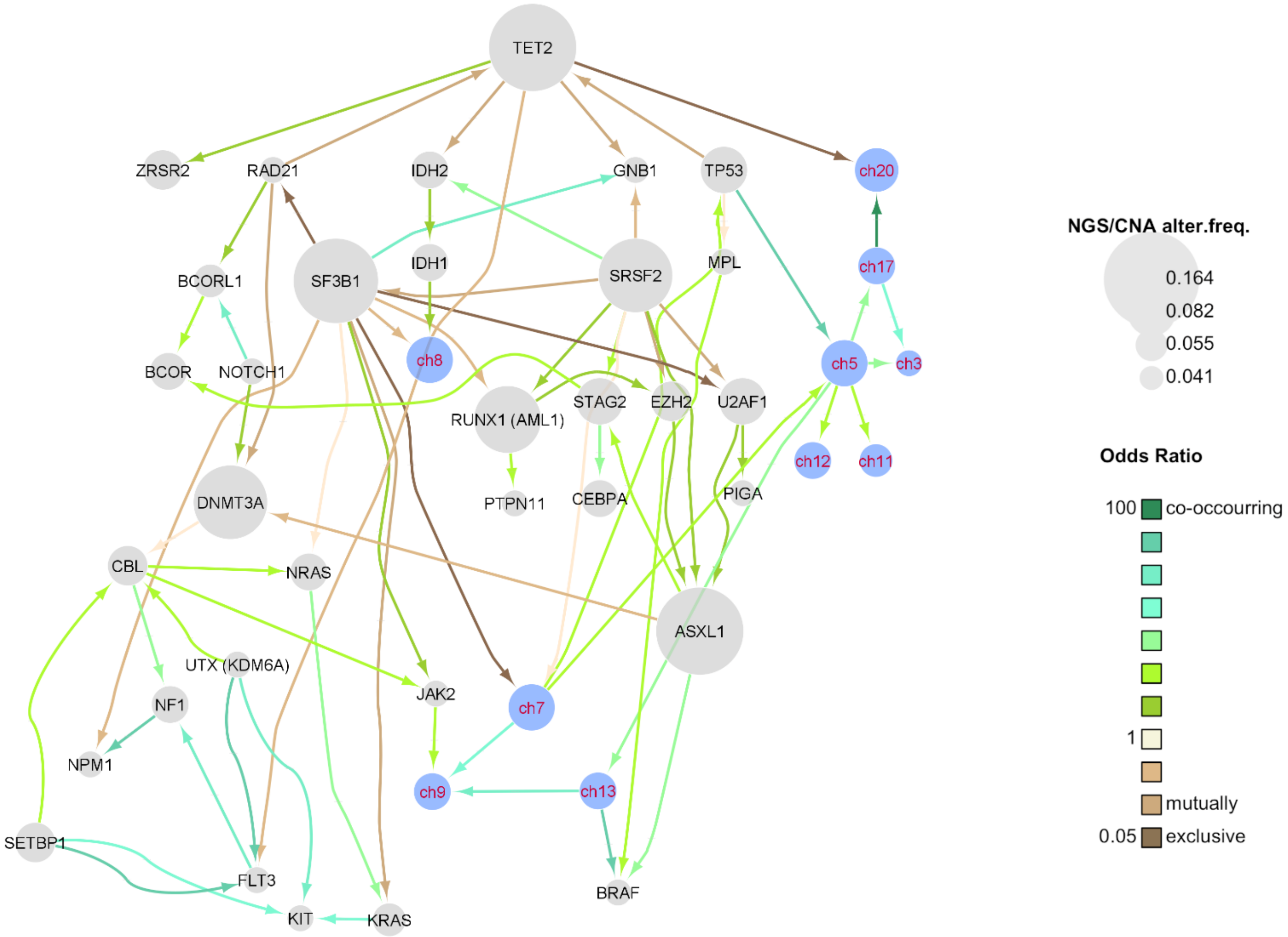

B. Women from EuroMDS cohort

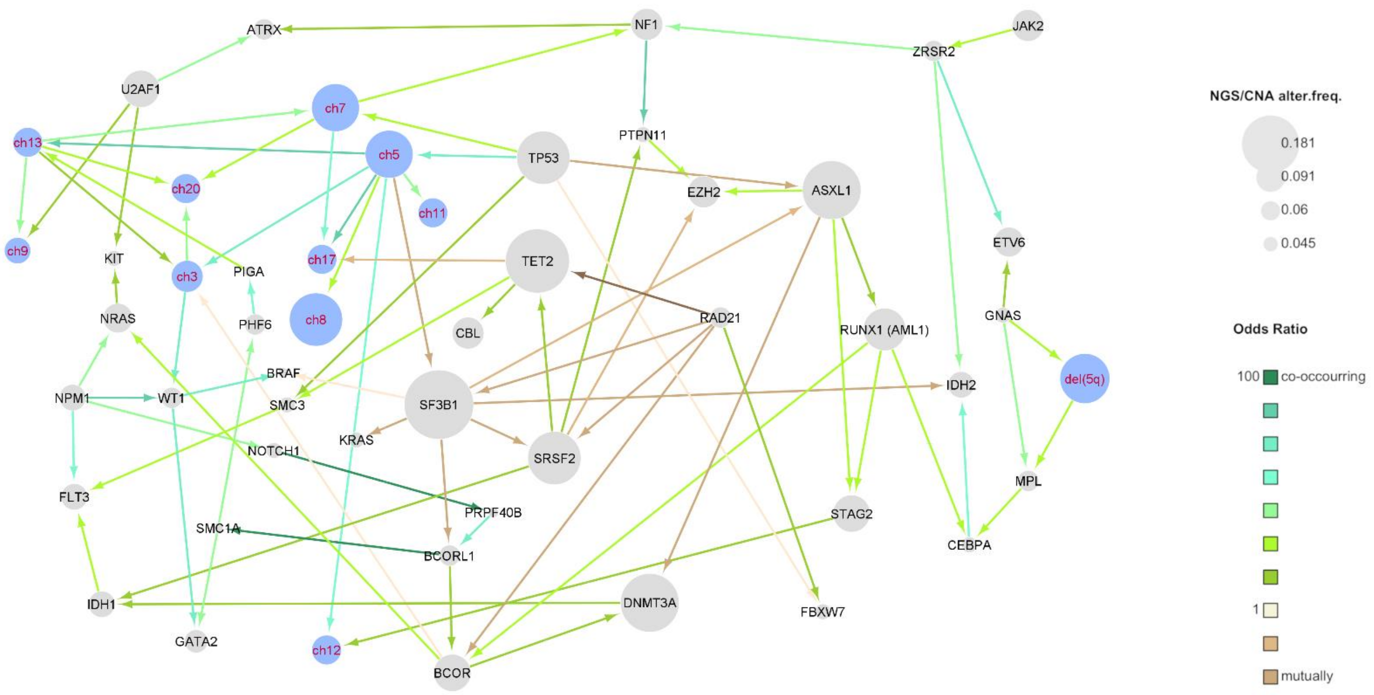

Supplementary Figure_4_SF6. *Genomic Landscape of MDS through Bayesian Networks in 2,387 patients from IWG-PM cohort, stratified by sex. Given a set of variables (in our study the set of cytogenetic and genetic mutations), a Bayesian Networks is a graphical way to highlight conditional dependencies among variables, i.e. how the values taken by a given variable influences the probability of the others. The size of each node accounts for the number of correspondent genomic or cytogenetic alterations. The color of each link reflects odds ratio of co- occurrence or mutually exclusivity as calculated previously in Figure_2_SF6. The thickness of edges grows with increasing significance of mutual exclusivity / co-occurrence between alterations.*

A. Men from IWG-PM cohort

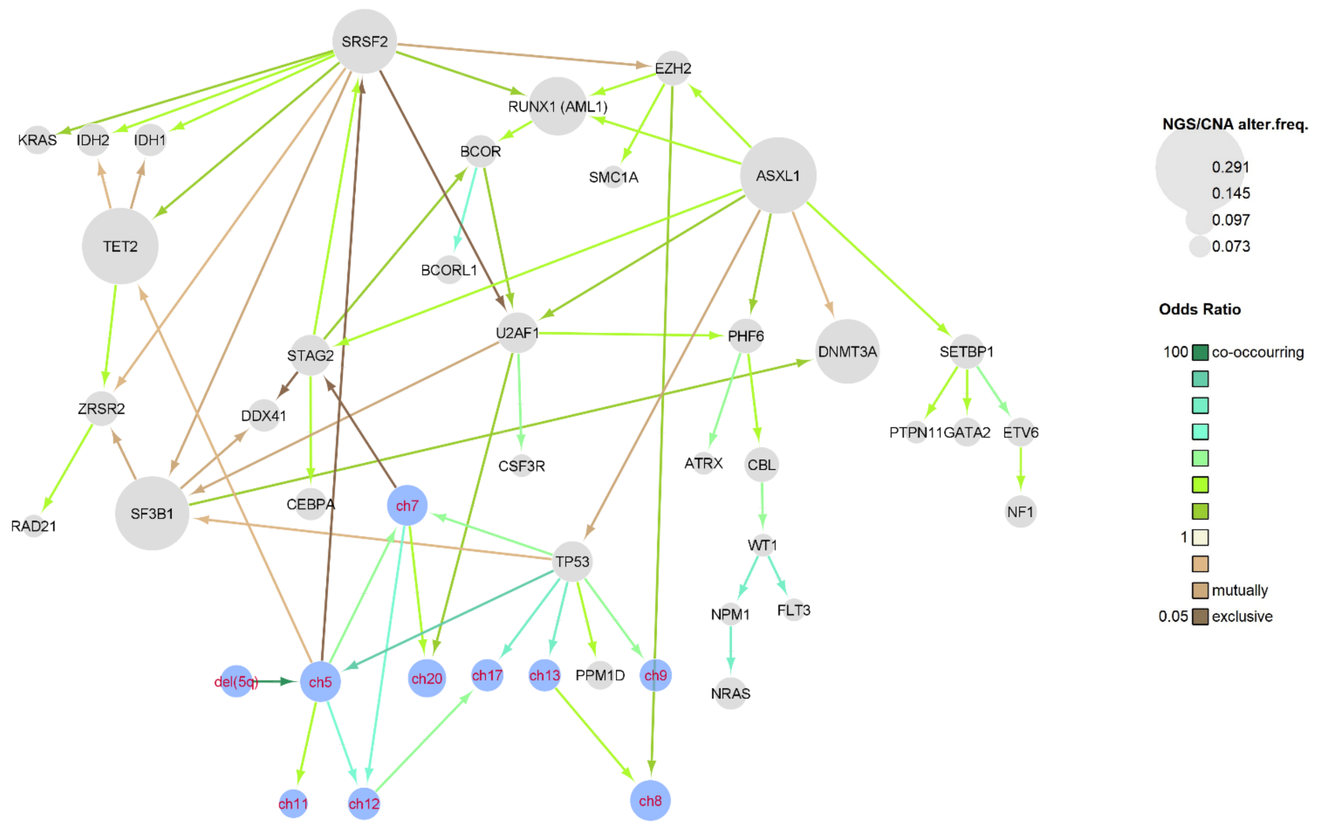

B. Women from IWG-PM cohort

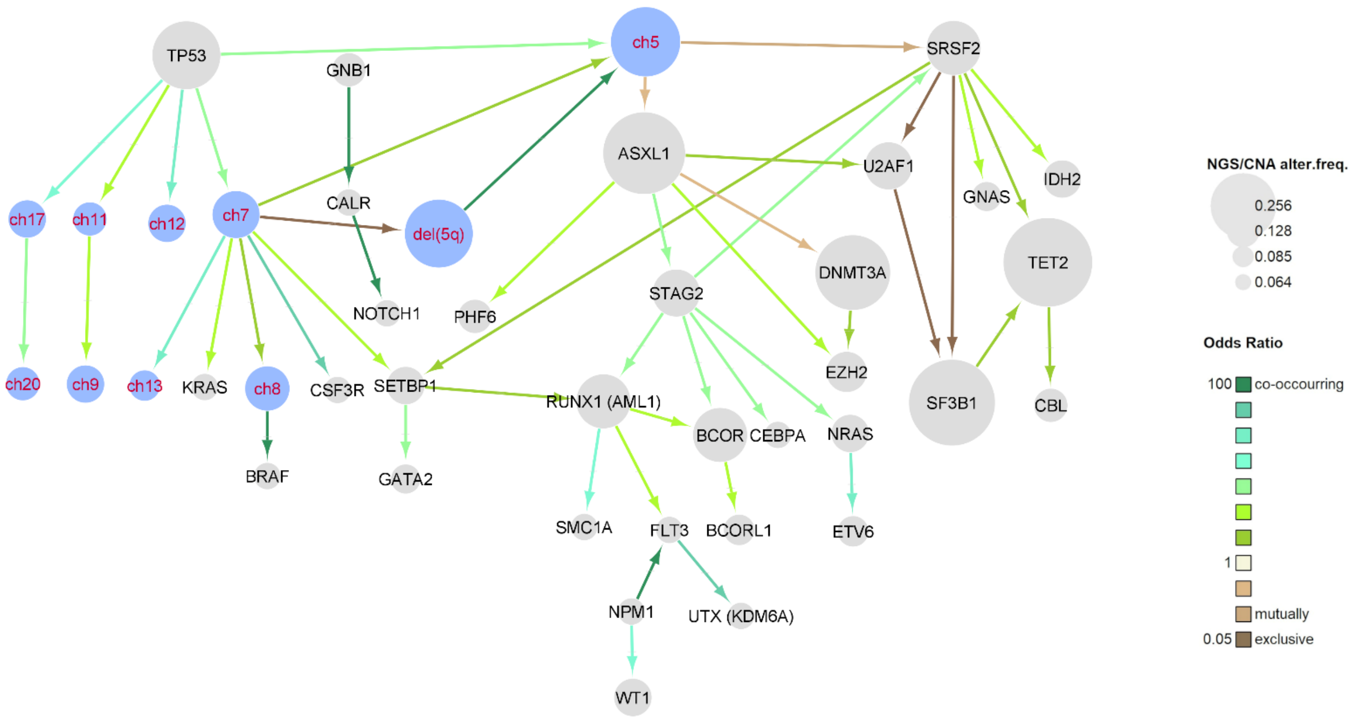

Supplementary Table_1_SF6. *Description of statistically significant (P value <0.05) co-occurring and exclusive mutations/chromosomal abnormalities (representative genes and chromosomal abnormalities are showed) in men vs women from EuroMDS and IWG-PM cohorts (only significant relationships in both populations are reported).*

Gene	Mutation	Men	Women
ASXL1	Exclusive	*DNMT3A*	*TP53, SF3B1, DNMT3A*
	Co-occurring	*BRAF, SRSF2, STAG2, EZH2*	*EZH2, RUNX1, STAG2*
DNMT3A	Exclusive	*ASXL1, RAD21*	*ASXL1*
	Co-occurring	*SF3B1*	*IDH1, BCOR*
TET2	Exclusive	*TP53, GNB1, IDH2, FLT3, Cr20*	*RAD21,*
	Co-occurring	*ZRSR2*	*SRSF2,CBL, EZH2*
SRSF2	Exclusive	*GNB1, EXH2, SF3B1, U2AF1*	*SF3B1, RAD21, EZH2*
	Co-occurring	*ASXL1, RUNX1, STAG2, IDH2*	*TET2, IDH1*
SF3B1	Exclusive	*KRAS, NPM1, SRSF2, RAD21, U2AF1, RUNX1,* Gain of chr 8, Loss of chr 7 or del(7q)	*BCORL1, IDH2, KRAS, RAD21, ASXL1,* Loss of chr 5 or del(5q) with other abnormalities
	Co-occurring	*GNB1, JAK2*	--
U2AF1	Exclusive	*SRSF2, SF3B1*	--
	Co-occurring	*ASXL1*	*ATRX, KIT*
ZRSR2	Exclusive	---	---
	Co-occurring	*TET2*	*JAK2, ETV6, IDH2, NF1*
TP53	Exclusive	*TET2*	*ASXL1*
	Co-occurring	Loss of chr 5 or del(5q) with other abnormalities, Loss of chr 7 or del(7q)	Loss of chr 5 or del(5q) with other abnormalities, Loss of chr 7 or del(7q)

Supplementary Figure_5_SF6 *Genomic Landscape of MDS through Bayesian Networks. Comparison between EuroMDS and IWG-PM cohorts inferred relations. All the represented relations are significantly determined in EuroMDS cohort and are confirmed in IWG-PM cohort.*

Women

Men

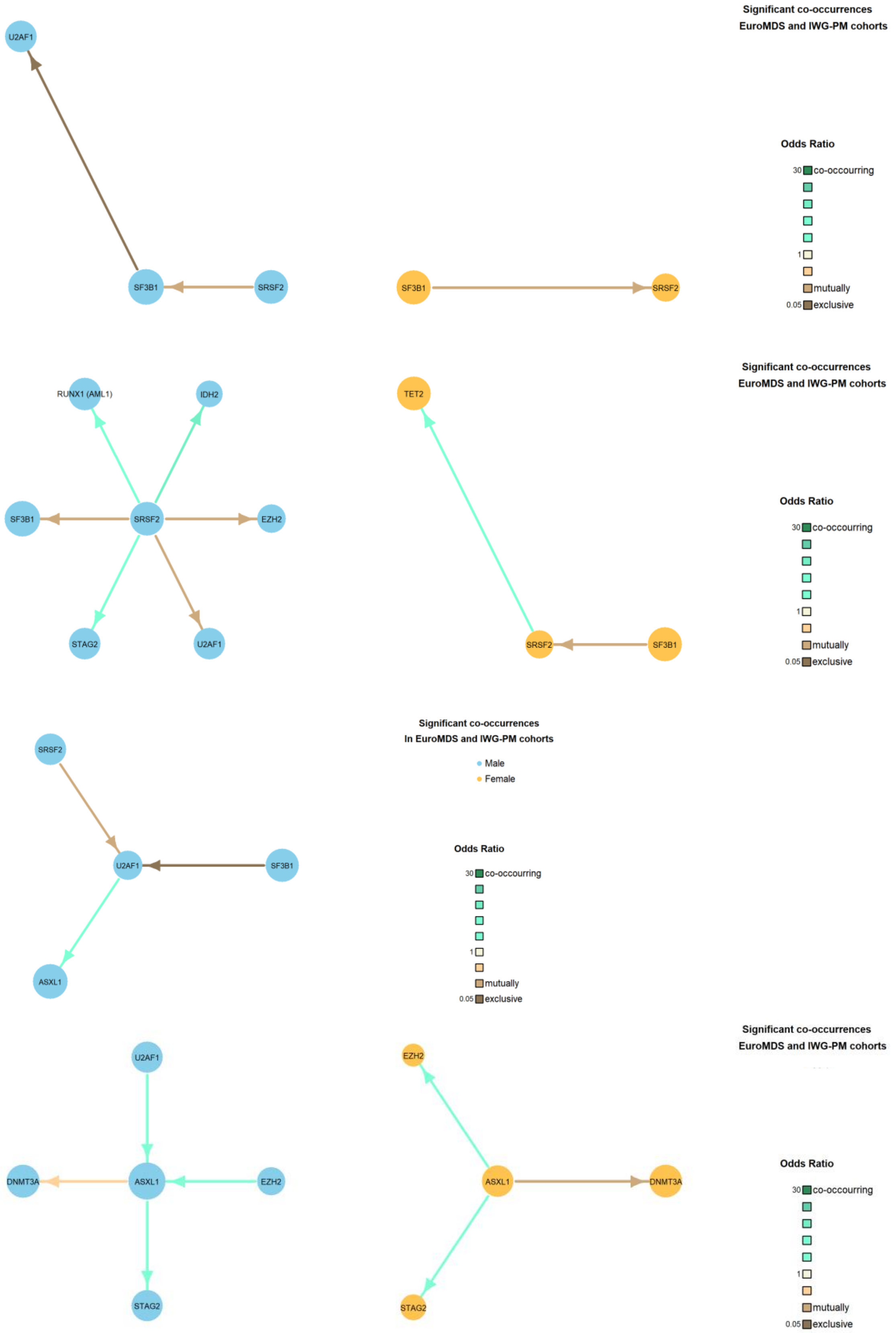

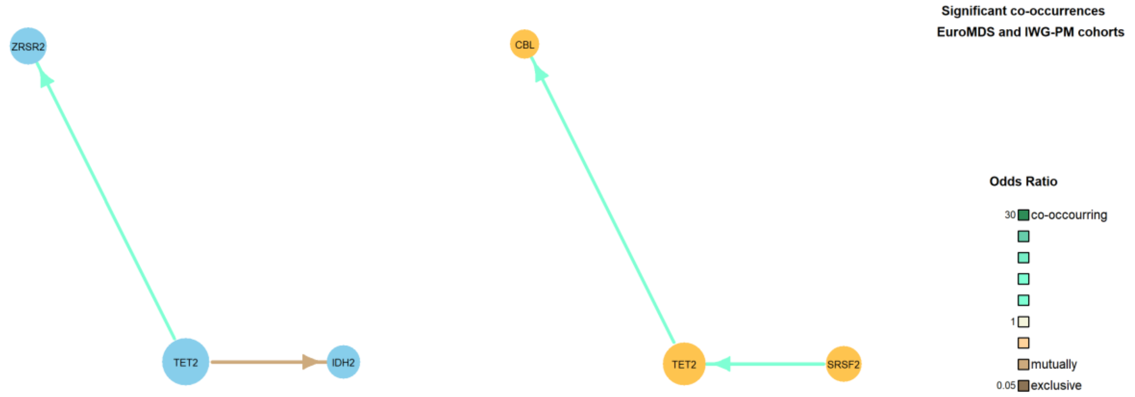
ZRSR2
TET2
IDH2
CBL
TET2
SRSF2
Significant co-occurrences
EuroMDS and IWG-PM cohorts
Odds Ratio
30
co-occourring
1
mutually
0.05
exclusive

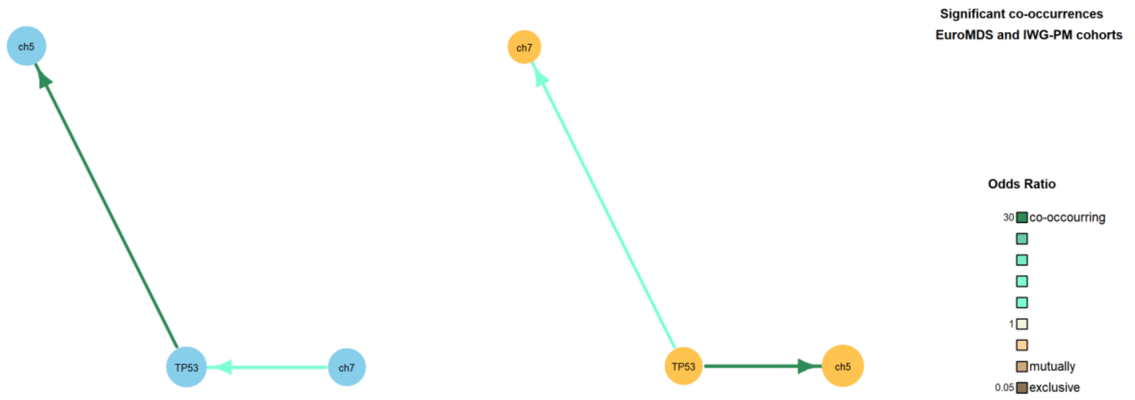
ch5
TP53
ch7
ch7
TP53
ch5
Significant co-occurrences
EuroMDS and IWG-PM cohorts
Odds Ratio
30
co-occourring
1
mutually
0.05
exclusive

Dirichelet Processes Multinomial Mixture Model

In order to identify MDS molecular subtypes we carried out Dirichelet Process Clustering (DP). The DP infinite multinomial mixture model allows to capture broad dependencies among all gene mutations assuming them to be extracted from a mixture of multinomials. The rationale underlying the model is that we expect mutations to be clustered together according to the specific molecular mechanism at work in a given tumor. Using an infinite mixture with DP prior, instead of finite mixture, allows not to specifying a priori the number of mutations categories, which, instead, is inferred from the data. Importantly, the usage of advanced clustering methods such as DP for patient clustering allows to avoid overfitting issues. To carry out the analysis we used the R package HDP available online https://github.com/nicolaroberts/hdp).
The input data consists of a patient by genes binary matrix. The genotype of a patient is a row of the matrix: G=(G_1,...,G_n); where n is the number of features per patient (in our case: 12 cytogenetic and 47 genomic variables). G_{ij} is a binary variable which denotes the presence or absence of i-th alteration. Missing data where imputed with R package copynumber. The analysis was performed using different kinds of imputation with comparable results. Patients with no alterations were excluded from the DP clustering and classified as a class on their own.
More formally, DP mixture model assumes data to be generate according to the following process:

- $\theta \sim DP(Dirichlet(\alpha), \alpha_0)$
- $X \mid \theta, N \sim Multinomial(\theta, N_j)$

where: θ are the parameters of the multinomials, α_0 is the concentration parameter of the DP process and α are the parameters of the base distribution with parameter $\alpha = (1/n,...,1/n)$.
We carried out Monte Carlo Markov Chain (MCMC) sampling of DP posterior for 4 different initial conditions (n. of different chains). For each chain we discarded the first 3000 iterations and we sampled 4000 realizations at intervals of 20 iterations. Components are built by grouping raw clusters of DP posterior samples according to the following conditions: 1) clusters are merged if their cosine similarity is above a give threshold (0.95 in our case) and 2) clusters are assigned to component 0 if they have no significant data categories or sample exposure. Components 1-5 account for the 97% of the data while component 0 accounts for data that cannot be explained by the model.
The model found a mixture of multinomials with 5 components, plus an additional one of unexplained data.

For additional details on the methodology, please see: https://ascopubs.org/doi/suppl/10.1200/JCO.20.01659

Supplementary Figure_6_SF6 *Distribution of MDS genomic-based groups in patients from EuroMDS (A) and IWG-PM cohorts (B), stratified by sex. (MDS genomic-based groups were defined according to Bersanelli M, et al. Classification and Personalized Prognostic Assessment on the Basis of Clinical and Genomic Features in Myelodysplastic Syndromes. J Clin Oncol 39: 1223-1233, 2021)*

Women

Men

A)

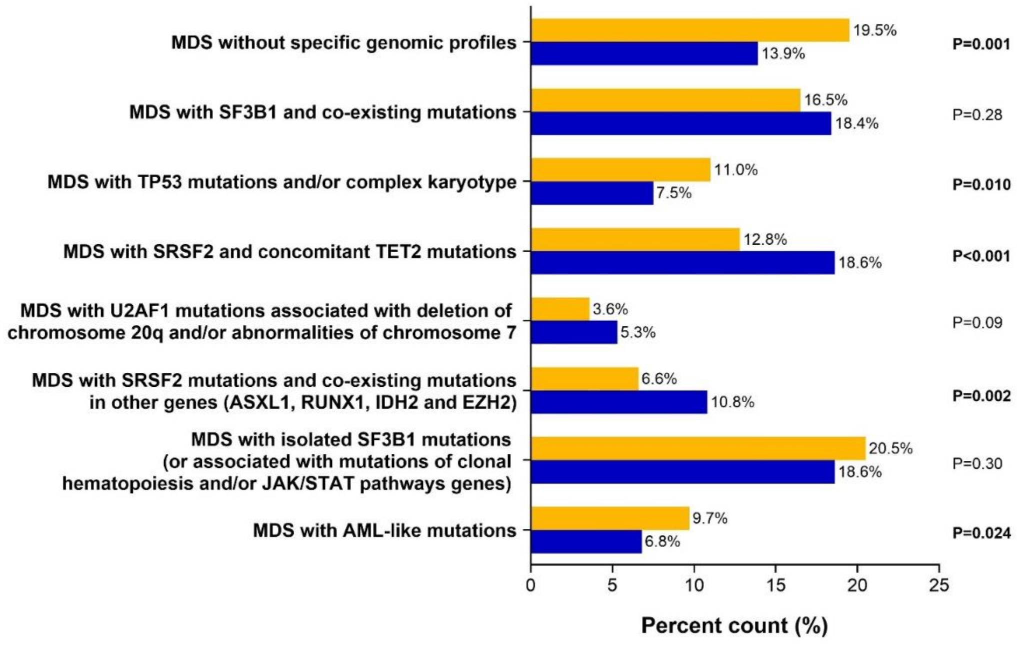

B)

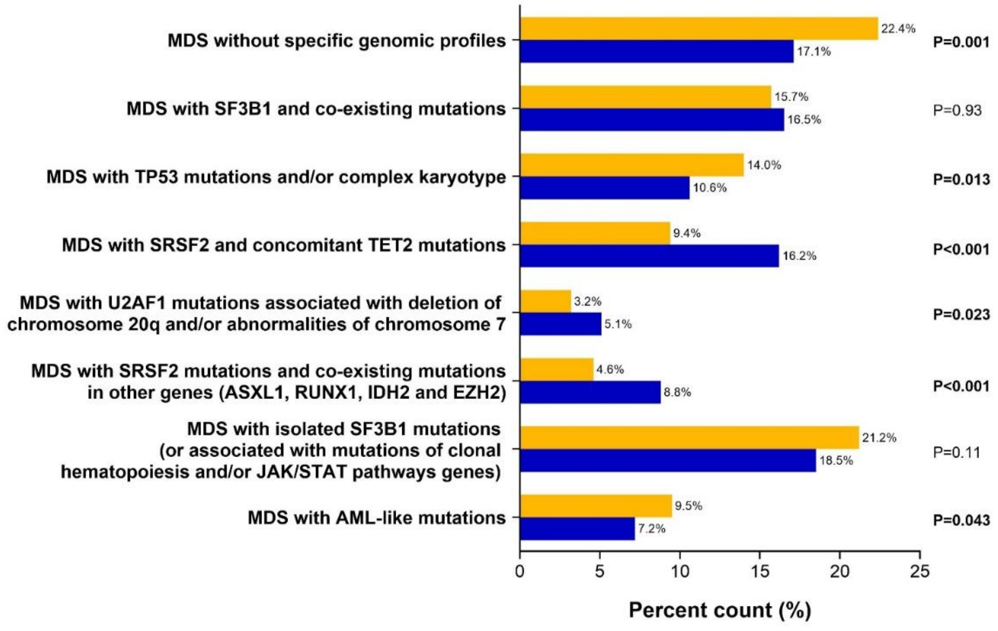

SUPPLEMENTARY_FILE_7 (SF7) - Sex effect on MDS clinical outcome

Supplementary Figure_1_SF7. *Probability of overall survival of MDS patients belonging to retrospective EuroMDS cohort (plot A), retrospective IWG-PM cohort (B), prospective registry of Spanish MDS Group (GESMD) (C) and prospective Düsseldorf MDS registry (D).*

Women

Men

A. Probability of overall survival of EuroMDS cohort stratified by sex (men vs women HR 1·40, CI 1·26-1·52, P<0·0001)

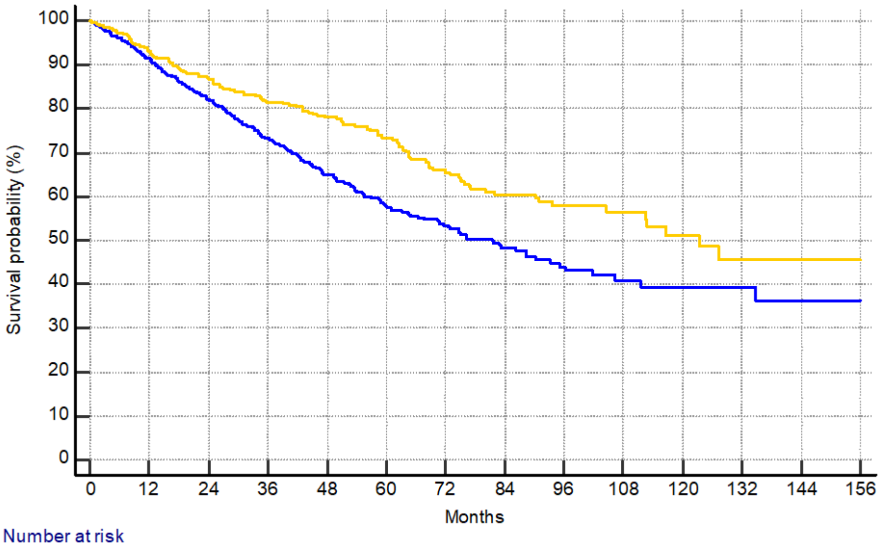

Number at risk

	0	12	24	36	48	60	72	84	96	108	120	132	144	156
Group: Male	1205	700	501	385	278	196	139	84	51	31	20	13	11	8
Group: Female	820	478	370	294	226	176	127	90	60	37	22	13	7	6

B. Probability of overall survival of IWG-PM cohort stratified by sex (men vs women HR 1·33, CI 1·13-1·57, P<0·0001)

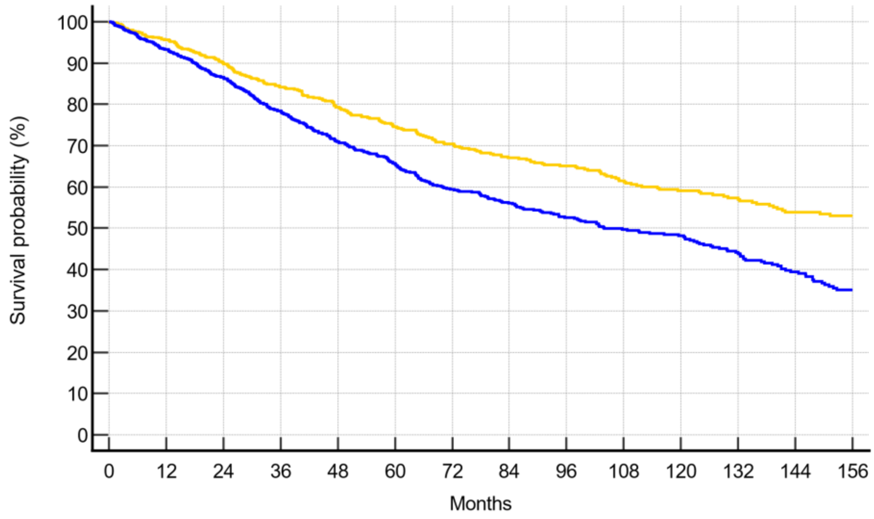

Number at risk

	0	12	24	36	48	60	72	84	96	108	120	132	144	156
Group: F	874	664	552	470	415	369	325	281	253	213	184	156	135	121
Group: M	1331	919	746	611	494	419	348	306	255	210	176	139	108	81

C. Probability of overall survival of Spanish MDS Group (GESMD) cohort stratified by sex (men vs women HR 1·30, CI 1·24-1·35, P<0·0001)

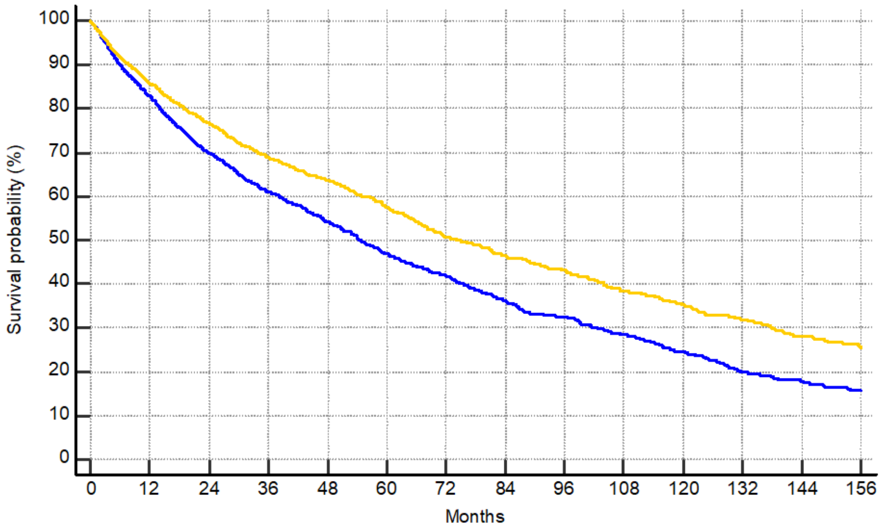

Number at risk

Group: Male	4415	2723	1885	1341	1007	748	568	405	317	234	170	115	92	64
Group: Female	3263	2200	1646	1257	1005	792	611	468	380	302	236	170	122	96

D. Probability of overall survival of Düsseldorf MDS registry cohort stratified by sex (men vs women HR 1·23, CI 1·07-1·36, P=0·0061)

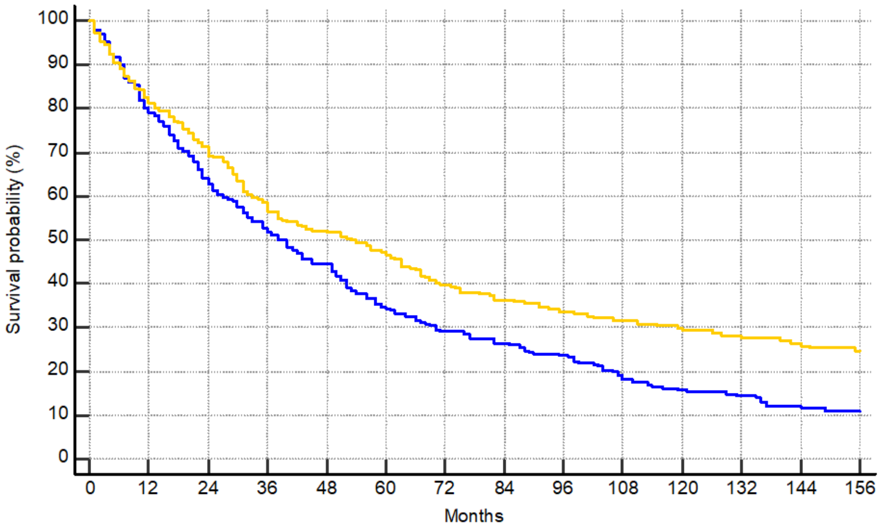

Number at risk

Group: Male	725	440	309	232	179	122	93	76	65	45	38	32	22	19
Group: Female	459	290	219	165	144	118	95	80	66	60	54	48	42	38

Supplementary Figure_2_SF7. *Competing risk analysis of leukemic death (LD) vs non leukemic death (NLD) in MDS patients from EuroMDS cohort with early disease stage (defined by IPSS-R score ≤3·5), stratified by sex. When estimating the occurrence of non-leukemic death, only deaths for all causes except leukemic evolution were considered as events. * The 5-year risk of non-leukemic death was 32·1% in men vs 18.4% in women (P<0·0001), while no difference was found regarding the risk of leukemic death*

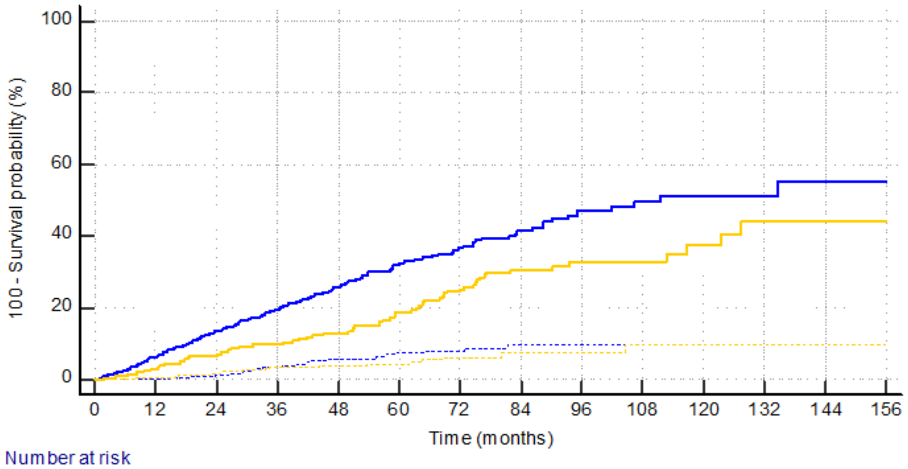

Number at risk

	0	12	24	36	48	60	72	84	96	108	120	132	144	156
Group: Males NLD	881	609	451	354	260	188	133	80	49	30	20	13	11	8
Group: Females NLD	612	422	336	271	213	164	119	84	59	36	22	13	7	6
Group: Males LD	881	609	451	354	260	188	133	80	49	30	20	13	11	8
Group: Females LD	612	422	336	271	213	164	119	84	59	36	22	13	7	6

Probability of non-leukemic death in men
Probability of non-leukemic death in women
Probability of leukemic death in men
Probability of leukemic death in women

Supplementary Figure_3_SF7. *Probability of overall survival of patients stratified by sex, according to different haemoglobin values. This analysis was conducted on retrospective EuroMDS cohort (A), retrospective IWG-PM cohort (B), prospective registry of Spanish MDS Group (GESMD) (C) and prospective Düsseldorf MDS registry (D).*

A. Probability of survival of EuroMDS cohort according to haemoglobin values stratified by sex

Women

Men

Hb≥10 g/dl (men vs women HR 1·55 [CI 1·14-2·10], P<0·0001)

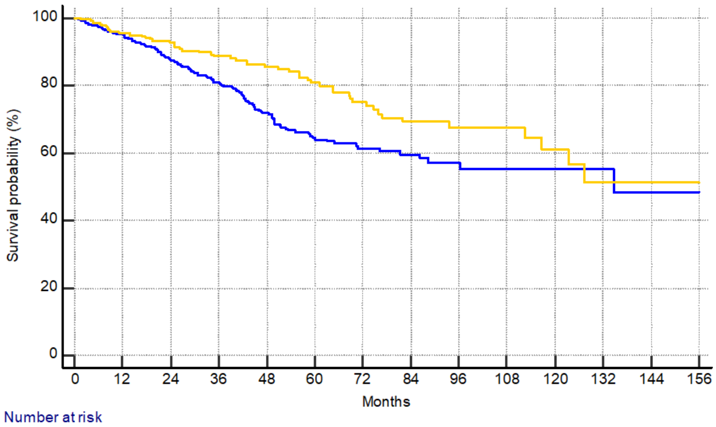

Number at risk

Group: Male	536	366	272	217	164	117	81	55	34	20	12	8	7	5
Group: Female	334	233	194	156	118	96	70	52	39	26	15	9	5	5

8 g/dl≤Hb<10 g/dl (men vs women HR 1·46 [CI 1·08-1·9], P=0·0131)

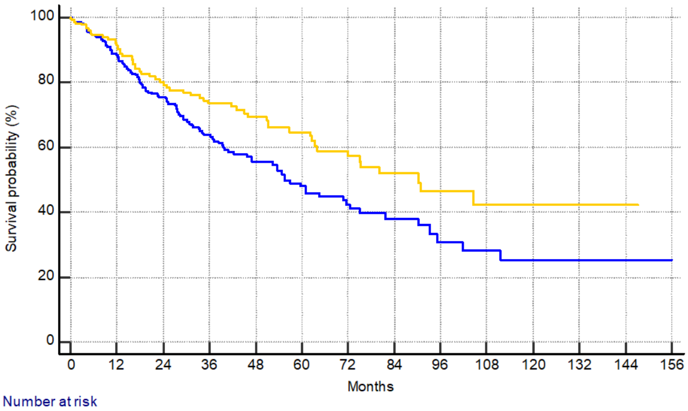

Number at risk

Group: Male	373	203	147	103	71	47	34	22	12	10	7	4	3	2
Group: Female	244	146	106	83	62	49	36	24	12	7	5	3	1	0

Hb<8 g/dl (men vs women HR 1·70 [CI 1·07-2·68], P=0·0242)

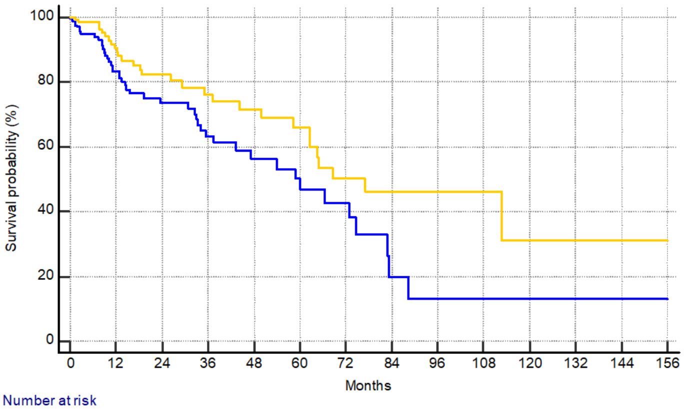

Number at risk

Group: Male	199	82	47	34	21	15	10	3	1	1	1	1	1	1
Group: Female	168	71	46	35	29	21	13	10	8	4	2	1	1	1

B. Probability of survival of IWG-PM cohort according to haemoglobin values stratified by sex

Women

Men

Hb≥10 g/dl (men vs women HR 1·41 [CI 1·09-1·84], P=0·0011)

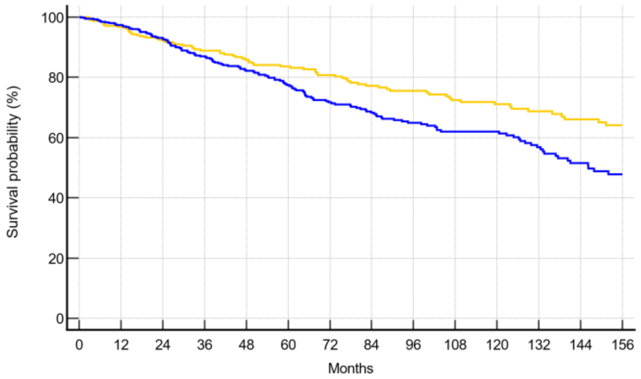

Number at risk

	0	12	24	36	48	60	72	84	96	108	120	132	144	156
Group: F	352	283	242	211	195	178	163	142	130	116	98	81	72	65
Group: M	583	439	363	307	259	222	192	170	141	115	102	80	62	44

8 g/dl≤Hb<10 g/dl (men vs women HR 1·41 [CI 1·12-1·76], P=0·0035)

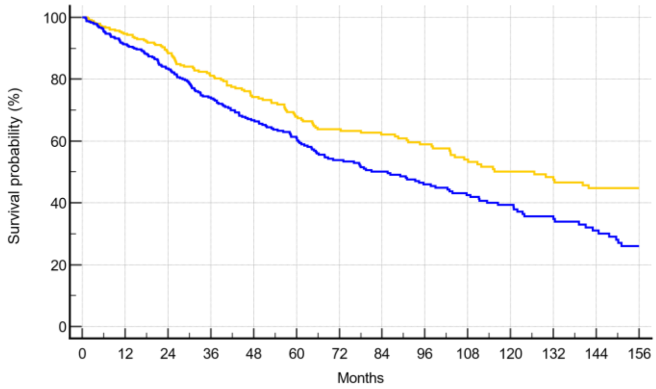

Number at risk

	0	12	24	36	48	60	72	84	96	108	120	132	144	156
Group: F	383	280	229	188	157	137	117	102	89	70	61	54	49	45
Group: M	524	338	271	218	179	151	119	103	86	71	54	41	32	25

Hb<8 g/dl (men vs women HR 1·80 [CI 1·28-2·54], P<0·0001)

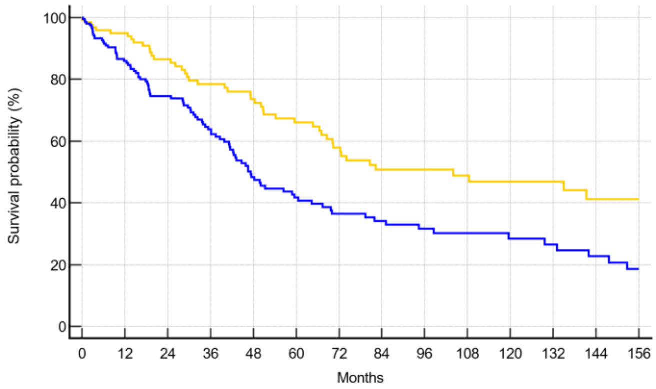

Number at risk

	0	12	24	36	48	60	72	84	96	108	120	132	144	156
Group: F	132	96	77	67	60	51	42	34	32	25	23	20	13	10
Group: M	214	134	104	81	52	42	33	29	24	20	16	14	11	9

C. Probability of survival of Spanish MDS Group (GESMD) cohort according to haemoglobin values stratified by sex

Women

Men

Hb≥10 g/dl (men vs women HR 1·56, [CI 1·39-1·76] P<0·0001)

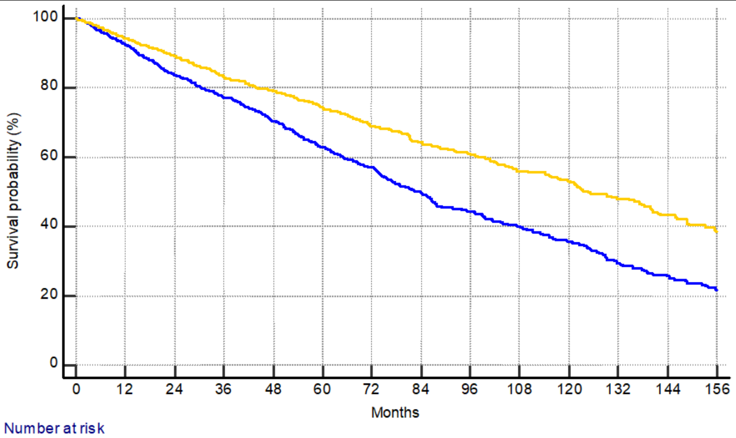

Number at risk

Group	0	12	24	36	48	60	72	84	96	108	120	132	144	156
Group: Male	2189	1615	1223	921	712	547	423	306	240	182	135	92	71	47
Group: Female	1460	1126	893	704	578	477	391	299	248	204	156	112	80	62

8 g/dl≤Hb<10 g/dl (men vs women HR 1·48 [CI 1·29-1·69] P<0·001)

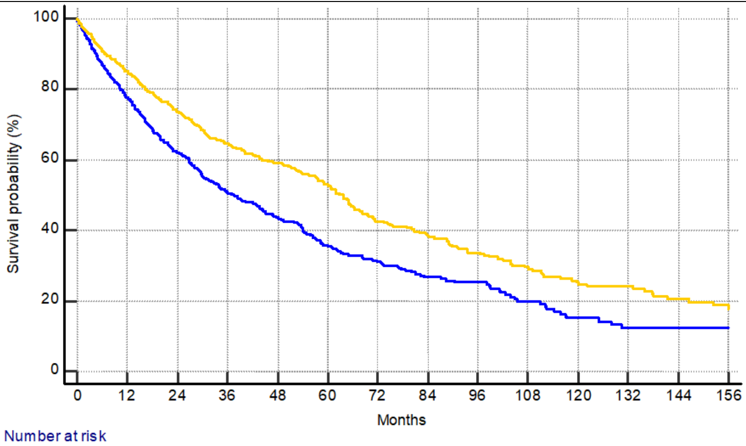

Number at risk

Group	0	12	24	36	48	60	72	84	96	108	120	132	144	156
Group: Male	1142	618	382	256	179	118	86	58	45	31	20	13	12	9
Group: Female	967	627	439	321	249	190	133	101	75	56	45	36	25	18

Hb<8 g/dl (men vs women HR 1·35 [CI 1·19-1·52] P<0·0001)

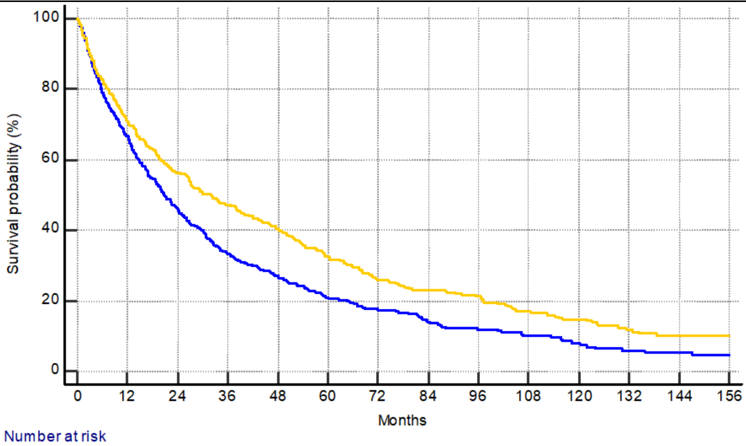

Number at risk

Group	0	12	24	36	48	60	72	84	96	108	120	132	144	156
Group: Male	1084	490	280	164	116	83	59	41	32	21	15	10	9	8
Group: Female	836	447	314	232	178	125	87	68	57	42	35	22	17	16

D. Probability of overall survival of Düsseldorf MDS registry cohort according to haemoglobin values stratified by sex

Women

Men

Hb≥10 g/dl (men vs women HR 1·37 [CI 1·05-1·79] P=0·0182)

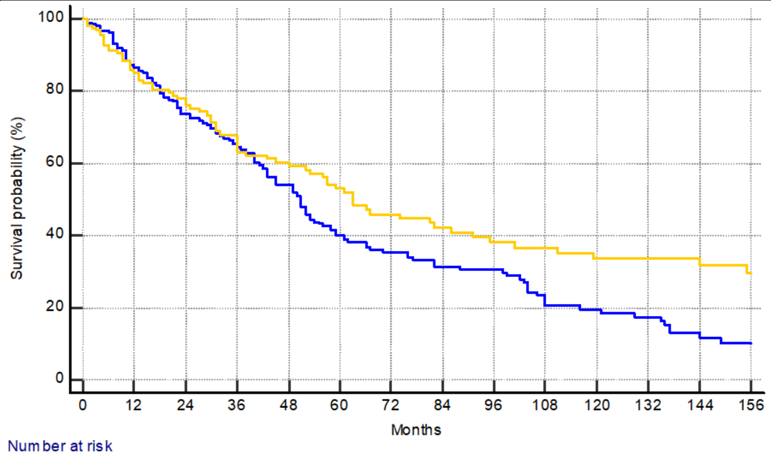

Number at risk

Group	0	12	24	36	48	60	72	84	96	108	120	132	144	156
Group: Male	317	218	162	129	101	63	47	38	35	21	20	16	10	7
Group: Female	168	110	85	66	59	47	39	32	27	24	21	19	17	15

8 g/dl≤Hb<10 g/dl (men vs women HR 1·20 [CI 0·90-1·60] P=0·2131)

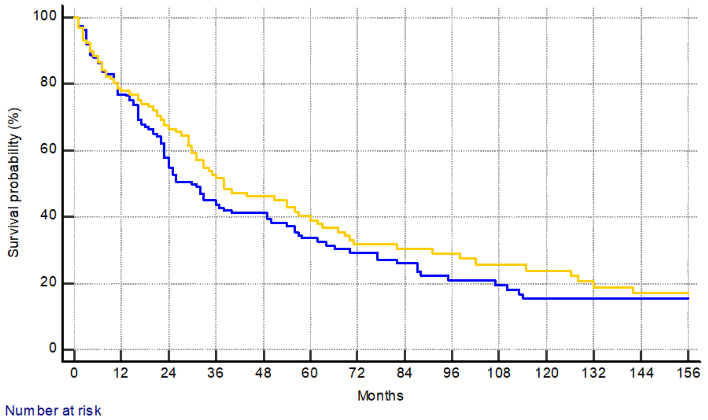

Number at risk

Group	0	12	24	36	48	60	72	84	96	108	120	132	144	156
Group: Male	191	114	76	54	44	33	28	21	16	14	10	9	7	7
Group: Female	135	86	67	47	41	32	24	21	18	16	14	11	8	8

Hb<8 g/dl (men vs women HR 1.63 [CI 1.21-2.19] P=0.0014)

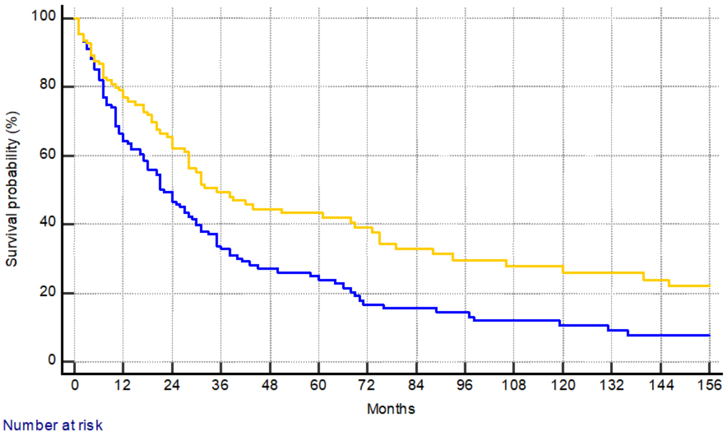

Number at risk

Group	0	12	24	36	48	60	72	84	96	108	120	132	144	156
Group: Male	174	82	54	37	25	21	14	13	12	8	7	6	4	4
Group: Female	124	76	57	42	36	31	26	21	16	15	14	13	12	11

Supplementary Table_1_SF7. *Prognostic impact of different haemoglobin (Hb) value in men and women from EuroMDS and IWG-PM cohorts; HR for probability of overall survival were calculated by using Hb normal value as reference (12-14 g/dl for women and 13-15 g/dl for men). Analyses were adjusted for age. Our analysis showed that anaemia start to have significantly negative prognostic impact below 11 g/dl in men and below 10 g/dl in women. This effect was maintained in multivariable analysis including age, neutrophil and platelet count, % of bone marrow blast and cytogenetics stratified according to IPSS-R criteria (EuroMDS cohort: men HR 2·17[1·23-4·27], P<0·001; women HR 2·51 [1·32-4·42], P<0·0001; IWG-PM cohort: men HR 2·04[1·47-3·66], P<0·0001; women HR 2·29 [1·39-3·84], P<0·0001).*

	**EuroMDS cohort (Men)**				**IWG-PM cohort (Men)**		
**Hb value**	**Hazard ratio**	**CI 95%**	**P value**	**Hb value**	**Hazard ratio**	**CI 95%**	**P value**
Hb <8 g/dl (n=180)	4·39	2·66-7·24	<0·0001	Hb <8 g/dl (n=225)	3·51	2·16-5·70	<0·0001
8 g/dl ≤ Hb <9 g/dl (n=190)	3·99	2·43-6·57	<0·0001	8 g/dl ≤ Hb <9 g/dl (n=275)	2·53	1·55-4·11	<0·0001
9 g/dl ≤ Hb <10 g/dl (n=202)	3·09	1·88-5·08	<0·0001	9 g/dl ≤ Hb <10 g/dl (n=285)	2·06	1·26- 3·37	0·0045
10 g/dl ≤ Hb <11 g/dl (n=189)	2·47	1·49-4·10	<0·0001	10 g/dl ≤ Hb <11 g/dl (n=208)	1·96	1·16- 3·30	0.0112
11 g/dl ≤ Hb <12 g/dl (n=159)	1·65	0·95-2·84	0·0721	11 g/dl ≤ Hb <12 g/dl (n=136)	1·01	0·58-1·76	0·9534
12 g/dl ≤ Hb <13 g/dl (n=80)	1·25	0·67-2·34	0·4732	12 g/dl ≤ Hb <13 g/dl (n=97)	1·11	0·60-2·07	0·7254
	**EuroMDS cohort (Women)**				**IWG-PM cohort (Women)**		
**Hb value**	**Hazard ratio**	**CI 95%**	**P value**	**Hb value**	**Hazard ratio**	**CI 95%**	**P value**
Hb <8 g/dl (n=159)	3·529	1·901 to 6·551	<0·0001	Hb <8 g/dl (n=141)	2·316	1·361 to 3·942	0·0021
8 g/dl ≤ Hb <9 g/dl (n=92)	3·553	1·875 to 6·733	<0·0001	8 g/dl ≤ Hb <9 g/dl (n=188)	2·05	1·225 to 3·433	0·0062
9 g/dl ≤ Hb <10 g/dl (n=161)	2·959	1·609 to 5·442	<0·0001	9 g/dl ≤ Hb <10 g/dl (n=223)	1·719	1·031 to 2·866	0·0384
10 g/dl ≤ Hb <11 g/dl (n=151)	1·791	0·944 to 3·399	0·0723	10 g/dl ≤ Hb <11 g/dl (n=178)	1·221	0·718 to 2·076	0·4632
11 g/dl ≤ Hb <12 g/dl (n=102)	1·348	0·675 to 2·692	0·4032	11 g/dl ≤ Hb <12 g/dl (n=107)	0·913	0·504 to 1·652	0·7625

SUPPLEMENTARY_FILE_8 (SF8) - Personalized prognostic assessment in myelodysplastic syndromes based on demographics, clinical and genomic features

Multistate Cox's proportional-hazards model (coxph)

The association between the overall survival time and possible predictor variables was investigated fitting multistate Cox proportional-hazards models using the survival and *mstate* R packages. Specifically, we considered 3 possible states (Diagnosis, Acute Myeloid Leukemia, AML, and Death) and 3 possible transitions (Diagnosis to AML, Diagnosis to Death, AML to Death). The model was fitted without any proportionality assumption on the baseline hazards, meaning that separate baseline hazards were allowed for the different transitions, and considering transition specific effects.
The analysis of the transition probabilities showed that the transition from AML to Death was particularly fast and highly probable. For this reason, in the following analyses we considered a simplified version of the multistate model in which only two transitions are considered: Diagnosis to AML and Diagnosis to Death, were the second transition also includes subjects that died after passing through the AML state.

Random effects Cox proportional-hazard multistate model (CoxHD)

Further innovative prognostic multistate models were developed fitting the random effects Cox proportional-hazards model developed by Gerstung et al (Nat Genet 49: 332–3340, 2017) and implemented in the R package CoxHD available at (*http://github.com/mg14/CoxHD*). Here, we considered 3 possible states (Diagnosis, AML, Death) and 2 possible transitions: Diagnosis to AML and Diagnosis to Death. In the last case, we also included subjects who died after being affected by AML. As for the previous Cox's model, we did not introduce any proportionality assumption on the baseline hazards, meaning that separate baseline hazards were allowed for the different transitions, and we considered transition specific effects.
Here, the covariates included in the design matrix Z are categorized in groups and the model parameters u_j for each group of variables *g* are assumed to be i.i.d. normally distributed.
Letting the hazard be

$$\lambda = \lambda_0(t)e^{(u^T Z)}$$

where $\lambda_0(t)$ is the baseline hazard of the coxph model; this means that

$$\forall j \in g: \qquad u_i \sim N\left(\mu_g; \sigma_g^2\right), \qquad i.i.d.$$

The shared means are motivated by the assumption that on average the effect of variables belonging to the same category is comparable.
This model can be interpreted as a hierarchical model in which we assume that variables belonging to the same group have the same prior (gaussian) distribution and is equivalent to a ridge penalized model in which the parameters are penalized group by group. The log-likelihood of the model is

$$l(u, \mu, \sigma^2; Z) = l_0(u; Z) - \sum_g \frac{\sum_{j \in g}\left(u_j - \mu_g\right)^2}{\sigma_g^2} = l_0(u; Z) + l_2(u, \mu, \sigma^2; Z)$$

where $u = \{u_j : j = 1, \cdots, p\}$, $u_g = \{u_j : j \in g\}$.

The term $l_0(u; Z)$ is the likelihood of the coxph, while the second term is a sum of ridge penalties resulting from the assumption of normal prior distributions for each group of variables, which penalizes large values of $u_j - \mu_g$(encourages the model parameters to be close to the mean of the corresponding Gaussian distributions) with strength $1/\sigma_g$.

Goodness of fit and model comparison based on the concordance statistic

The goodness of fit of both the coxph and the CoxHD models was evaluated computing the concordance. Concordance is defined as the probability for any two randomly chosen observations that the one with the shorter survival time of the two also has the larger predicted risk score (i.e. is concordant). The concordance C of each model was estimated using the survival R package as

$C = \left(A + \frac{T_P}{2}\right)/(A + D + T_P)$,

where A, D and T_P indicate the number of pairs of observations that are concordant, discordant, and tied on the predictor p but not on the observed data.

Supplementary Figure_1_SF8. *Fraction of explained variation that was attributable to different prognostic factors for non-leukemic death and leukemic death by using Sex-informed Genomic Scoring System on merged EuroMDS and IWG-PM cohorts*

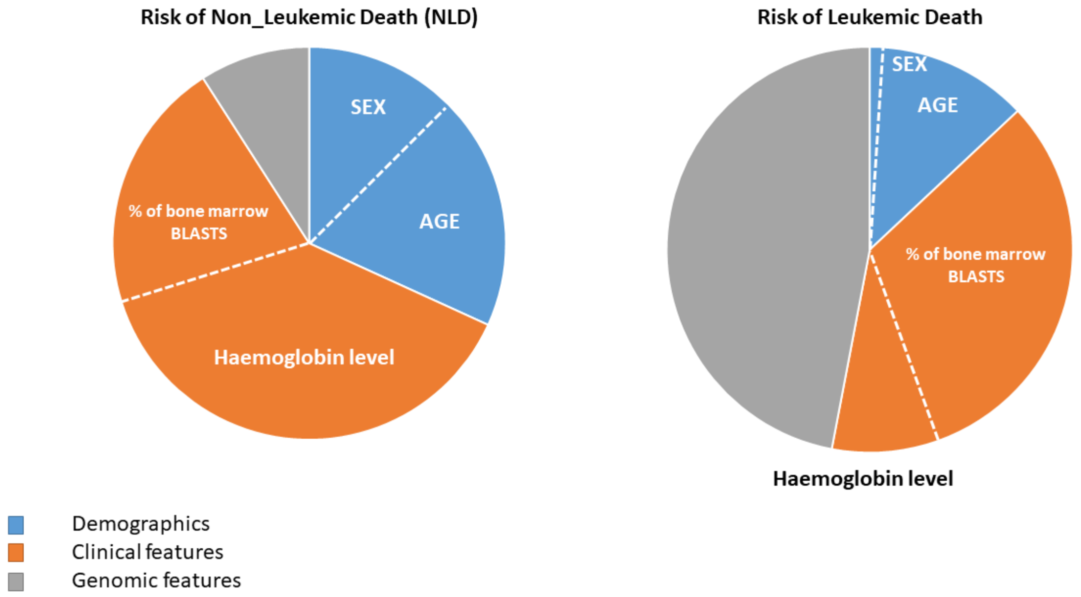

Supplementary Table_1_SF8. *Comparison of concordance (with standard deviation, sd) between IPSS-R categories, IPSS-R and age, and IPSS-R, age and sex on EuroMDS cohort*

**Prognostic model**	**Euro_MDS cohort (n=2,025)**	
	**Concordance**	**sd**
IPSS-R categories (HR 2.15, P<0·0001)	0·68	0·014
IPSS-R categories (HR 2.11, P<0·0001), and age (HR 1.59, P<0·0001)	0·70	0·012
IPSS-R categories (HR 2.13, P<0·0001), age (HR 1.58, P<0·0001) and sex (HR 1.21, P=0·0001)	0·72	0·012

In order to test the improvement of the model due to the introduction of the Sex variable, we used the R function anova.coxph to compute an analysis of deviance for these Cox models considering IPSS-R and age vs. IPSS-R, age and sex. We obtained a p-value of 0.0033, confirming the importance of Sex in the model.
